# Supplementary material for: High Mountain Asian glacier response to climate revealed by multi-temporal satellite observations since the 1960s
Source: Nat Commun. 2021 Jul 5;12:4133. doi: 10.1038/s41467-021-24180-y (PMC8257854; doi:10.1038/s41467-021-24180-y)
Supplement: Supplementary file 1 — Supplementary Materials [file 41467_2021_24180_MOESM1_ESM.pdf]

Supplementary Materials for

High Mountain Asian Glacier Response to Climate Revealed by Multi-temporal Satellite Observations since the 1960s

Atanu Bhattacharya[1,2]*, Tobias Bolch[1]*, Kriti Mukherjee[3], Owen King[1], Brian Menounos[3], Vassiliy Kapitsa[4], Niklas Neckel[5], Wei Yang[6], Tandong Yao[6]

[1]School of Geography and Sustainable Development, University of St Andrews, Scotland, UK.
[2]Department of Remote Sensing & GIS, JIS University, Kolkata, India.
[3]Geography Earth and Environmental Sciences, University of Northern British Columbia, Prince George, Canada.
[4]Institute of Geography, Ministry of Education and Science, Almaty, Kazakhstan.
[5]Alfred-Wegener-Institut Helmholtz-Zentrum fur Polar und Meeresforschung, Bremerhaven, Germany.
[6]Institute of Tibetan Plateau Research, Chinese Academy of Sciences, Beijing, China.

*Correspondence to: atanudeq@gmail.com; tobias.bolch@st-andrews.ac.uk

Supplementary Note

1. Supplementary Note 1: Data
2. Supplementary Note 2: Characteristics of the selected study regions.
3. Supplementary Note 3: Detailed regional and glacier specific changes.
4. Supplementary Note 4: Elevation change data gap filling methodology
5. Supplementary Note 5: Supplementary References

Supplementary Figures

Supplementary Figure 1. (a) Distortion pattern of Corona KH-4 images, (b) Forward and Aft raw images and (c) Orthorectified image and hillshade of the Corona DEM of the Ak Shirak region.

Supplementary Figures 2-9. Glacier surface elevation differences of the available time periods for the eight regions.

Supplementary Figures 10-17. Off-glacier surface elevation differences of the available time periods for the seven regions.

Supplementary Figure 18. Comparison of ERA5 Land data with the data of (A) Tuyuksu (for Northern Tien Shan), (B) Tien Shan (for Ak-Shirak) and (C) Bange (for Purogangri Ice Cap) weather stations.

Supplementary Figure 19. Comparison of ERA5 Land data with the data of (A) Dangxiong (for Western Nyainqentanglha), (B) Nielamu (for Poiqu region) and (C) Pulan (for Gurla Mandhata) weather stations.

Supplementary Figure 20. Comparison of ERA5 Land data with the data of Tashikuergen (for Muztagh Ata Massif) weather stations.

Supplementary Figure 21. Summer temperature and summer precipitation anomaly of ERA 5 Land gridded climate data for all regions.

Supplementary Figure 22. Winter temperature and winter precipitation anomaly of ERA 5 Land gridded climate data for all regions.

Supplementary Figure 23. Annual temperature and annual precipitation anomaly of ERA 5 Land gridded climate data for all regions.

Supplementary Figure 24. ERA5 Land gridded climate data for all investigated region (SP = Solid precipitation, LP = Liquid precipitation)**.**

Supplementary Figures 25-32. Comparison with mass balances derived by other studies for individual glaciers in each region.

Supplementary Figure 33. Summer temperature and winter precipitation anomalies of the available weather station data along with geodetic glacier mass balance estimates for each of the study sites and time periods.

Supplementary Figure 34. Summer temperature and summer precipitation anomalies of the available weather station data along with geodetic glacier mass balance estimates for each of the study sites and time periods.

Supplementary Figure 35. Surface elevation difference based on ASTER (acquisition date: 09.11.2012) and TanDEM-X (26.01.2012) DEMs for the Purogangri Ice Cap (PIC) region and based on Pléiades (26.10.2013) and TanDEM-X (28.10.2013) DEMs for the Gurla Mandhata

region. The difference provides information about magnitude and characteristics of the X-band radar penetration depth.

Supplementary Figure 36 Distribution of elevation difference data over the full study period in Ak-Shirak and Northern Tien Shan.

Supplementary Tables

Supplementary Table 1. Details about the remotely sensed data used in this study (MB: Glacier Mass Balance; Mapping: Glacier Mapping).

Supplementary Tables 2-9. Elevation change (ΔH) and mass budget (MB) of selected glaciers and all glaciers in the seven study regions for the different time periods.

Supplementary Tables 10-16. Area change (ΔA) and length change rate (ΔL) of the selected glaciers and all glaciers in the seven study regions for the different time periods.

Supplementary Table 17. Summer, winter and annual temperature, precipitation and annual solid precipitation for different time periods based on ERA5 Land Climate data.

Supplementary Table 18. Characteristics of the available and analyzed weather stations for the seven study regions.

Supplementary Table 19. Correlation matrix between the High Asia Refined Analysis (HAR V2) water vapor and precipitation data with ERA5 Land and corresponding weather station data for Northern Tien Shan, Gurla Mandhata and Muztagh-Ata regions.

Supplementary Tables 20-27. Comparison of glacier mass balances with other available studies and measurements in the seven study regions.

Supplementary Table 28**.** Comparison of mass balance of surge type and non-surge type glaciers in Ak-Shirak and Muztagh-Ata regions.

Supplementary Table 29. Comparison of Elevation difference in different time period using regional mean hypsometry and regional median hypsometry gap filling methods for Ak-Shirak region

Supplementary Table 30. Mean and median elevation change considering outlier outside ±100 m or ±150 m for Ak-Shirak and Northern Tien Shan region

Supplementary Note 1

1. Data

1.1. Satellite Data

1.1.1. Data from the 1960s and 1970s: Corona KH-4 and Hexagon KH-9 data

Corona and Hexagon imagery was originally used for reconnaissance and to produce maps for U.S. intelligence agencies. Corona KH-4 satellites collected more than 860,000 images of the Earth's surface between 1960 and 1972[1]. The KH4 archives were declassified in 1995[1,2]. Corona KH-4 images have the potential to yield surface elevation data due to their stereo capabilities[3,4]. Corona KH-4 had a dual panoramic camera mounted on the satellite which produced high resolution images of up to 1.8 m for KH-4B in nadir[5]. The dual panoramic Corona KH-4 camera had a focal length of 609.60 mm and its spatial resolution varied in the scan direction of the film from 3-7.6 m (KH-4), 2.7-7.6 m (KH-4A) and 1.8-7.6 m (KH-4B)[6]. Corona images covered approximately 210×15 km^2 (KH-4), 132×17 km^2 (KH-4A) and 188×14 km^2 (KH-4B) ground area with a flight height of ~200 km[6,7] and the cameras were tilted ± 15^0 with in both forward (FWD) and backward (AFT) directions. Therefore, Corona KH-4 camera provided a 30^0 convergent angle along the flight direction[5].

Several geometric distortions (Supplementary Figure 1) are prevalent within Corona KH-4 images due to the application of a panoramic camera and the forward movement, which makes reconstructing 3D surfaces complex especially at the edges of the image stripes. Moreover, due to the absence of calibration and ancillary data such as fiducial coordinates, principal point coordinates, lens distortion coefficient, position, velocity vector and altitude angle, conventional photogrammetric methods are not suitable to generate DEMs from Corona images.

The Hexagon KH-9 mapping camera system, operated between 1973-1980, used a 12-inch terrain lens system with a view angle of 80 degree[8]. An area of approximately 250×125 km^2 is covered by one KH-9 scene with a film resolution of 88-line pairs per mm and a focal length of 30.5 cm[9]. Hexagon KH-9 images distortion varies from 0.91 pixels to 4.91 pixels and originates from the development of the film and almost three decades of storage[9]. The frame camera design of KH-9 provided a precise reseau grid (1081 reseau crosses) along with four fiducial marks which can be used to reconstruct the image geometry.

1.1.2. Data from the 2000s and 2010s: ASTER, SPOT6/7 GeoEye, Pléiades, TanDEM-X

The ASTER sensor, onboard NASA's Terra satellite, started operating from December 1999. The ASTER sensor collects 15m visible/near-infrared (VNIR) spatial resolution images and has along-track stereoscopic capabilities. Cross-track capabilities of ASTER have been achieved by rotating two VNIR telescopes up to 24^0 and additionally, along-track stereoscopy is used for radiometric calibration[10].

SPOT-6 and SPOT-7, two optical remote sensing spacecraft of identical characteristics and capabilities of acquiring data of high resolution, were launched 2012 and 2014 respectively by Airbus Defense and Space (former name: EADS's Astrium Service) in order to continue sustainable wide-swath high resolution observation services[11]. Both SPOT-6 & SPOT-7 satellites were designed for same sun-synchronous orbit with an inclination angle and altitude of 98.2^0 and 694 km respectively[12]. The panchromatic (Pan) and multi-spectral (RGB) images are acquired simultaneously with along-track stereo capability and a spatial resolution of 1.5 m and 6 m respectively.

Pléiades (PLE) is an optical earth observation system for both civilian and military purpose developed by CNES as part of the intergovernmental agreement ORFEO between France and Italy. Pléiades1A and Pléiades 1B, launched on 17 December 2011 and 2 December 2012 respectively, are two similar but not identical satellites that are flying at an altitude of 694 km and capturing images in stereo and tri-stereo mode. The nadir resolution of the panchromatic and four multi-spectral band (RGB and Near infrared (NIR)) is 0.7 m and 2.8 m, respectively which are resampled to 0.5 m and 2.0 m. The image swath of Pléiades is 20 km at nadir, and by adjusting its viewing angle of up to 47^0 it can enlarge the width of the swath[13,14].

Similar to Pléiades, the GeoEye-1 satellite, launched in 2008, is also capable to acquire data in single pass stereo mode with panchromatic and four multi-spectral bands (blue, green, red and near-infrared) of resolution 0.41 m and 1.65 m, resampled to 0.5 m and 2.0 m respectively.

TerraSAR-X (TSX) and TanDEM-X (TDX) are two almost identical radar satellites operated by the German Aerospace Centre (Deutsches Zentrum für Luft- und Raumfahrt, DLR). TSX was launched in June 2007 followed by twin satellite TDX in June 2010. Both satellites are equipped with a X-band Synthetic Aperture Radar (SAR) system and fly in close formation to act as a single pass SAR interferometer. The main aim of the TDX mission is to generate a globally consistent

Digital Elevation Model (DEM) with a 12×12 m grid with a vertical accuracy of < 2 m, fulfilling the high DTED-3 specification[15,16].

1.2. Climate data

1.2.1 Weather station data

For each of the seven regions we selected the closest and most representative existing weather station with long-term records of temperature and precipitation data (Supplementary Table 18). The weather stations are typically located at lower elevation than the glaciers but give an indication of the climatic conditions and trends of the regions and allow comparisons to the gridded climate data. Some weather stations (e.g., Tien Shan and Tuyuksu) are located relatively close to glacier tongues but others are found lower in the valley (e.g., Pulan). The closest station to PIC is located on the Tibetan Plateau, but about 300 kilometres away (Bange). We did not use the data from Tuotuoh station which is closer to PIC but only contains short term data.

1.2.2. ERA5 Land

ERA5 Land data is a global land surface dataset at 0.1 degree (9 km) spatial resolution and is available from January 1981 to 2-3 months before present at an hourly time step. This is the land component of the ERA5 data but is more accurate for land applications after a series of improvements. Uncertainty of the ERA5 data is provided (at 60 km horizontal and 3 hr temporal resolution) by the Ensemble of Data Assimilations (EDA) system, mostly accounting for random errors. The uncertainties are usually less for recent periods than earlier periods. We have used the '2m temperature' and 'total precipitation' data from 1982 until 2019 for all the seven regions.

For Northern Tien Shan, we have used data from 3 grid points with a mean elevation of 2800 m a.s.l. whereas, the average ELA was 3800-4000 m a.s.l. Mean elevation (4210 m a.s.l.) estimated from 12 grid points of Ak-Shirak was similar to the average ELA (4200-4300 m a.s.l.) of this region. The Purogangri Ice Cap (PCI) was covered by 6 grid points with an average elevation of 5431 m a.s.l. Maximum grid points were selected for western Nyainqentanglha (36 points) and Poiqu (38 points) regions. The average elevation of ERA5 Land data for Poiqu and Western Nyainqentanglha were significantly lower than the mean ELA of the glacierised areas (Supplementary Table 19). Similarly, the average elevation of the grid points, 5019 m a.s.l and 4481 m a.s.l for Gurla Mandhata (12 grid points) and Muztagh Ata Massif (25 grid points)

respectively, were lower than the mean ELA of these regions. We have considered May to October as summer and November to April as the winter season. We have calculated the solid and liquid precipitation by considering that any precipitation below 0^0 is solid and otherwise it is liquid precipitation (Supplementary Figures 21-24).

1.2.3. High Asia Refined Analysis (HAR V2)

The regional Numerical Weather Prediction (NWP) model used to generate the HAR dataset[17] is the Advanced Research Weather Research and Forecasting model (WRF-ARW). The HARV2 dataset consists of consecutive reinitialised model runs of 36 hr time integration. The first 12 hr from each run are considered as spin-up while the remaining 24 hr of the model output provides a daily, 11-year long time series in initial version of the HAR data[18], which has been extended to 2018 in the latest release. Moreover, HARV2 covers also a larger area and includes the entire Tien Shan.

1.2.4. Comparison of ERA5 Land with weather station data and HARV2 data

We found only a weak correlation (Supplementary Table 19) between HAR V2 water vapour flux and the precipitation of ERA5 Land (r^2 = 0.01 to 0.58 and p = 0.1 to 0.02 for all three study regions) and weather station data (r^2 = 0.01 to 0.44 and p = 0.2 to 0.5 for all three study regions). Thus, we do not compare this dataset against our mass balance time series. However, we observed a strong correlation of summer temperature anomalies between gridded ERA5 Land and meteorological data for all the regions (Supplementary Figures 18-20) but, precipitation anomalies for most of the regions, except for winter precipitation of Gurla Mandhata (r^2 = 0.82 and p = 0.01), showed very little agreement. Moreover, the deviations for the precipitation anomalies can be attributed to the fact that precipitation variability is much larger and most of the stations are located at lower elevations than the average of the selected grid cells. The significant deviation is evident for the summer precipitation at the Tien Shan weather station (R^2 = 0.6 & p = 0.5) for the period around 1990 where measurements show a strong decrease while ERA5 an increase (Supplementary Figure 16). It is likely that the in-situ measurements were erroneous as no other nearby weather station (e.g. Karakol, Naryn) shows such a precipitation decrease during this period. Moreover, the station was relocated and newly equipped in 1997 and thereafter there is close agreement between the weather station data and ERA5 land data. The amount of precipitation of Pulan weather station shows the highest variation in-spite of a good correlation of winter precipitation

(R^2 = 0.82 & p = 0.01) (Supplementary Figure 17 (C). The station measures overall very little precipitation and the deviation from the mean is also very small as compared to the gridded data. This is probably due to the fact that the station is located in the dry valley bottom at an elevation of 3900 m a.s.l. while the gridded data of more representative for high elevation precipitation (mean elevation 5020 m a.s.l.).

Supplementary Note 2

2. Characteristics of the selected study regions

2.1 Northern Tien Shan: The mountain ranges of Ile (Zailiskiy) Alatau and Kungöy Ala-Too constitute a major part of Northern Tien Shan and are located in Central Asia at the border between Kazakhstan and Kyrgyzstan. Our data specifically covers Ulken (Big) Almaty Valley, Kishi (Small) Almaty Valley and Left Talgar Valley of Ile (Zailiskiy) Altau in Kazakhstan. The region has a semi-continental climate, with greater precipitation on windward northern slopes compared to leeward southern valleys[19]. Precipitation is generally lowest in the winter due to the Siberian anticyclone and greatest in early summer[19,20]. ERA5 Land reanalysis climate data also showed a lower mean winter precipitation (~ 421 mm) compared to mean summer precipitation (~ 902 mm) during the observation periods (Supplementary Table 17). Moreover, mean summer temperature is above zero (~4.25^{0}C) at an average elevation of ~ 2800 m a.s.l. The ELA for the northern slope of the entire northern Tien Shan is approximately 3800 m a.s.l and slightly higher (3900-4000 m a.s.l) on the southern slope[19]. We have considered 48 glaciers constituting ~ 51 km^2 of glacierized area.

2.2 Ak-Shirak range: The Ak-Shirak mountain range is located in Central Tien Shan in Kyrgyzstan. The region typically displays a continental dry and cold climate. ERA5 reanalysis data during the observation period showed that annual mean precipitation is about 607 mm. While weather station data from the Tien Shan station (located above 3600 m a.s.l.)[21] and the ERA5 data show higher precipitation during the summer months, in-situ investigations at glacier 354 indicate slightly higher winter accumulation[22]. The annual air temperature at 4210 m a.s.l. is about -9.7°C and varies from –17.2°C in winter to –2.2°C in summer, based on the ERA5 reanalysis data (Supplementary Table 17). The ELA is located between 4200 and 4300 m a.s.l. We have investigated 177 glaciers with a glacierized area of 365 km^2.

2.3 Purogangri Ice cap (PIC): PIC, is located in the north-central part of the Tibetan Plateau and is dominated by continental climate and is less influenced by the westerlies and the Indian monsoon[23]. Annual mean precipitation is ~568 mm due to cold and relatively dry continental climate and around 80–86% (~ 487 mm) of this precipitation occurs in the summer months (Supplementary Table 17). However, the average summer temperature at an elevation of 5430 m a.s.l, a parameter associated mostly with glacier melt[24], is below 0^0 C (−2.7^0 C). Meteorological

data at Tuotuohe station, 130 km from the Dongkemadi glacier, also shows a mean annual temperature of -2.7^{0}C, and the highest monthly average temperatures occurred during June–September[25]. Our time series of observations covers 37 PIC glaciers which cover nearly 344 km^2 of glacierized area. with a mean ELA of 5750 m a.s.l[26].

2.4 Western Nyainqentanglha: The Western Nyainqentanglha mountain range is situated in the south-east of the central Tibetan Plateau. The area is influenced by both the continental climate of Central Asia and Indian Monsoon system[27,28]. The approximate ELA is reported as 5770 ± 5 m a.s.l[28]. The mean annual air temperature estimated from ERA5 Land reanalysis data at an average elevation of 5095 m a.s.l, is −3.8^{0}C (Supplementary Table 17), however the mean summer temperature is well above zero (~2.3^{0}C). The mean annual precipitation of this region (~860 mm) predominantly occurs in the summer (~764 mm), which is ~89% of total precipitation. A similar mean annual precipitation (700-900 mm) is also reported[29] for Xibu Glacier which is situated close to Mt. Nyainqentanglha (7162 m a.s.l., the highest mountain of the range). Moreover, Nam Co meteorological station data also shows that 90% of mean annual precipitation is measured in the warm season from June to September[29]. Our analyses included 115 glaciers in Western Nyainqentanglha which have an area of ~168 km^2.

2.5. Poiqu region: The Poiqu River is a trans-boundary river that originates in the Tibetan Plateau and the southern slopes of the central Himalayas and flows southward into Nepal. We have included also the neighboring Langtang valley in our analyses as there is better data availability for this sub-region. The climate of the region is largely controlled by the Indian monsoon and is categorized by a humid subtropical mountain climate[30]. ERA5 Land reanalysis data suggests the mean annual air temperature is -2.4°C and the summer temperature is above zero degrees (~3.3°C) at an average elevation of 4970 m a.s.l (Supplementary Table 17). The region receives ~1709 mm mean annual precipitation and 81% of this precipitation falls in the summer (monsoon) months. The ELA of glaciers is located around 5600 m[23]. We investigated 179 glaciers in this region which constitute ~ 490 km^2 of glacierized area.

2.6. Gurla Mandhata (Naimona'Nyi): Gurla Mandhata (or Naimona'nyi) is an isolated dome-shaped massif in the central-west Himalaya (Nalakankar Himal) in Burang County, Ngari Prefecture. The Indian summer monsoon provides the bulk (~70%) of the annual mean precipitation (~ 5219 mm) (Supplementary Table 17) but winter Westerlies also provide

precipitation to the area. The resulting two precipitation peaks of approximately equal magnitude reflect a typical pattern in the transition zone controlled by both climate systems[31]. Low summer temperatures (~0.11°C at 5019 m a.s.l.) and substantial winter precipitation could be a major reason for weak glacier melt in this region. A field study[23] measured a high altitude of specific ELAs for Gurla Glacier (G081317E30454N) at 6300 m for 2004/06, 6480 m for 2007/08, 6200 m for 2008/09 and 6150 m for 2009/10. We have considered 50 glaciers which constituted ~ 73.3 km^2 of glacierized area.

2.7. Muztagh Ata Massif: The Muztagh-Ata massif, located in most eastern part of Pamir and west of the Taklamakan Desert, is one of the coldest environments in low and mid-latitude regions of Hindu Kush Himalaya (HKH), Muztagh-Ata is dominated by cold and semi-arid continental type climate and is principally influenced by mid-latitude westerlies[23]. The mean annual temperature and mean annual precipitation is estimated as -8.8^0 C and ~415 mm at 4481 m a.s.l respectively from ERA5 Land reanalysis data (Supplementary Table 17). Low summer temperatures (-0.7^0 C) and substantial mean winter precipitation (~308 mm or 73% of total precipitation) could be a major reason for weak glacier melt in this region. The ELA for the period 1973-2013 was estimated to be ~5285 m a.s.l by[32]. We investigated 166 glaciers in this region which represented ~ 348 km^2 of glacierized area.

Supplementary Note 3

3. Detailed regional and glacier specific changes

In this section, we present detailed results for all seven study regions and for selected glaciers. We have also presented more information in Supplementary Tables 2-17 and Supplementary Tables 20-27.

3.1. Northern Tien-Shan: Total glacierized area reduced by 15.9 ± 1.9 km^2 at a mean rate of 0.3 ± 0.03 km^2a^{-1} from 1964 to 2020 in Northern Tien-Shan. The maximum rate of glacier area loss occurred over the last two decades (2000-2020) at a mean rate of 0.6 ± 0.03 km^2a^{-1}, compared to 0.1 ± 0.02 km^2 a^{-1} from 1964 to 2000 (Supplementary Table 10). Over the entire observation period (1964-2020), the mean glacier thickness change was estimated as -26.12 ± 6.7 m (-0.47 ± 0.12 m a^{-1}) which amounts to 1.16 ± 0.21 Gt (0.02 ± 0.01 Gt a^{-1}) of ice mass loss or an equivalent annual mass budget rate of -0.40 ± 0.09 m w.e.a^{-1} (Supplementary Table 2). The glacier mass loss rate significantly increased from -0.18 ± 0.11 m w.e.a^{-1} to -0.49 ± 0.13 m w.e.a^{-1} from 1964-1971 and 2016-2020 respectively. Using the time series of ASTER DEM based elevation change data derived by Brun et al. (2017)[33], we estimated a mean mass balance of -0.41 ± 0.20 m w.e.a^{-1} for the same glaciers in Northern Tien-Shan from 2000-2016. Using the DEMs generated in this study, we estimate a mass loss rate of -0.43 ± 0.09 m w.e.a^{-1} from 2000-2012 and -0.45 ± 0.13 m w.e.a^{-1} from 2012-2016 (Supplement Table 20). Tuyuksu (G077081E43044N) glacier, which has been closely monitored since the 1960s by field measurements, showed a similar trend in ice loss, from -0.30 ± 0.11 m w.e.a^{-1} (1964-1971) to -0.48 ± 0.13 m w.e.a^{-1} (2016-2020) with a mean rate of -0.43 ± 0.09 m w.e.a^{-1} during the entire period (Supplementary Tables 2 & 20). Further comparisons between individual glacier mass loss rates using data derived in the study and using elevation change data generated by Brun et al. (2017)[33] are shown in Supplementary Table 20.

3.2. Ak-Shirak Range: Substantial reductions of glacierised area occurred in the Ak-Shirak range from 1964 to 2019 (38.4 ± 1.4 km^2 at a mean rate of 0.7 ± 0.03 km^2 a^{-1} or 0.19 ± 0.01% per year) (Supplementary Table 11). The mean rate of glacier surface down-wasting was estimated to be -26.1 ± 5.3 m (-0.47 ± 0.09 m a^{-1}) with an equivalent annual mass budget rate of -0.40 ± 0.07 m w.e. a^{-1} from 1964-2019. The region lost 8.09 ± 1.08 Gt (0.15 ± 0.02 Gt yr^{-1}) of ice from 1964 to 2019. The rate of mass loss decreased substantially in the period 2002–2019 (-0.31 ± 0.15 m w.e.a^{-1}) when compared to the time period 1964-2002 (-0.43 ± 0.13 m w.e.a^{-1}) (Supplementary Tables 3

and 21). Slight contrasts in estimated rates of mass loss exist when comparing our results with those of other regional studies which examined ice mass loss over different time periods. From 1964-1980, we estimate a mean mass loss rate of -0.28 ± 0.16 m w.e.a^{-1}; slightly lower than that estimated by Goerlich et al. (2017)[7]. From 1973-2002 we estimate a mean mass loss rate of -0.47 ± 0.12 m w.e.a^{-1}, compared to -0.51 ± 0.36 m w.e.a^{-1} from 1975-1999 estimated by Pieczonka and Bolch (2015)[8] and -0.59 ± 0.31 m w.e.a^{-1} estimated by Aizen et al. (2007)[40] from 1977-1999. Using data derived by Brun et al. (2017)[33] we derived a mean mass loss rate of -0.32 ± 0.20 m w.e.a^{-1} from 2000-2016, compared to -0.31 ± 0.14 m w.e.a^{-1} using our DEMs.

Several surge-type glaciers, namely Davidov, Sary Tor North, Basimjannij, Kaindy, Kara Say North have previously been reported [7,34] in Ak-Shirak. We do not examine the behavior of Davidov glacier as the main part of its tongue was removed starting from 1997 by the Kumtor goldmine company[35]. Our time series of observations shows that the lower reaches of Kaindy glacier advanced (38.9 ± 1.5 m a^{-1}) and thickened from 1964 to 1973 (Supplementary Tables 3 and 11). Overall, the Kaindy glacier showed a slight mass loss (-0.18 ± 0.16 m w.e.a^{-1}) during the active phase of the surge (Supplementary Table 3). The initial advance of the Davidov glacier (12.0 ± 1.5 m a^{-1}) was slower than the Kaindy glacier from 1964 to 1973, but its terminus advanced until 1980 at an increased rate (25.5 ± 1.9 m a^{-1}). Similar to Kaindy glacier, Davidov glacier also experienced a surface thickening of its tongue from 1964 to 1973 (Supplementary Figure 3). In direct contrast, Kara Say North (-23.9 ± 0.4 m a^{-1}) and Petrov glaciers (-28.4 ± 0.4 m a^{-1}) showed the strongest retreat rates over our entire study period. The accelerated mass loss of the Petrov glacier might be due to the constant expansion (240%) of moraine dammed glacier lake throughout our study period (1964-2019). The surge-type Besimjannij glacier[34] showed complex behavior including extreme retreat from 1964 to 2002 (-33.3 ± 1.5 m a^{-1}) and then advance at a rate of 14.1 ± 2.8 m a^{-1} until 2017 (Supplementary Table 11). We measured substantial ice loss (-0.54 ± 0.10 m w.e.a^{-1}) between 1980 and 2002 in Ak-Shirak, which was not coincident with a marked increase in temperature or a decrease in precipitation in either the ERA5 Land or Tien Shan weather station data.

The estimated mass budgets for the all eight selected glaciers varied from -0.43 ± 0.07 m w.e.a^{-1} to -0.68 ± 0.07 m w.e.a^{-1} between 1964 and 2019, although higher mass loss rates were evident in intervening periods. Besimjannij (-0.84 ± 0.16 m w.e.a^{-1}), Petrov (-0.59 ± 0.16 m w.e.a^{-1}) and Davidov glaciers (-0.60 ± 0.16 m w.e.a^{-1}) in particular had the highest overall mass loss rates from 1964 to 1973. Interestingly, Davidov glacier also experienced a slight mass gain (+0.22 ± 0.18 m

w.e.a^{-1}) in the following time period (1973-1980) (Supplementary Table 3). Bordu South glacier in this region showed a balance budget (+0.07 ± 0.16 m w.e.a^{-1}) during the same period (1964-1973).

3.3. Purogangri Ice Cap: PIC lost 0.6 ± 0.08 km^2 a^{-1} of its total glacierised area from 1969-2019 (Supplementary Table 12), which equates to a total ice mass loss of 2.51 ± 1.32 Gt (0.05 ± 0.03 Gt yr^{-1}). We measured moderate ice mass loss before 2000 (-0.22 ± 0.07 m w.e.a^{-1} for 1969-2000) when compared to recent years (-0.05 ± 0.03 m w.e.a^{-1} for 2000-2019), despite the PIC being located in and relatively dry region (Supplementary Table 4). The mean rate of glacier area changes from 2000-2019 (-0.4 ± 0.02 km^2 a^{-1}) was similarly dampened. We noted glacier surface elevation increases in the interior of the ice cap alongside glacier terminus thinning (Supplementary Figure 4). Possible surge activity, indicated by positive elevation differences (0.62 ± 0.36 m a^{-1}) along with a strong advance of the tongue (567 ± 21.2 m a^{-1}) was identified for glacier G089227E33891N from 2000-2012. The mass balance of individual glaciers at PIC was highly heterogeneous otherwise. We estimate a moderate rate of mass loss (-0.15 ± 0.07 m w.e.a^{-1}) for the last five decades (1969-2019). Over shorter time periods, our mass loss estimates are generally comparable to other geodetic studies. From 1975-2000, we estimate a mean mass loss rate of -0.22 ± 0.07 m w.e.a^{-1} which is comparable to the estimate of -0.21 ± 0.07 m w.e.a^{-1} made by Lei et al. (2012)[36]. From 2000-2012 we measured close to balanced glacier mass budgets (-0.03 ± 0.07 m w.e.a^{-1}) as did Neckel et al. (2013)[37] (-0.04 ± 0.02 m w.e.a^{-1}).

3.4. Western Nyainqentanglha: Glaciers in western Nyainqentanglha experienced continuous retreat and sustained surface lowering throughout the observation period (1968-2019), although the variability in mass loss rates between individual glaciers has been high. The area of the investigated glaciers reduced at a mean rate of -0.5 ± 0.02 km^2 a^{-1} (-24.7 ± 1.9 km^2 or -0.30 ± 0.02% a^{-1}) from 1968-2019 (Supplementary Table 13). The mean rate of glacier surface lowering in the western Nyainqentanglha was 0.38 ± 0.12 ma^{-1}, equivalent to an annual mass loss rate -0.32 ± 0.09 m w.e.a^{-1}, from 1968 to 2019 (Supplementary Table 5). The north-west flowing Zhadang glacier (G090639E30472N), which is also the site of glaciological mass balance investigations, lost considerable mass (-0.62 ± 0.15 m w.e.a^{-1}) in recent years (2012-2018). The mass loss rate of the large (~ 28 km^2 in 1968), debris-covered, south-east flowing Xibu glacier (G090600E30388N) was -0.28 ± 0.09 m w.e.a^{-1} from 1969-2019. The mass loss rate of several clean ice glaciers in Western Nyainqentanglha (G090521E30390N- -0.53 ± 0.09 m w.e.a^{-1}, G090639E30472N- -0.46

± 0.09 m w.e.a^{-1} and G090550E30416N- -0.36 ± 0.09 m w.e.a^{-1}), was higher than the areas debris cover glaciers (G090618E30355N- -0.25 ± 0.09 m w.e.a^{-1} and G090600E30388N- -0.32 ± 0.09 m w.e.a^{-1}) although our sample size is small in this region (Supplementary Tables 5 & 23).

The results of similar geodetic studies tend to agree with our findings of consistently increasing ice loss rates from Western Nyainqentanghla glaciers since the mid-1970s. Zhou et al. (2018)[38] estimate a mean mass loss rate of -0.25 ± 0.15 m w.e.a^{-1} from 1976-2000, akin to our estimate of -0.24 ± 0.13 m w.e.a^{-1} from 1976-2001. Similarly, our estimates of mass loss from 2001-2012 (-0.41 ± 0.11 m w.e.a^{-1}) and 2012-2018 (-0.47 ± 0.15 m w.e.a^{-1}) are close to the mass loss rate derived using the elevation change data from Brun et al. (2017)[33] (-0.42 ± 0.23 m w.e.a^{-1}). Studies concentrating on shorter time periods since the millennium (Supplementary Table 23), particularly those using ICESat data, have estimates both higher[39] and lower[40] mass loss rates from the region. The shorter time period (2003-2008) of the ICESat data may therefore not be representative of the overall ice loss trajectory in this region.

3.5. Poiqu region: Our time series over the broader Poiqu catchment begins with Hexagon KH-9 declassified data from 1974 and for other time periods we used ASTER (2004) and Pleiades (2018) dataset. However, to improve our understanding of temporal behavior of glaciers in this region, we have also examined the mass balance of glaciers in the Langtang valley adjacent to Poiqu catchment at higher temporal resolution (1964-20019) as more appropriate data was available (Supplementary Table 1), A decrease in glacier area was evident for all the glaciers throughout the observation period and overall area loss was 39.3 ± 1.5 km^2 from 1974 to 2018 (Supplementary Table 14). The rate of areas loss was higher over our most recent time period (2004-2018 -1.5 ± 0.05 km^2 a^{-1}) when compared to our earlier epoch (1974-2004- -0.6 ± 0.03 km^2 a^{-1}). Considerable glacier surface lowering was also observed throughout the observation period; however, the nature of mass loss was highly heterogeneous from one glacier to another (Supplementary Table 6). The overall (1974-2018) mass loss rate for all glaciers was -0.36 ± 0.07 m w.e.a^{-1}, due to the lowering of glacier surfaces at a mean rate of -0.42 ± 0.11 ma^{-1}. The mass loss rate from 2004-2018 was considerably higher (-0.42 ± 0.11 m w.e.a^{-1}) than from 1974-2004 (-0.30 ± 0.10 m w.e.a^{-1}). The clean ice Kangwure glacier (G085816E28470N) experienced considerable mass loss (-0.63 ± 0.11 m w.e.a^{-1}) from 2004-2018, which increased again from 2018-2019 (-0.77 ± 0.16 m w.e.a^{-1}).

Studies such as Ragettli et al. (2016)[41] (-0.24 ± 0.08 m w.e.a^{-1} from 1974-2006 and -0.45 ± 0.18 m w.e.a^{-1} from 2006-2015) and King et al. (2019)[42] (-0.27 ± 0.10 m w.e.a^{-1} from 1974-2000 and -0.37 ± 0.11 m w.e.a^{-1} from 2000-2015) both noted similar increases in the rate of mass loss over two broad study periods that our results also suggest in the Poiqu basin. Ice mass loss rates (-0.42 ± 0.11 m w.e.a^{-1}) indicated during our latest (2004-2018) study period are in line with those based on the data of Brun et al. (2017)[33] (Supplementary Table 24).

The Langtang sub region also experienced an increased mass loss rate in the recent time period (-0.50 ± 0.11 m w.e.a^{-1} for 2004-2019) when compared to an earlier time period (-0.23 ± 0.10 m w.e.a^{-1} for 1964-2004). The contemporary mass loss rate in the Langtang sub-region (-0.50 ± 0.11 m w.e.a^{-1} for 2004-2019) was greater than in the broader Poiqu region (-0.42 ± 0.11 m w.e.a^{-1} for 2004-2019). (Supplementary Table 7). The debris-covered Langtang (G085816E28470N) and Langshisha (G085747E28200N) glaciers, located in the Langtang valley, also showed increased mass loss rates throughout the time span (-0.27 ± 0.10 m w.e.a^{-1} & -0.37 ± 0.10 m w.e.a^{-1} for 1964-2004, -0.62 ± 0.12 m w.e.a^{-1} & -0.61 ± 0.12 m w.e.a^{-1} from 2004-2019 respectively). The most substantial glacier thinning we measured between 1964–2004 occurred at Shalbachum (−0.45 ± 0.13 m a^{-1}) and Ghanna (−0.48 ± 0.13 m a^{-1}) glaciers. The lowest rate of thinning occurred on Kimoshung (-0.07 ± 0.12 m a^{-1} for 1964-1974) glacier. Considering the periods 1964–2004 and the 2004–2019, the strongest thinning acceleration took place at Langtang Glacier (from −0.32 ± 0.13 m a^{-1} to −0.73 ± 0.15 m a^{-1}).

As with the broader Poiqu region, increased ice loss rates have been noted by other studies over broad study periods in the Langtang catchment. Our more temporally resolved mass loss estimates reaffirm these observations and suggest heightened contemporary mass loss. King et al. (2019)[42] and Ragettli et al. (2016)[41] measured comparable ice loss rates (-0.29 ± 0.10 m w.e.a^{-1} versus -0.21 ± 0.08 m w.e.a^{-1}) between 1974-2000 and 1974-2006, respectively. Our data (-0.24 ± 0.10 m w.e.a^{-1}) agree with these estimates over a similar time period (1974-2004). Ragettli et al. (2016)[41] derived a mean mass balance of -0.38 ± 0.17 m w.e.a^{-1} between 2006-2015. Our estimates of ice loss from 2009-2015 (-0.55 ± 0.12 m w.e.a^{-1}) and 2015-2017 (-0.57 ± 0.11 m w.e.a^{-1}) confirm the accelerating trajectory of ice loss in the Langtang.

3.6. Gurla Mandhata (Naimona'nyi): Glaciers in Gurla Mandhata shrank by 4.3 ± 1.5 km^2 over our 53 years study period (Supplementary Table 15), which represents a loss of 5.9 ± 2.1% of the

total 1966 glacier area (an annual rate of -0.08±0.03 $km^2a^{-1,}$ or -0.11 ± 0.04% a^{-1}). Glaciers thinned at a mean rate of -8.4 ± 6.3 m from 1966 to 2019, which equates to a mean mass balance of -0.13 ± 0.09 m w.e.a^{-1}. Mass loss rates from 1966 to 2000 (-0.12 ± 0.10 m w.e.a^{-1}) and 2000 to 2011 (-0.12 ± 0.11 m w.e.a^{-1}) mirrored the overall mass loss rate for this region, however almost balanced conditions were observed (-0.02 ± 0.07 m w.e.a^{-1}) from 2011 to 2013 (Supplementary Table 8). Mass loss rates increased considerably from 2013 to 2016 (-0.20 ± 0.11 m w.e.a^{-1}) and from 2016-2018 (-0.22 ± 0.11 m w.e.a^{-1}). The maximum mass loss (-0.29 ± 0.09 m w.e.a^{-1}) was observed for the glacier no. G081365E30442N from 1966-2019. NanNamuNaNi (G081307E30424N) is the only glacier in the study area that is largely covered by debris and exhibited higher mass loss than regional average (-0.22 ± 0.09 m w.e.a^{-1} from 1966-2019). Similar mass loss was also observed for glaciers G081317E30454N (Gurla) (-0.24 ± 0.09 m w.e.a^{-1}), G081379E30472N & G081349E30428N (both -0.23 ± 0.09 m w.e.a^{-1}) and G081387E30495N (-0.20 ± 0.09 m w.e.a^{-1}) over the full study period. The remaining six (of 12) investigated glaciers were almost stable, with mass balance estimates varying from -0.10 ± 0.09 to -0.03 ± 0.09 m w.e. a^{-1} from 1966 to 2019 (Supplementary Table 8).

Geodetic studies have captured almost balanced glacier mass budgets or slight glacier mass loss since 2000 in Gurla Mandhata, but the recent increases in mass loss shown by our data have not been previously noted. Holzer et al. (2014)[43] measured a mean glacier mass balance of -0.07 ± 0.11 m w.e.a^{-1} from 2000-2013 and the data of Brun et al. (2017)[33] suggests a mass loss rate of -0.14 ± 0.07 m w.e.a^{-1} between 2000-2016. Our results indicate moderate mass loss over a slightly shorter time period since 2000 (-0.12 ± 0.10 m w.e.a^{-1} from 2000-2011), which has increased since (Supplementary Table 26).

3.7. Muztagh Ata Massif: Small variations in the rate of frontal recession and area change were evident for glaciers in Muztagh Ata (Supplementary Table 16), but most of the glaciers were stable throughout the observation period (Supplementary Table 9). The mean mass budget of glaciers in Muztagh Ata Massif was slightly negative or nearly balanced (-0.06 ± 0.07 m w.e.a^{-1}) during the entire observation period. However, negative mass balance (-0.14 ± 0.10 m w.e.a^{-1}) was evident from 1967-1973, which was followed by a balanced budget until 2009 (-0.01 ± 0.07 m w.e.a^{-1} for 1973-2009). After 2009 a moderate signal of glacier mass loss (-0.10 ± 0.12 from 2009-2013, -0.12 ± 0.09 m w.e.a^{-1} from 2013-2019) was evident (Supplementary Table 9).

Overall glacier area reduction was -3.8 ± 1.0 km^2, from 347.6 ± 9.2 km^2 in 1967 to 343.7 ± 4.1 km^2 in 2019. The largest glacier (Kekesayi Glacier-G075225E38255N- 84.0 ± 1.7 km^2 in 1967) in the region, lost little ice cover over the full study period (-0.03 ± 0.8 km^2) and its surface elevation reduced by -0.2 ± 0.1 ma^{-1} from 1967-2019.

Surge behavior was evident in several glaciers of Muztagh Ata Massif over various portions of the observation period. A clear and rapid advance of the terminus of Kuosikulake (G075092E38214N) and KukKuosele (G075156E38175N) glaciers was evident, starting in 2009 and 1973 respectively. Kuokuosele glacier (G075156E38175N) experienced a strong elevation gain (3.78 ± 3.89 ma^{-1}) at its tongue from 1973 to 2019. Glacier G075075E38189N also experienced similar elevation gain (0.56 ± 1.10 $ma^{-1)}$ during its advance (2001-2013), however, its tongue began to thin from 2013-2019 (0.28 ± 0.89 ma^{-1}). Despite their considerable debris cover, Kekesayi (G075225E38255N) and G075171E38163N showed considerable down-wasting (Supplementary Table 9).

Generally stable long-term glacier mass budgets have been noted by other studies around the Mustag Ata massif, but, again, the temporal resolution of our time series captures the subtle variations in mass balance contained within longer study periods of previous work. Holzer et al. (2015)[32] estimated mean glacier mass balance of -0.04 ± 0.42 m $w.e.a^{-1}$ and 0.04 ± 0.27 m $w.e.a^{-1}$ from 1973-1999 and 1999-2013, respectively. Zhang et al. (2016)[44] suggest moderate mass loss rates over two extended periods (-0.15 ± 0.18 m $w.e.a^{-1}$ from 1973-1999 and -0.13 ± 0.23 m $w.e.a^{-1}$ from 1999-2014) in comparison. Our results suggest the transition to mass loss from glacier mass balance occurred since 2009 (Supplementary Table 27).

Supplementary Note 4

4. Elevation change data gap filling methodology

A variety of methods have been proposed to fill glacier surface elevation change data to be used in the derivation of geodetic glacier mass balance estimates. McNabb et al. (2019)[46] examined the impact of eleven different void filling techniques on the derived mass balance of a sample of glaciers in southeast Alaska. McNabb et al. (2019)[46] illustrate the strengths and weaknesses of each method in their test case and highlight which approaches reproduce regional total glacier volume change to within 1% of a true estimate. Of the eleven methods examined by McNabb et al. (2019)[46] we employ the regional median hypsometric approach, which is slightly different to the local mean hypsometric method, which was ranked as most accurate in the study of McNabb et al. (2019)[46].

To illustrate the impact of the two different approaches we assessed the sensitivity of elevation change values to the implementation of a mean hypsometric filling approach compared to median hypsometric filling, in two different cases (Supplementary Figure 36 and Supplementary Table 29). In agreement with McNabb et al. (2019)[46], it is evident that the void filling methods do not substantially affect values of elevation change if the elevation change dataset is normally distributed (one example of the elevation change distribution of Ak-Shirak region for the year 1964-2019 is shown below). The second histogram, the elevation changes distribution of Northern Tien Shan region for the year 1964-2020, shows a skewed distribution of elevation change. For this non-normally distributed data, different gap filling methods result in a slight but insignificant deviation in mass budget (-29.59±6.7 m or -0.45±0.09 m.w.e.a^{-1} using regional mean hypsometry and -26.12±6.7 m or -0.40±0.09 m.w.e.a^{-1} using regional median hypsometry). Subsequently, in our study area we have considered the regional median gap filling method to be more accurate as the median is more robust and less sensitive to any remaining outliers in the DEM difference images after outlier removal than the mean. For example, the median does not change substantially when we consider all elevation changes outside ±100 m or ±150 m as outliers, but the change in mean is more pronounced (Supplementary Table 30). As we cannot be certain about the value of the obvious outlier, we have considered median to be more representative of the central tendency of the data.

We have applied median hypsometric void filling at the regional scale over the majority of our study areas. However, in some study areas, such as Ak-Shirak, Purogangri and Muztagh-Ata

Massif, where surge type glaciers are present, we have applied median hypsometric void filling separately for surge-type and non-surge type glaciers. This approach was required because the mass displacement involved in glacier surging is of a much greater magnitude than climate induced glacier thinning (or thickening) over the same time scale, thus surge-type glacier elevation changes would not be representative of the wider glacier population in the case of regional hypsometric void filling.

Where surge-type glaciers are not prevalent, we examined the impact of applying median gap filling on a regional or glacier by glacier basis (local). We have computed the resulting mass balance of glaciers in the Purogangri Ice Cap (PIC) region over the period 2000-2012 using both regional and glacier by glacier (local) median hypsometry gap filling. We chose this location and time period because of the near-balanced glacier mass budget, which would be more sensitive to changes in our methodological approach. Gap filling using a regional median hypsometry yielded a near balanced mass budget for this time period (-0.03 ± 0.02 m w.e.a^{-1}, -0.31 ± 0.3 ma^{-1} elevation change). Median hypsometric gap filling on a glacier-by-glacier basis (local) yielded almost identical results (-0.02 ± 0.02 m w.e.a^{-1} or -0.26 ± 0.3 ma^{-1} elevation change). This exercise confirms that our results are robust and the recalculation of all the data for all the periods and regions will not change the finding of our study.

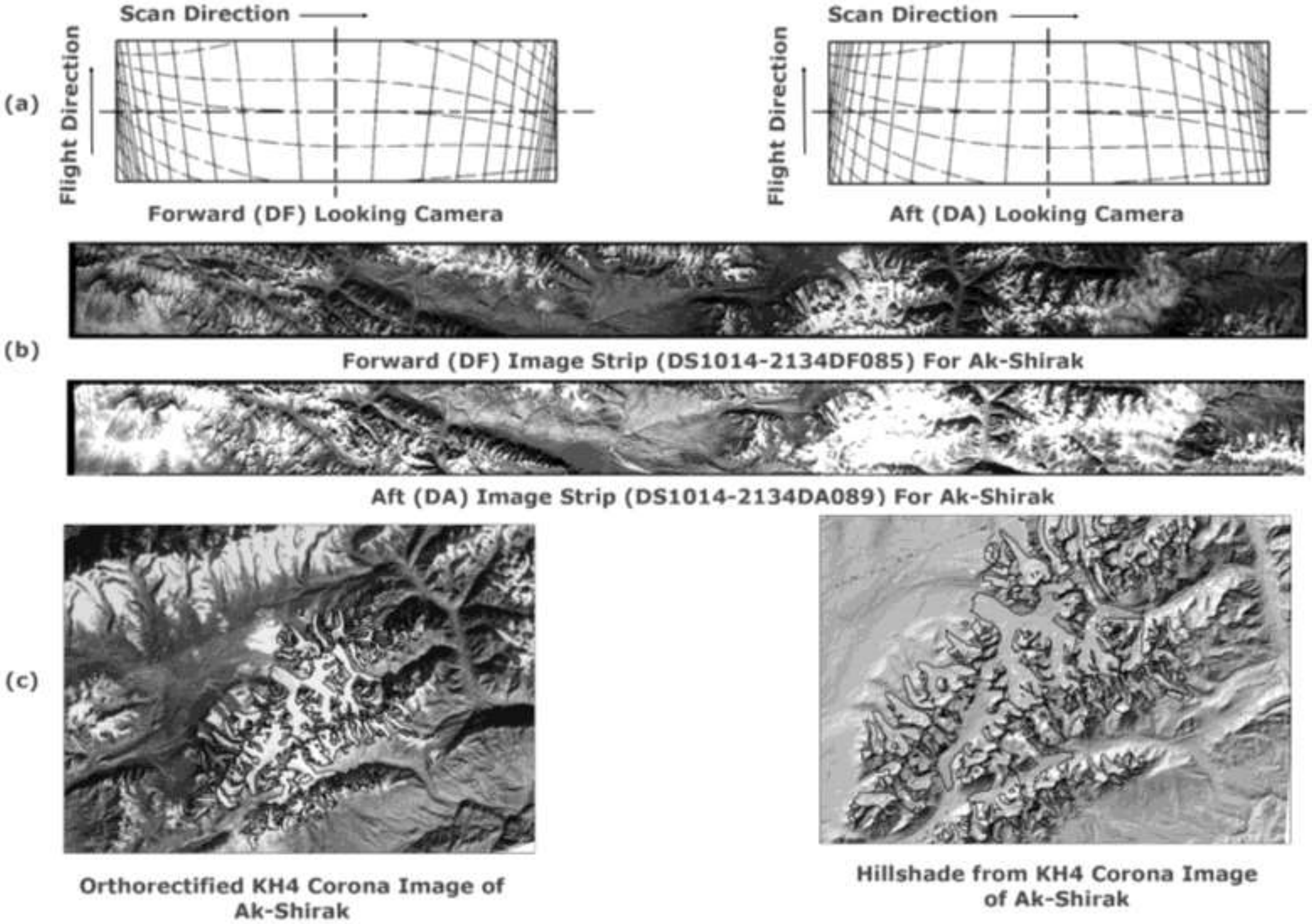

Supplementary Figure 1 (a) Distortion pattern of Corona KH-4 images, (b) Forward and Aft raw images and (c) Orthorectified image and hillshade of the Corona DEM of the Ak Shirak region.

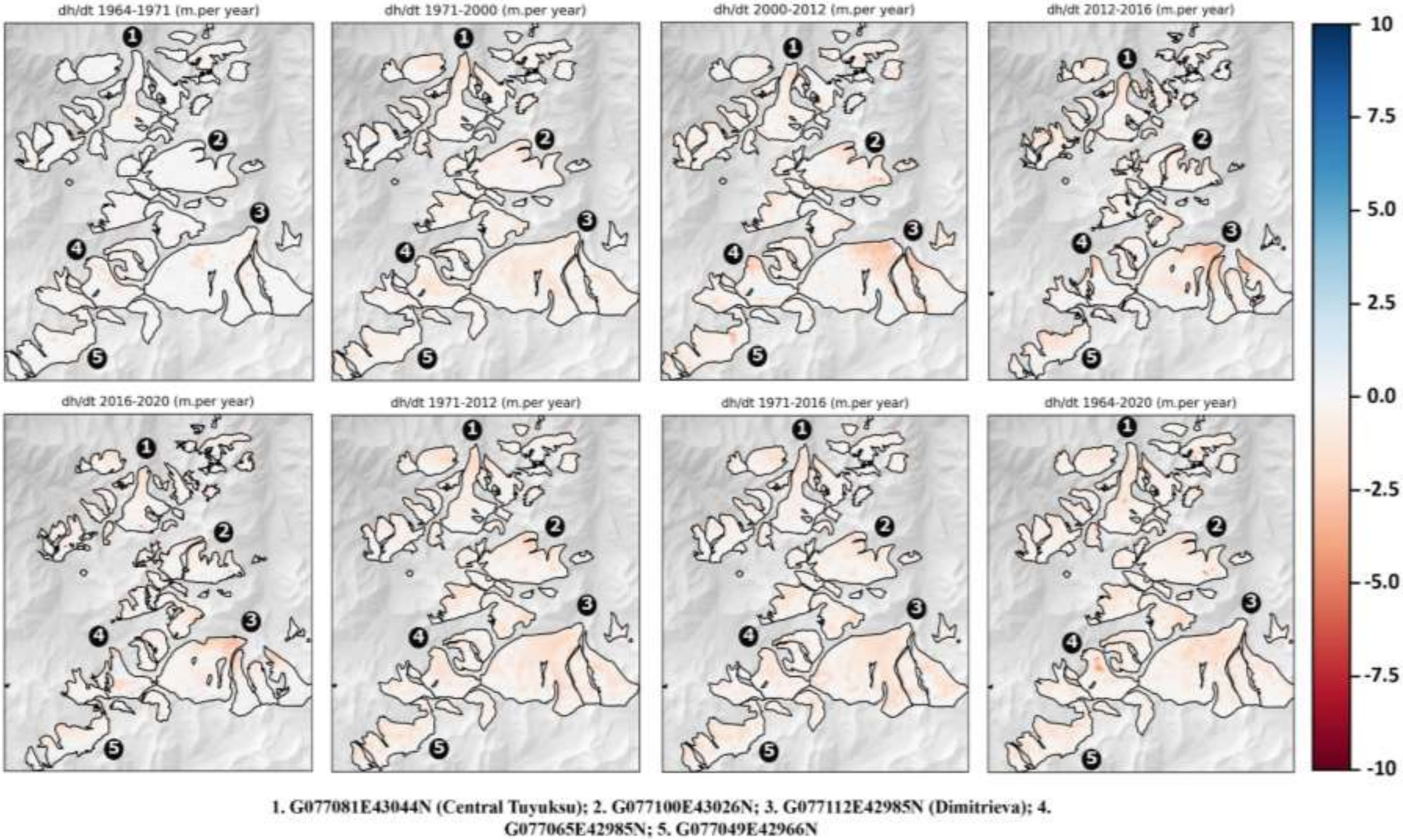

Supplementary Figure 2 Glacier surface elevation differences of the available time periods for the Northern Tian Shan region. The elevation difference during 1964-2016 of glacier 1 (Tuyuksu Glacier, G077081E43044N) is also shown in Fig. 5 (a).

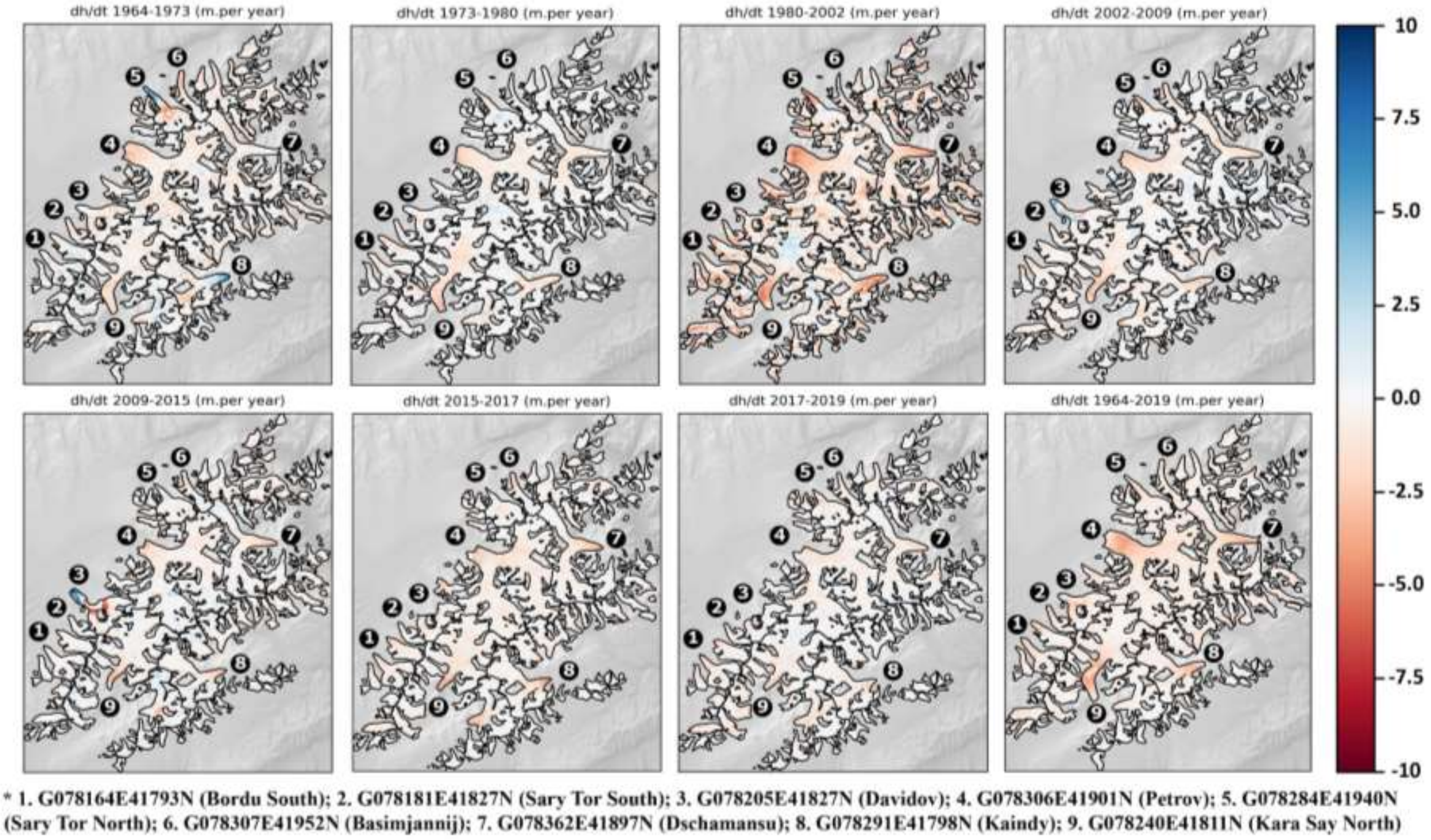

Supplementary Figure 3 Glacier surface elevation differences of the available time periods for the Ak-Shirak region.

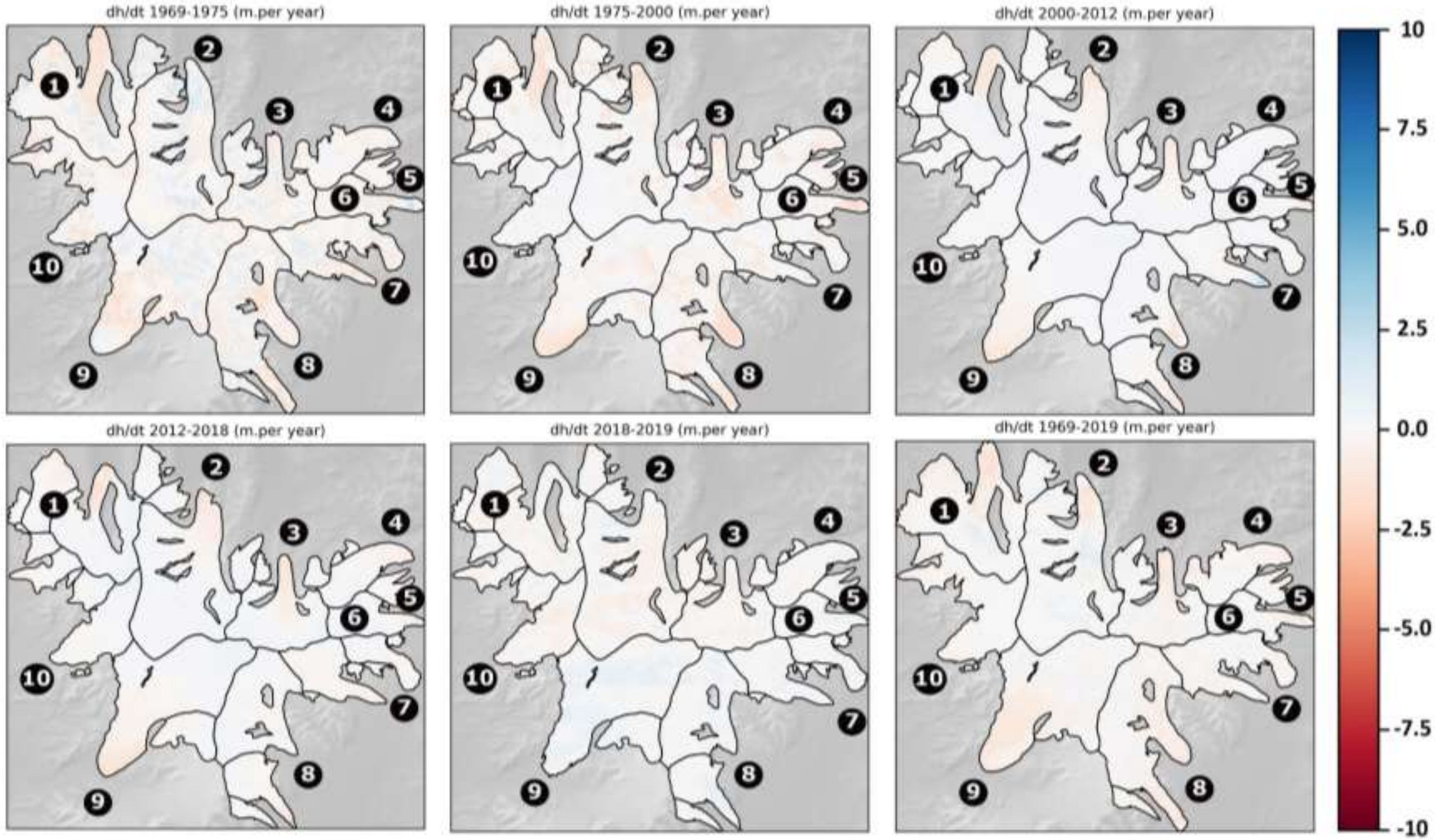

* 1. G089071E33998N; 2. G089128E33943N; 3. G089181E33971N; 4. G089231E33964N; 5. G089281E33945N; 6. G089272E33936N; 7. G089227E33891N; 8. G089189E33837N; 9. G089122E33894N; 10. G089034E33984N

Supplementary Figure 4 Glacier surface elevation differences of the available time periods for the Purogangri Ice Cap (PIC) region.

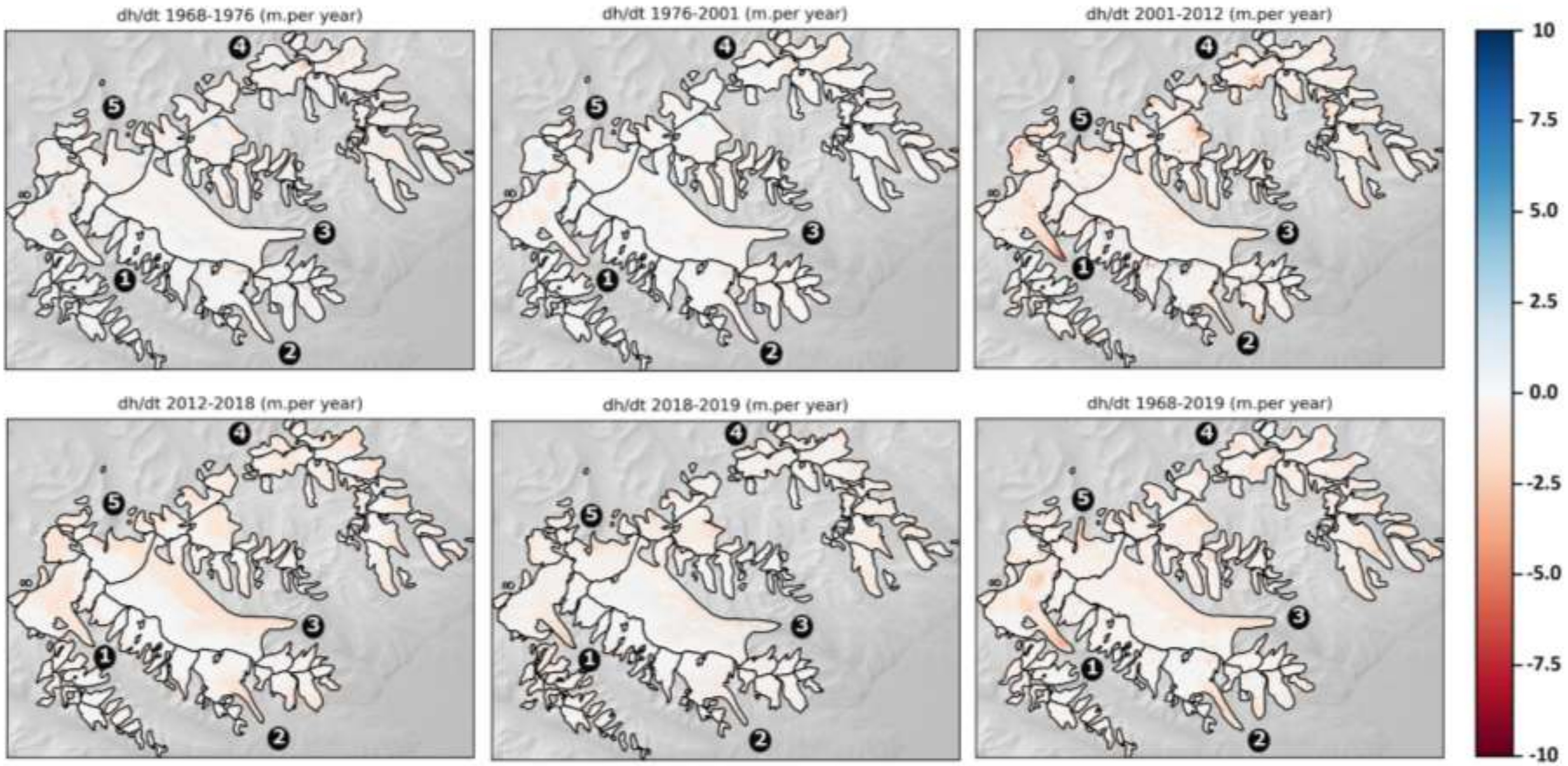

1. G090521E30390N; 2. G090618E30355N; 3. G090600E30388N (Xibu) ; 4. G090639E30472N (Zhadang); 5. G090550E30416N

Supplementary Figure 5 Glacier surface elevation differences of the available time periods for the Western Nyainqentanglha region. The elevation difference during 1968-2019 of glacier 1 (G090521E30390N) is also shown in Fig. 5 (b).

625

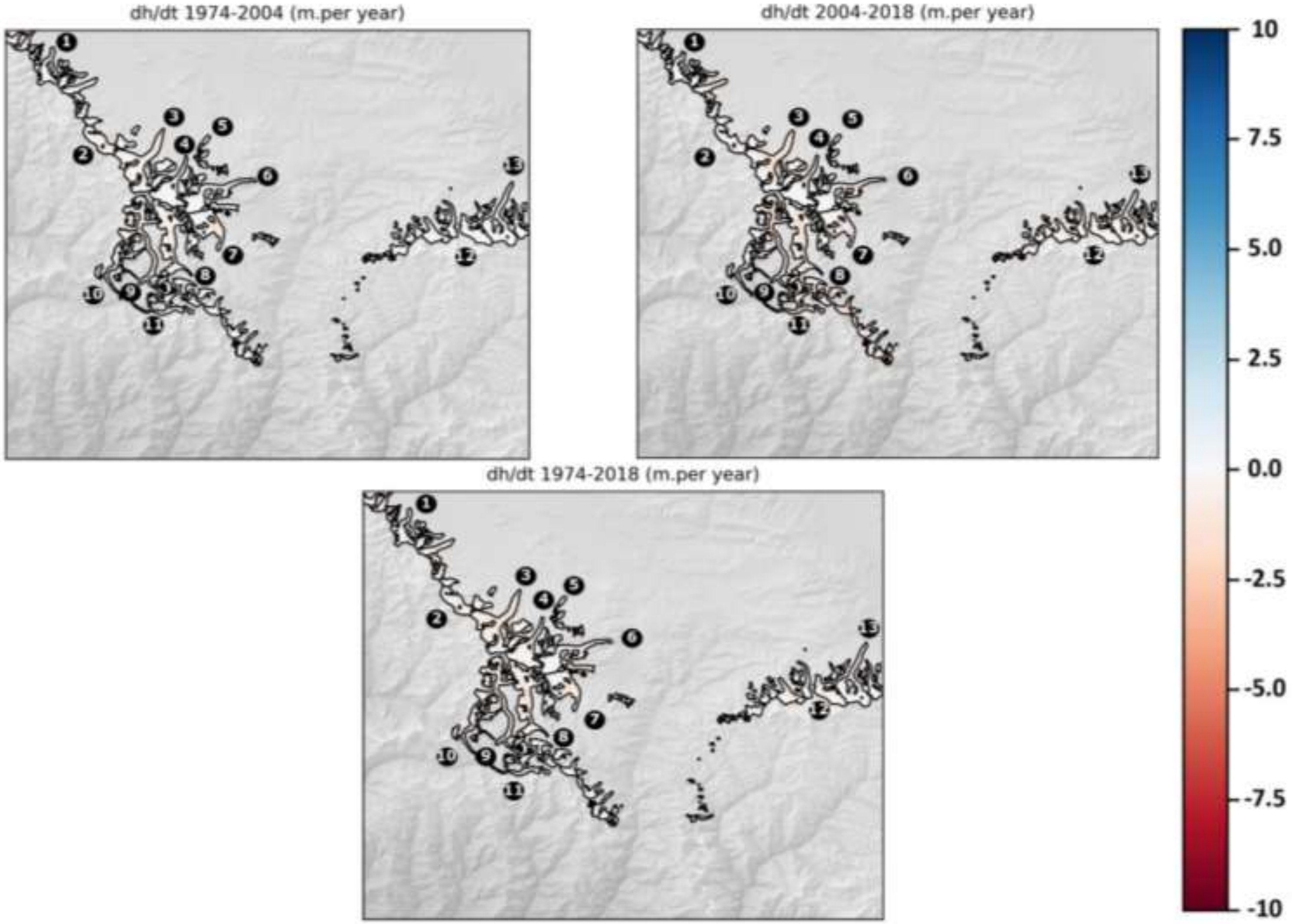

* 1. G085521E28579N; 2. G085618E28467N; 3. G085694E28431N; 4. G085751E28386N; 5. G085816E28470N (Kangwure); 6. G085801E28372N; 7. G085812E28335N; 8. G085751E28316N; 9. G085670E28312N (Langtang); 10. G085645E28262N (Shalbachum); 11. G085747E28200N (Langshisha); 12. G086301E28329N; 13. G086358E28329N

Supplementary Figure 6 Glacier surface elevation differences of the available time periods for the Poiqu region.

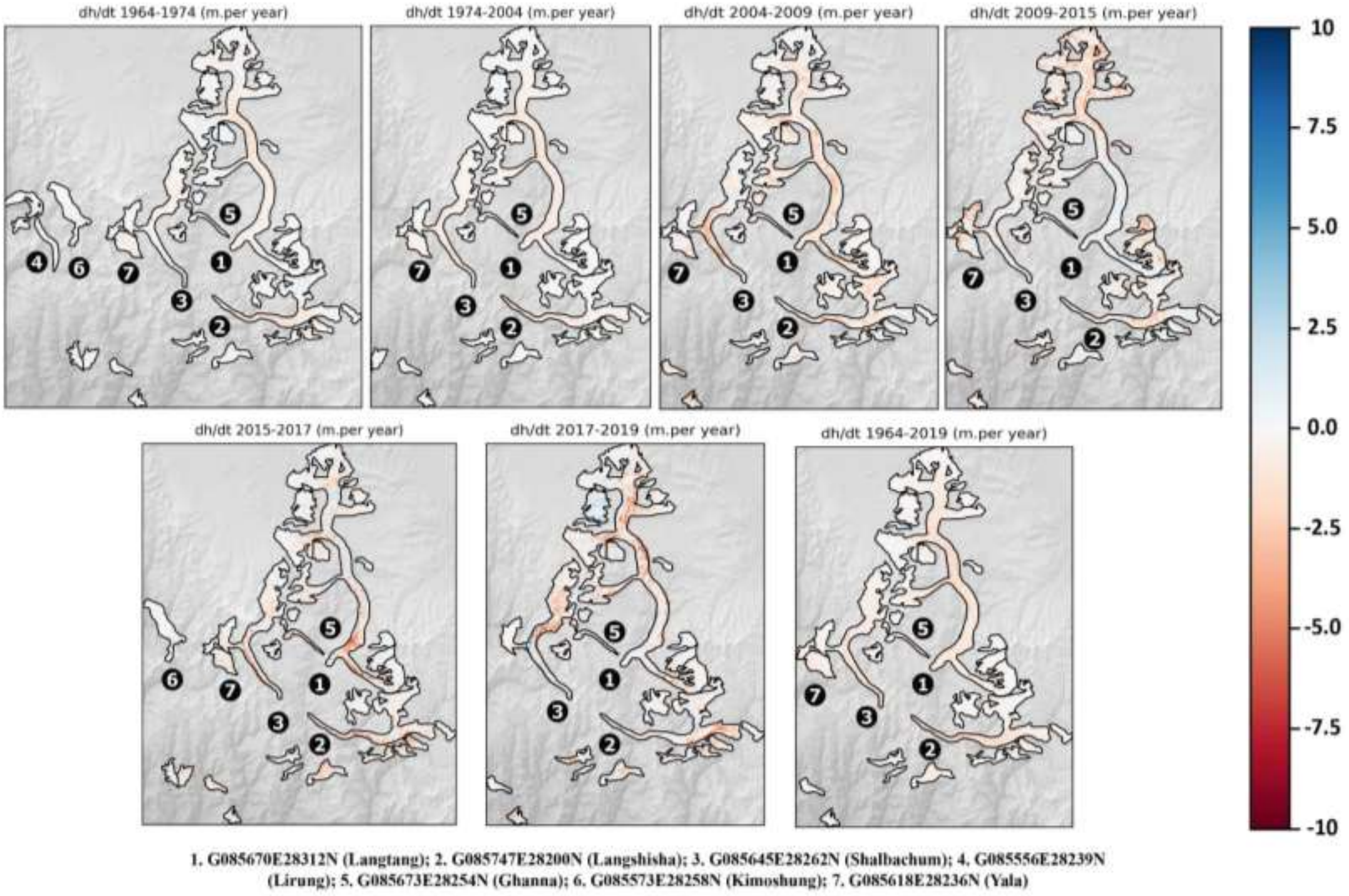

Supplementary Figure 7 Glacier surface elevation differences of the available time periods for the Langtang region.

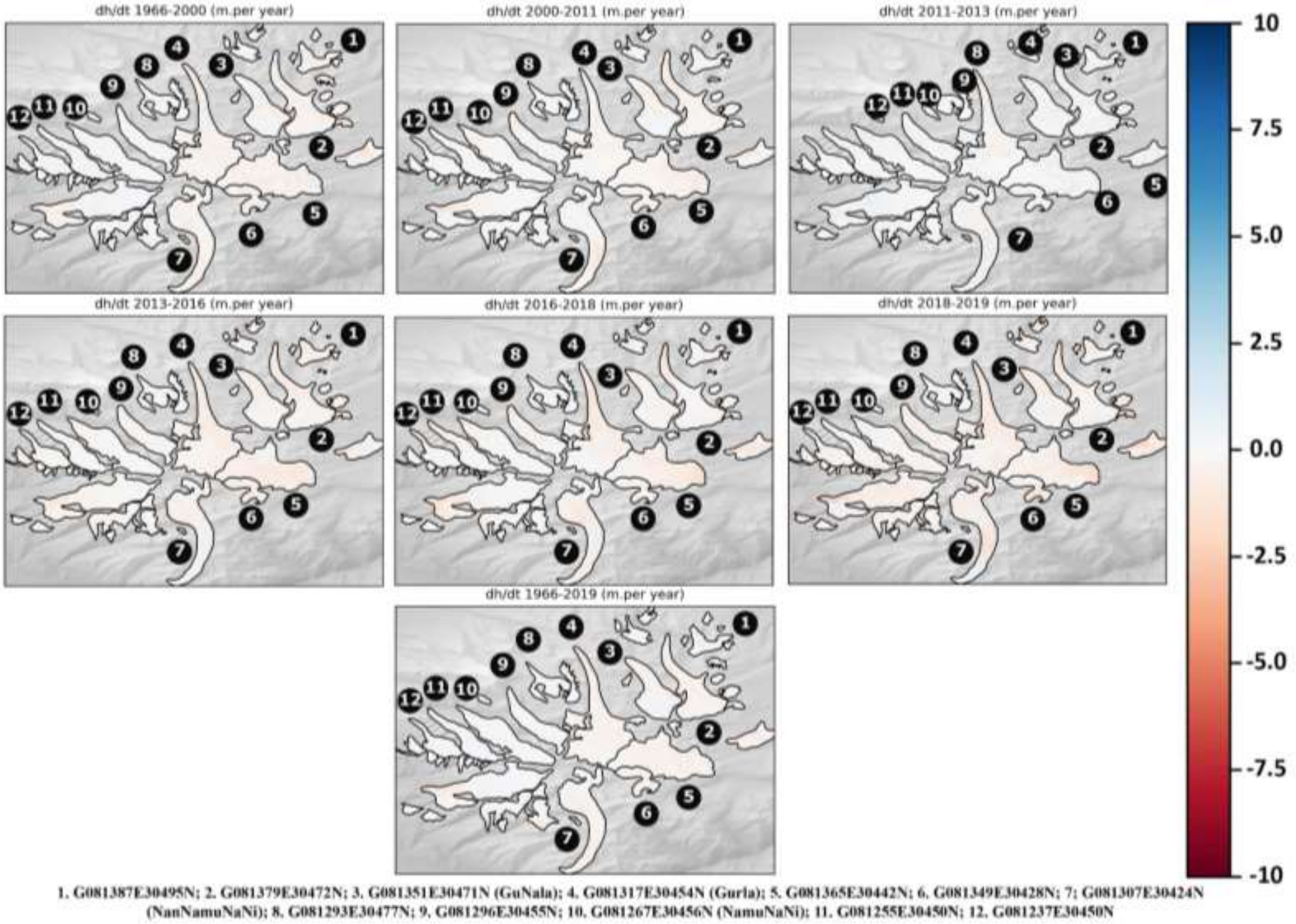

Supplementary Figure 8 Glacier surface elevation differences of the available time periods for the Gurla Mandhata region.

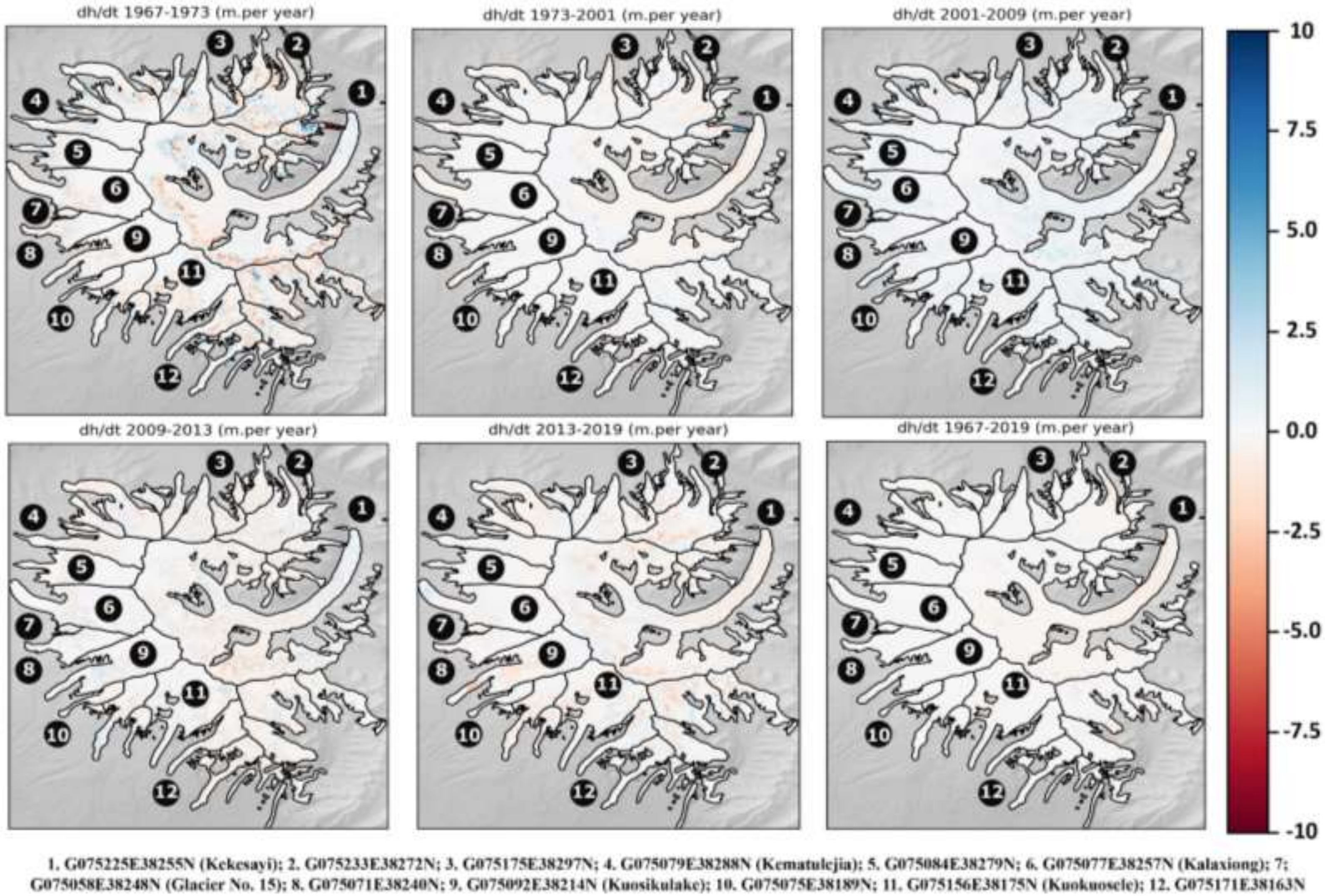

670

Supplementary Figure 9 Glacier surface elevation differences of the available time periods for the Muztagh Ata Massif region. The elevation difference during 1967-2019 of glacier 9 (Kuosikulake Glacier, G075092E38214N) is also shown in Fig. 5 (c).

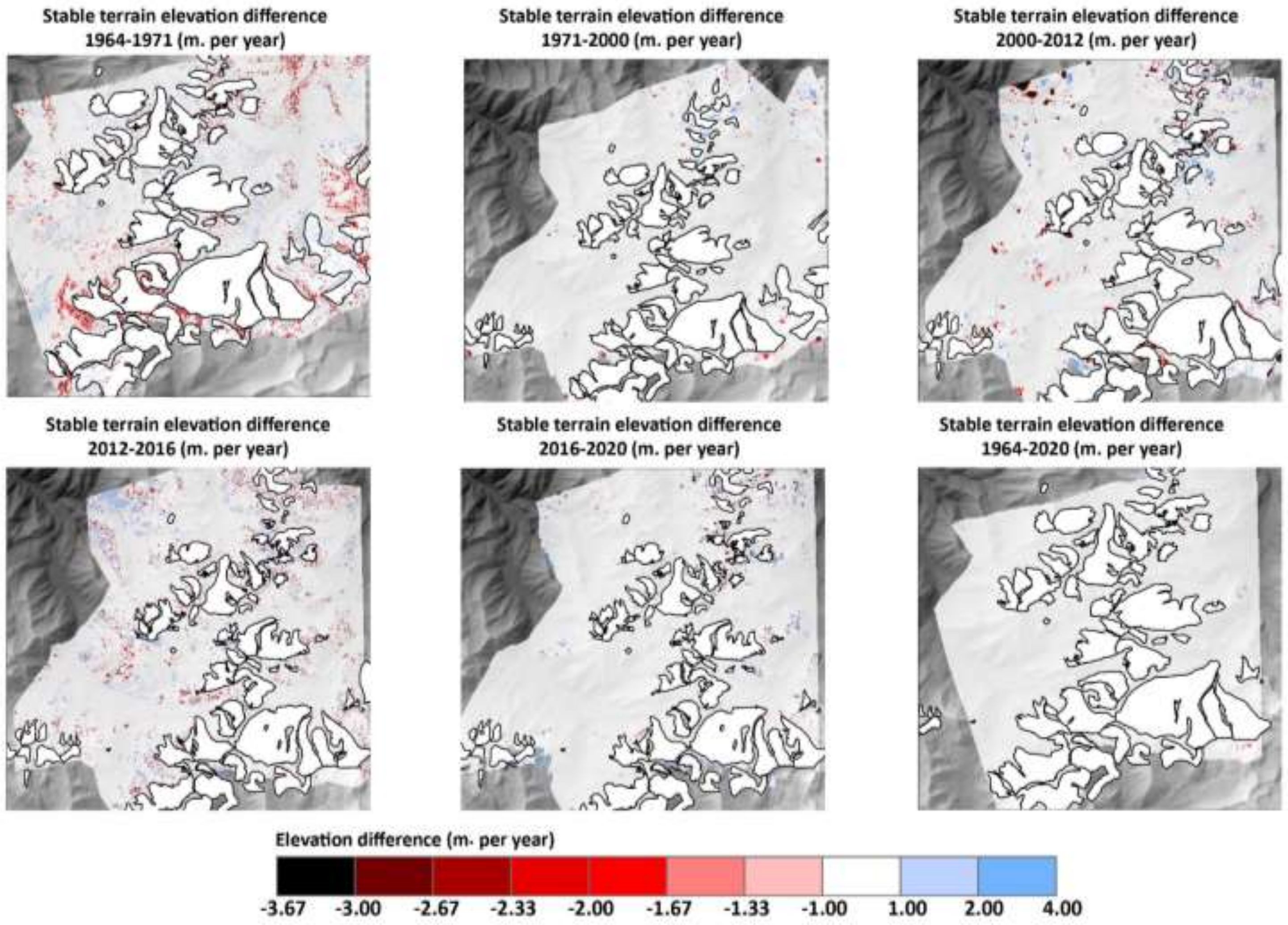

Supplementary Figure 10 Off glacier surface elevation differences of the available time periods for the Northern Tien Shan region.

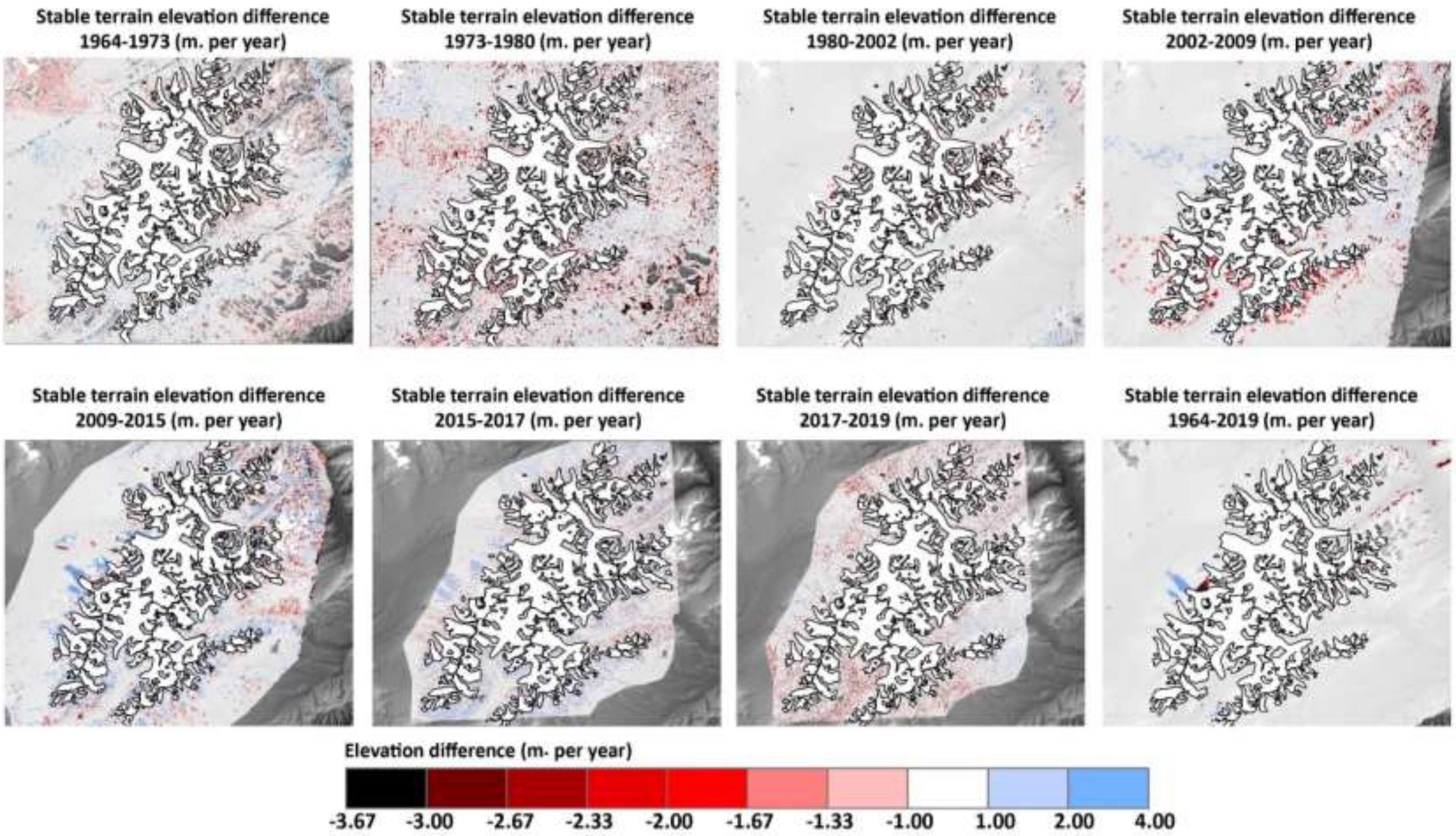

Supplementary Figure 11 Off glacier surface elevation differences of the available time periods for the Ak-Shirak region.

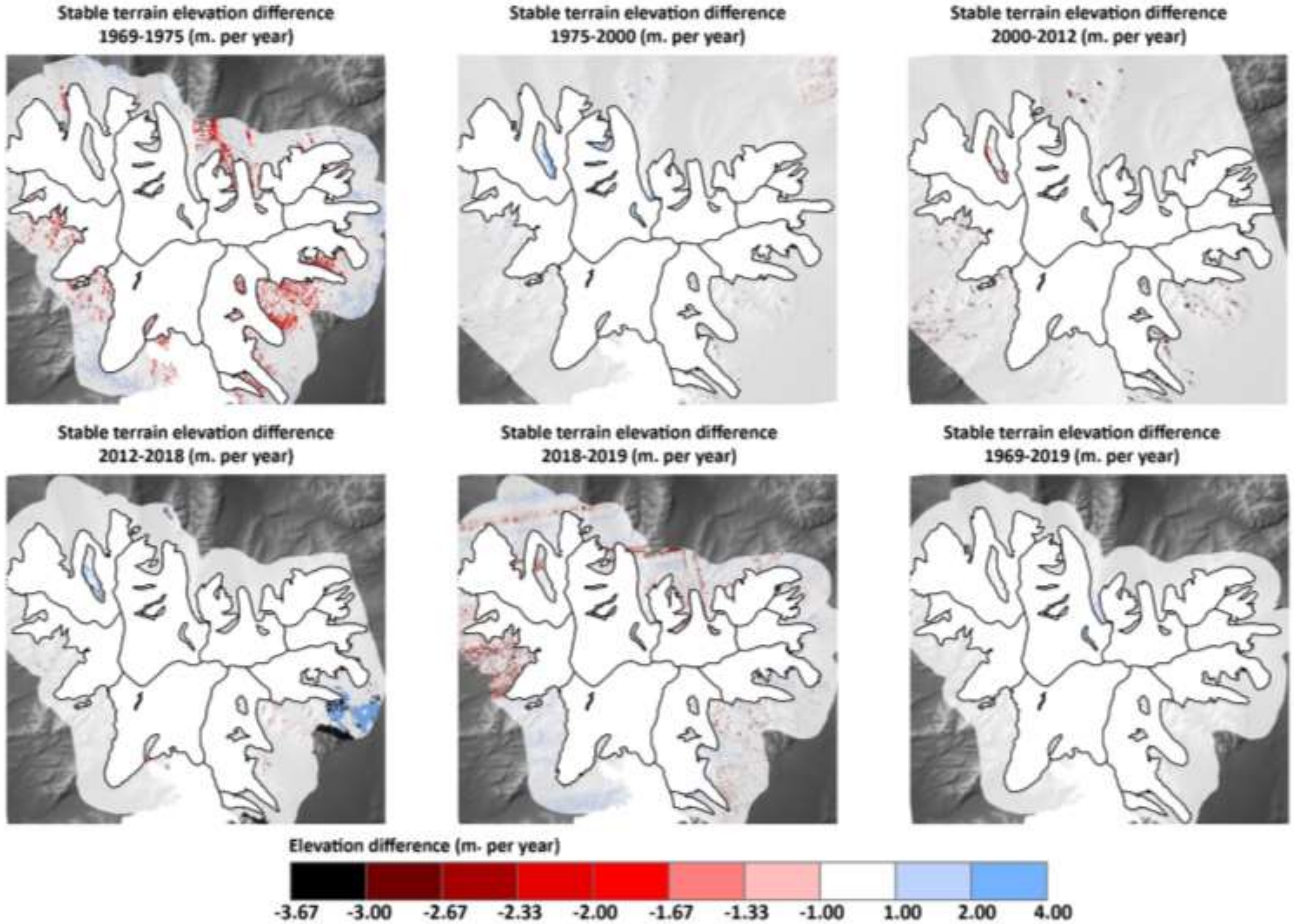

Supplementary Figure 12 Off glacier surface elevation differences of the available time periods for the Purogangri Ice Cap (PIC) region.

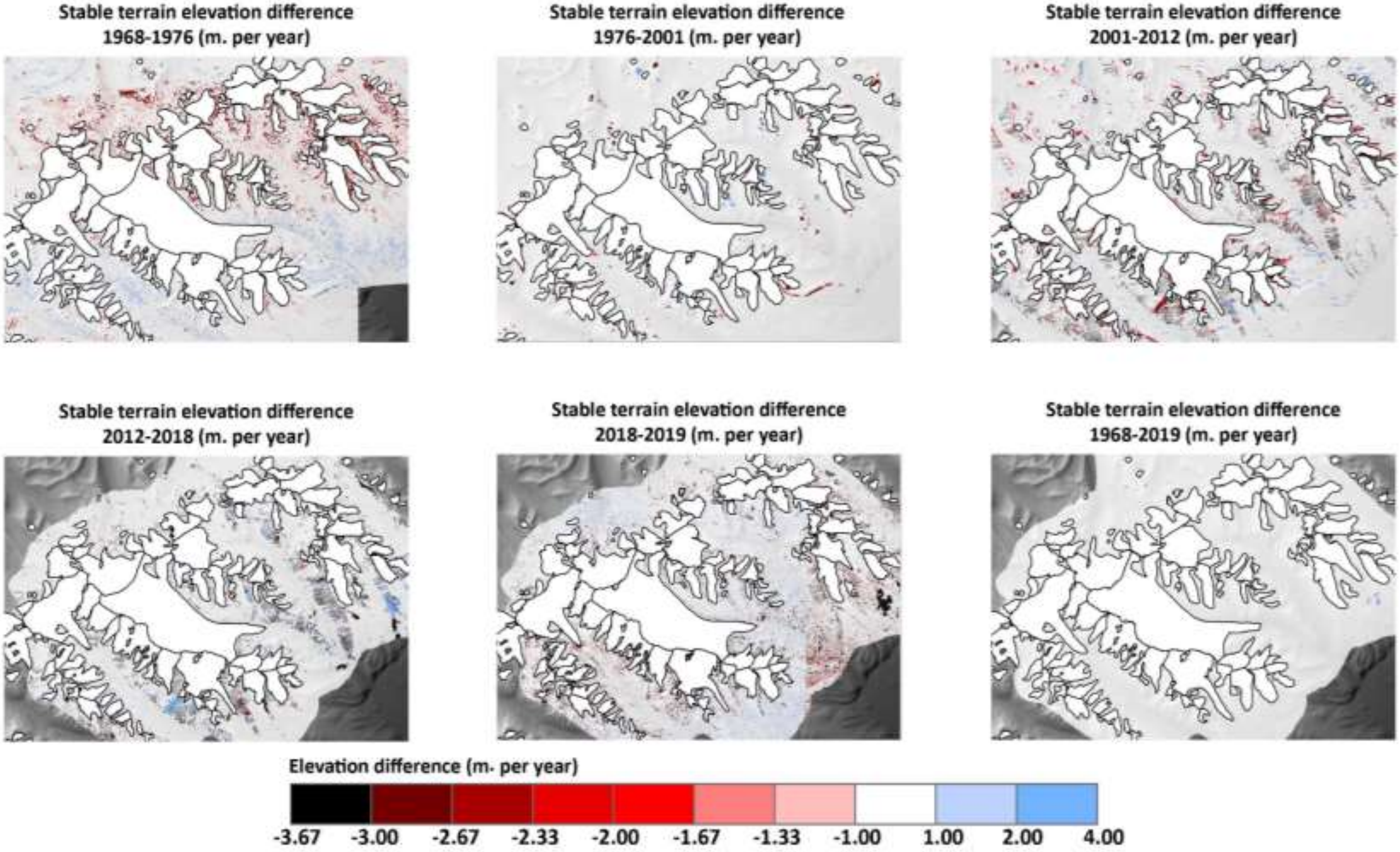

Supplementary Figure 13 Off glacier surface elevation differences of the available time periods for the Western Nyainqentanglha region.

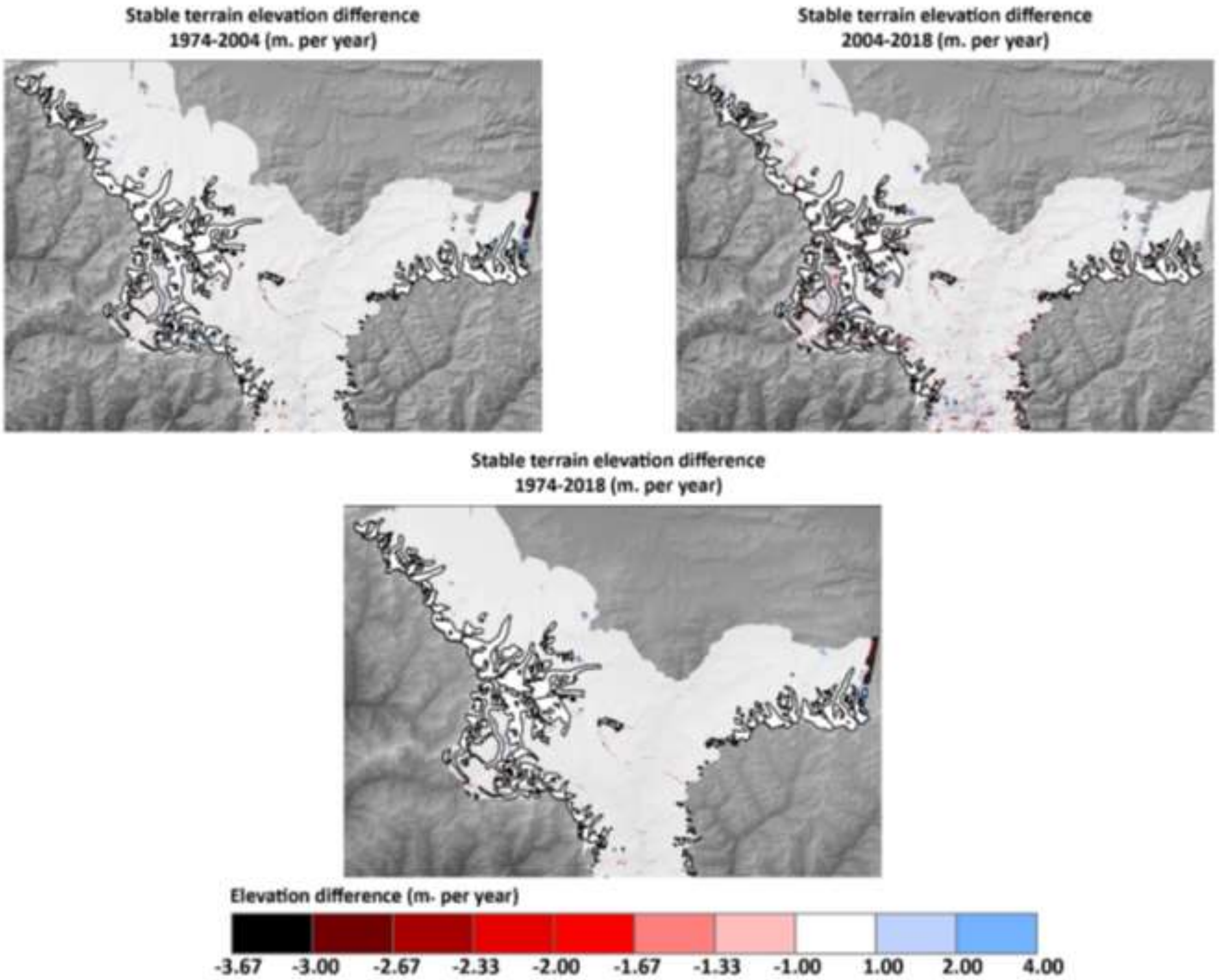

Supplementary Figure 14 Off glacier surface elevation differences of the available time periods for the Poiqu region.

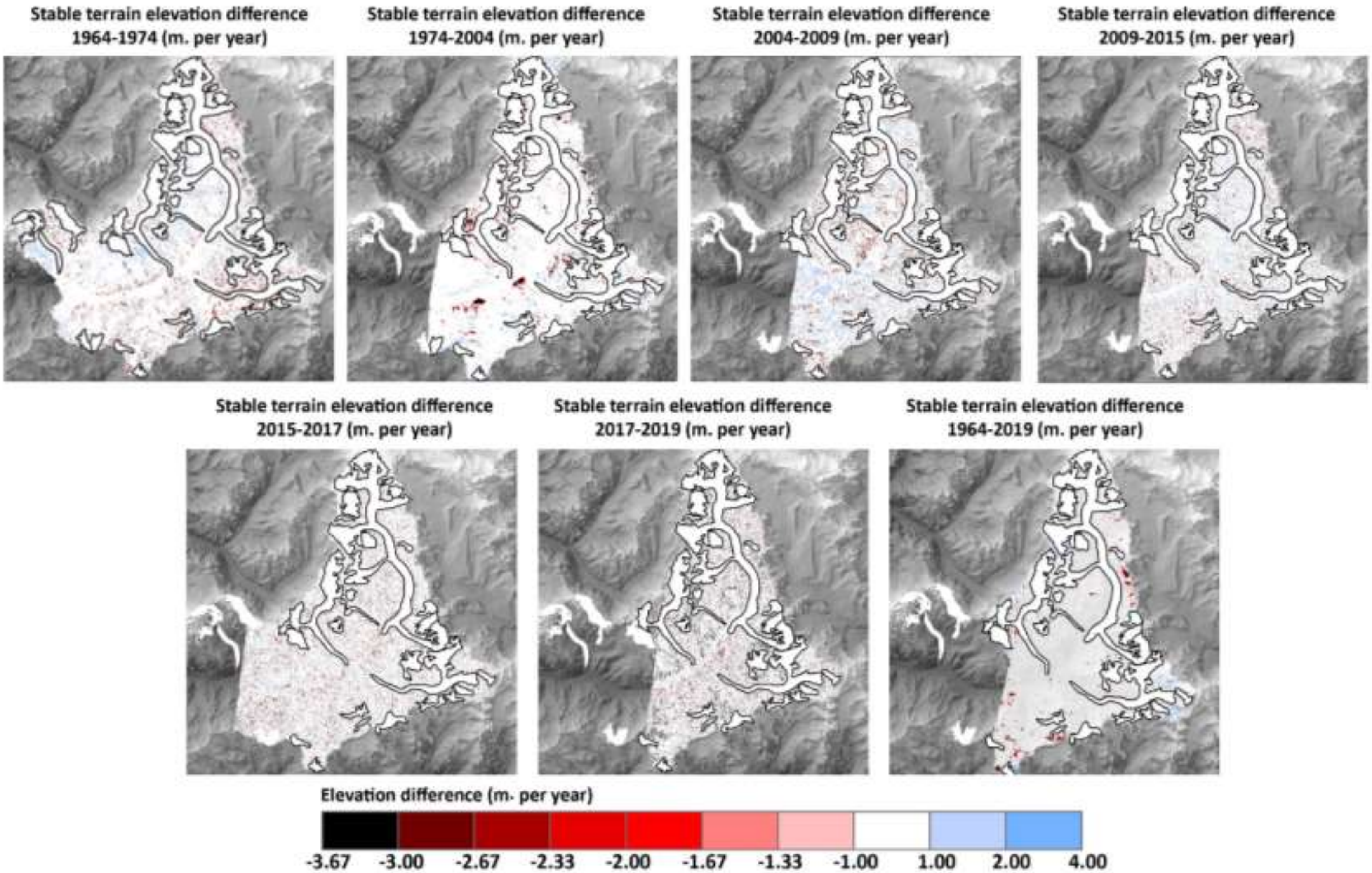

Supplementary Figure 15 Off glacier surface elevation differences of the available time periods for the Langtang region.

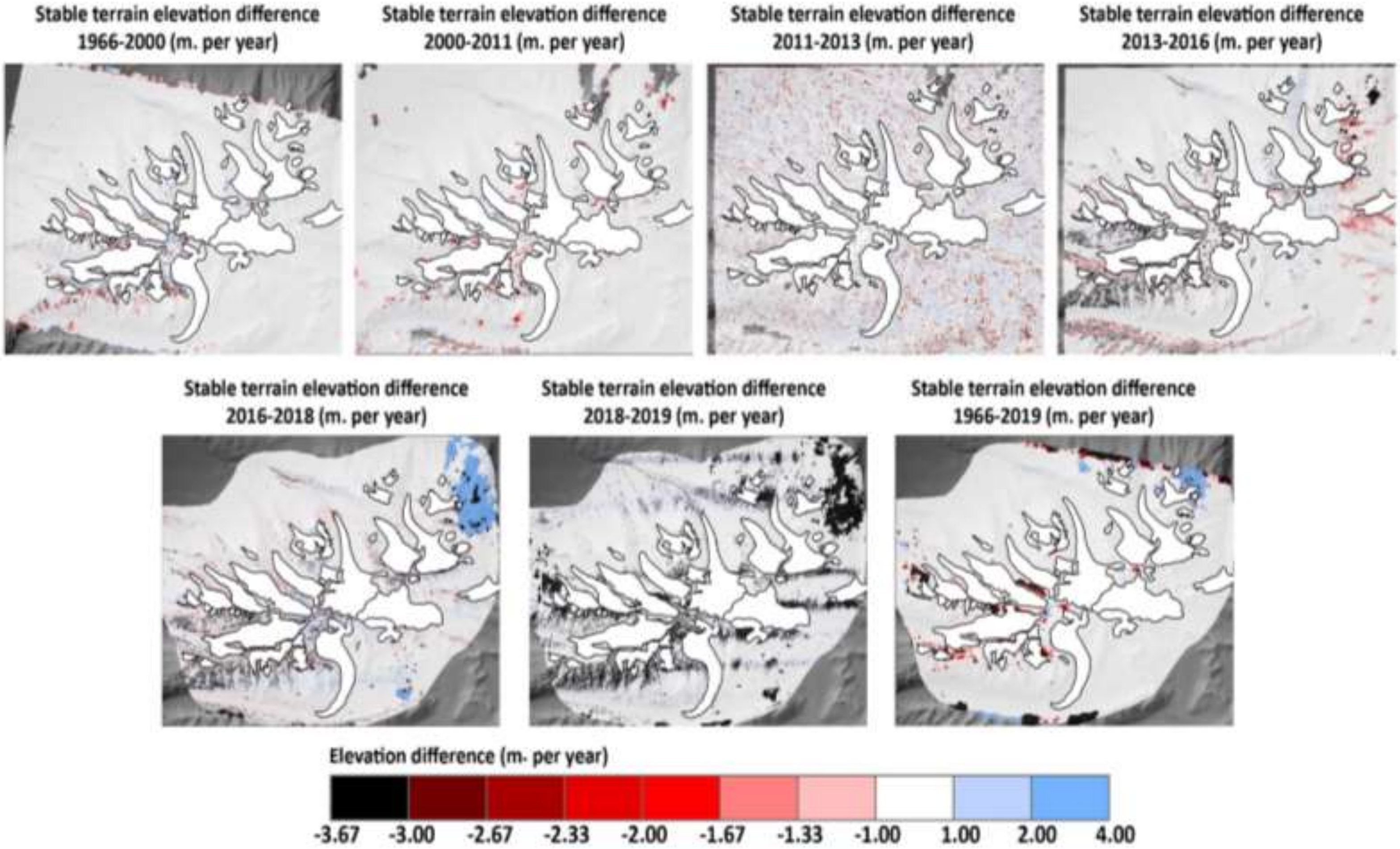

Supplementary Figure 16 Off glacier surface elevation differences of the available time periods for the Gurla Mandhata region.

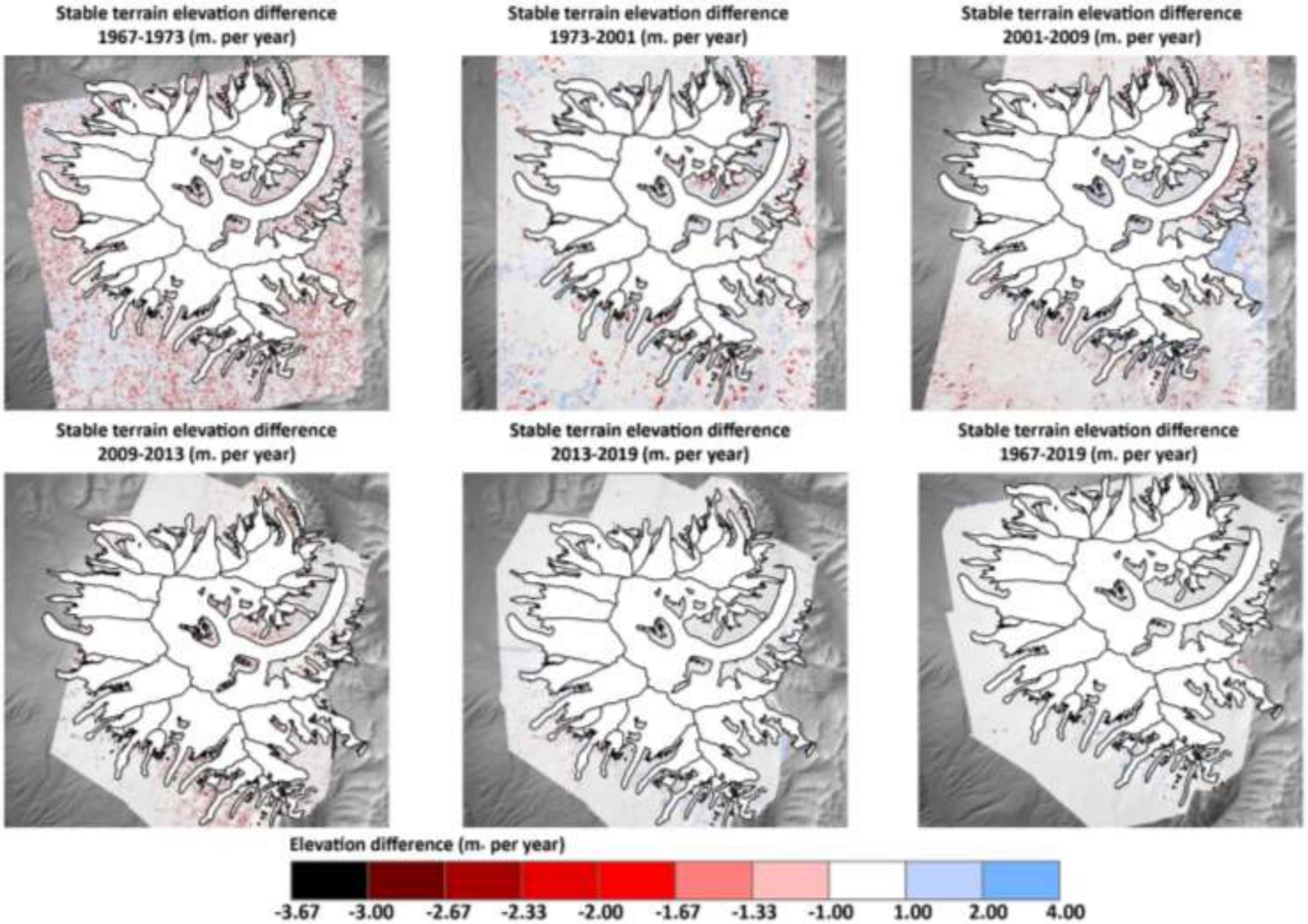

Supplementary Figure 17 Off glacier surface elevation differences of the available time periods for the Muztagh Ata region.

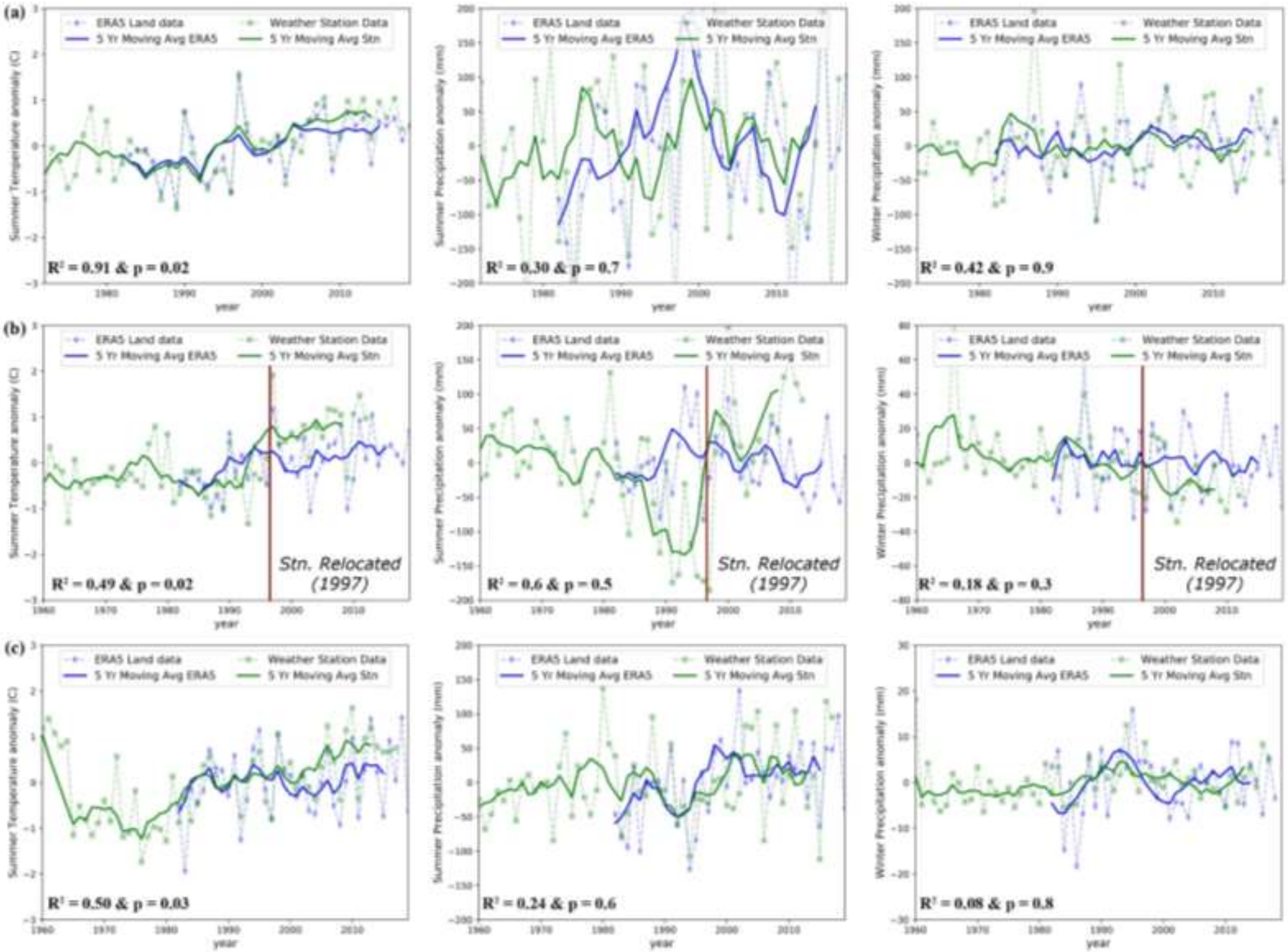

Supplementary Figure 18 Comparison of ERA5 Land data with the data of (a) Tuyuksu (for Northern Tien Shan), (b) Tien Shan (for Ak Shirak) & (c) Bange (for Purogangri Ice Cap) weather stations.

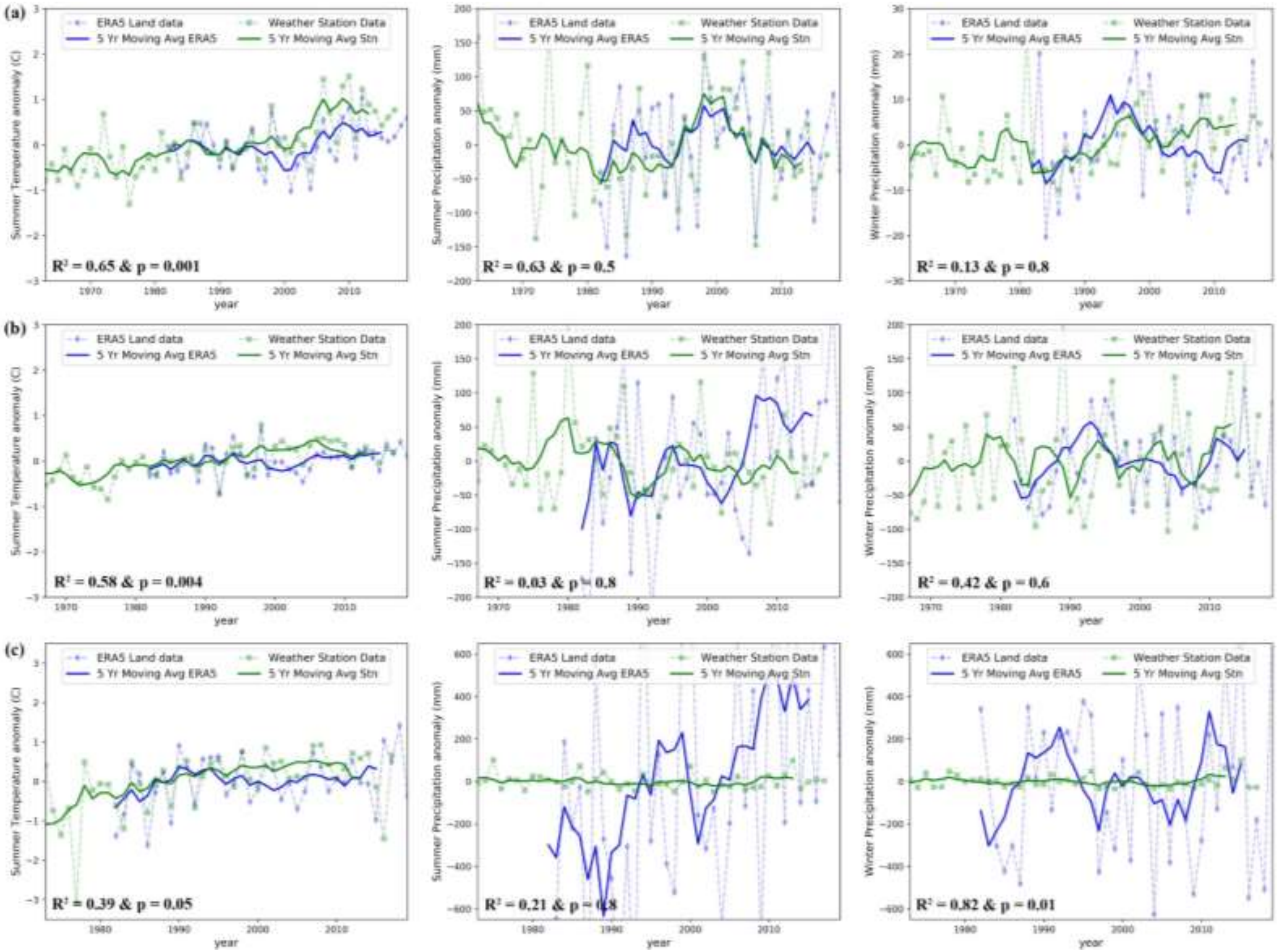

Supplementary Figure 19 Comparison of ERA5 Land data with the data of (A) Dangxiong (for Western Nyainqentanglha), (B) Nielamu (for Poiqu region) & (C) Pulan (for Gurla Mandhata) weather stations.

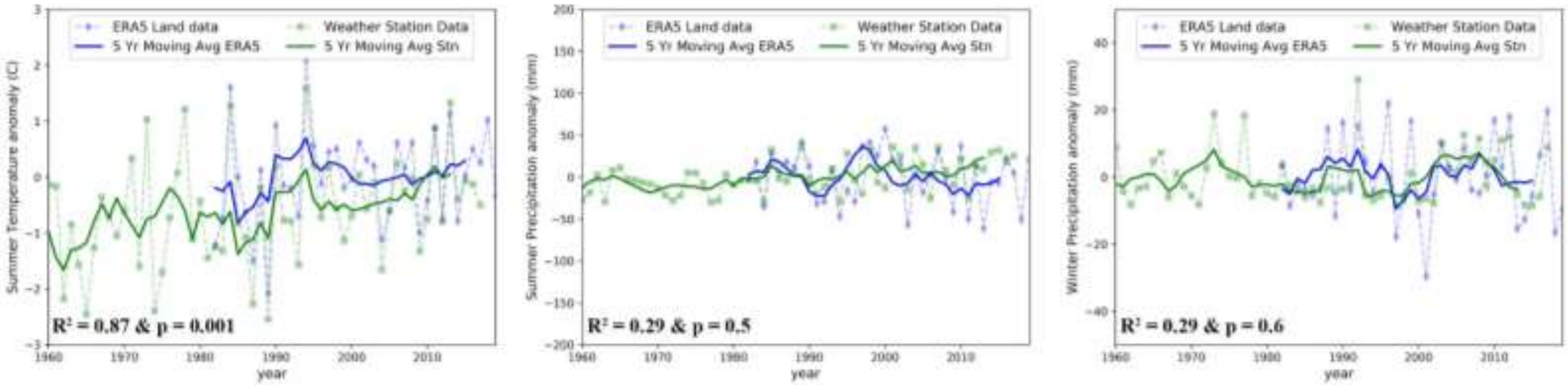

Supplementary Figure 20 Comparison of ERA5 Land data with the data of Tashikuergen (for Muztagh Ata Massif) weather stations.

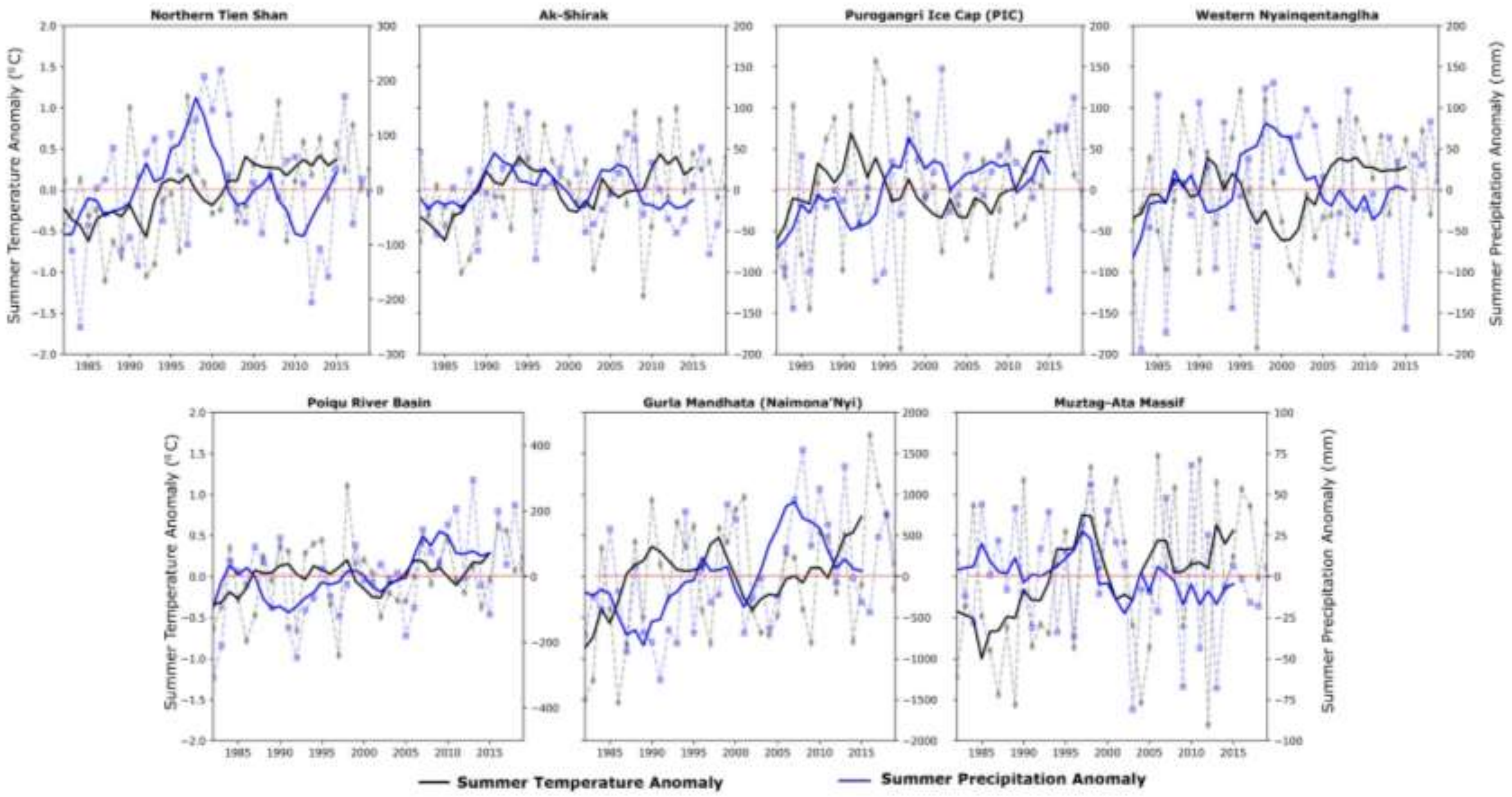

Supplementary Figure 21 Summer temperature and summer precipitation anomaly of ERA 5 Land gridded climate data for all regions. The thick lines represent the 5 years moving average.

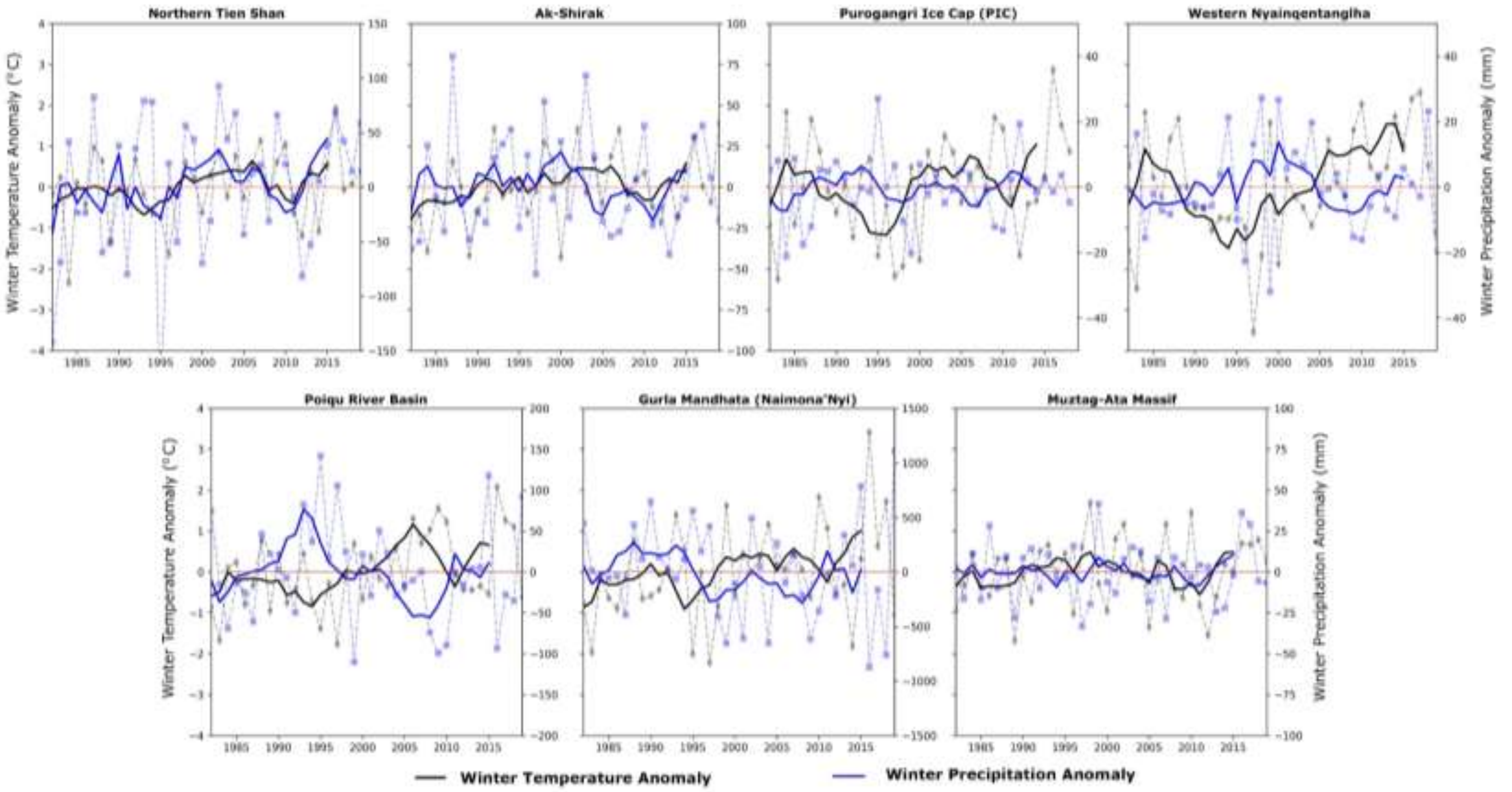

Supplementary Figure 22 Winter temperature and winter precipitation anomaly of ERA 5 Land gridded climate data for all regions. The thick lines represent the 5 years moving average.

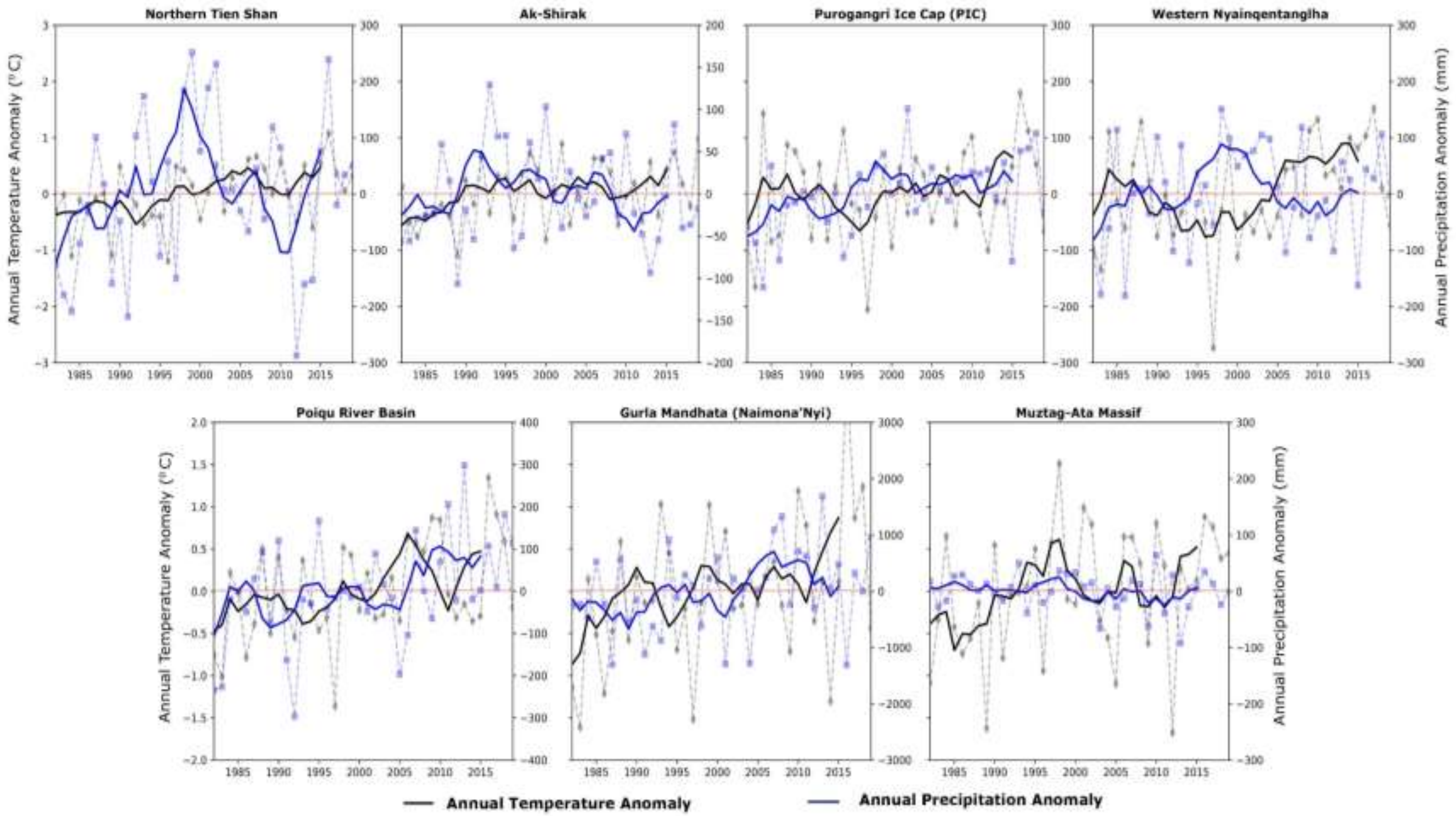

Supplementary Figure 23 Annual temperature and annual precipitation anomaly of ERA 5 Land gridded climate data for all regions. The thick lines represent the 5 years moving average.

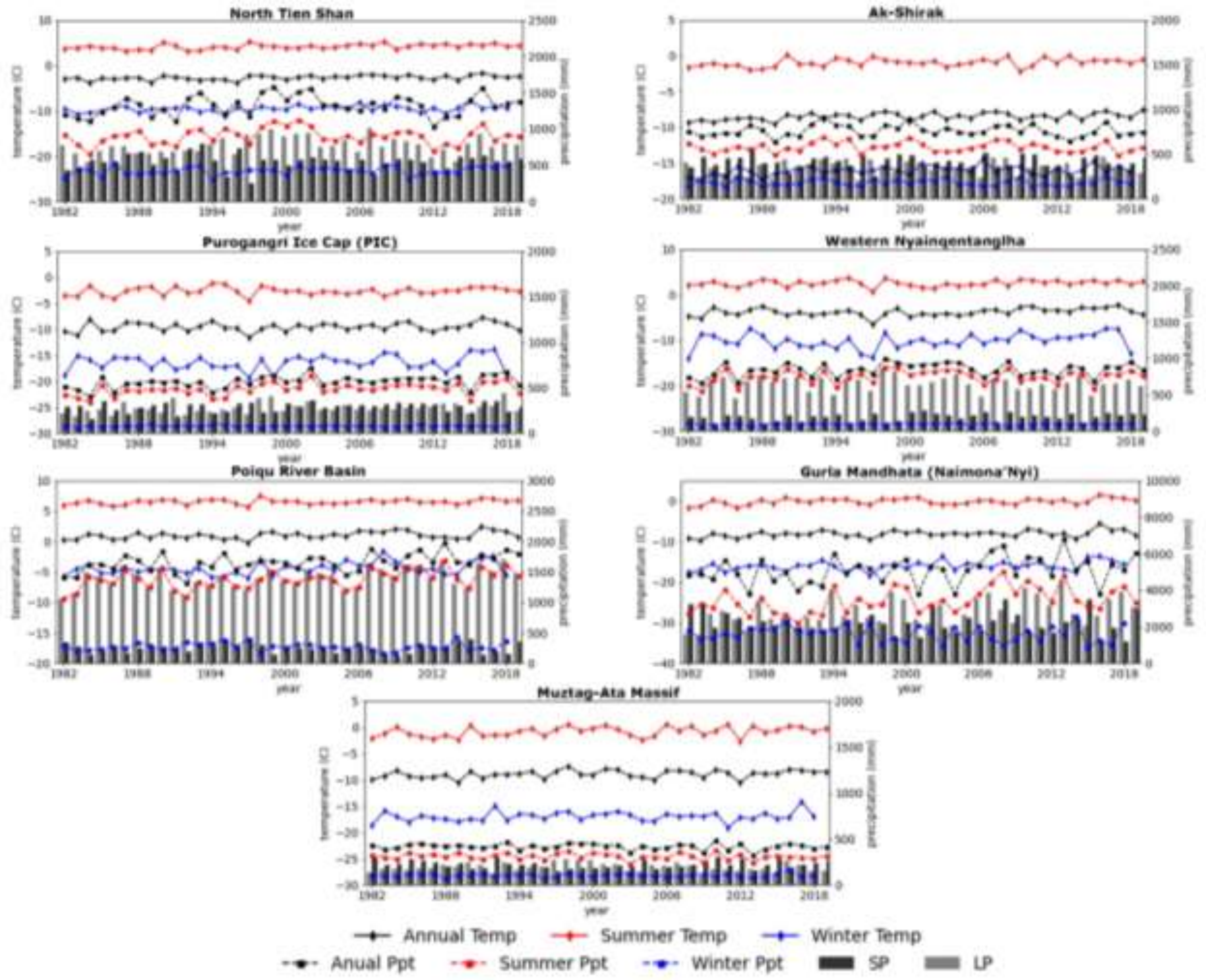

Supplementary Figure 24 ERA5 Land gridded climate data for all investigated regions (SP = Solid precipitation, LP = Liquid precipitation).

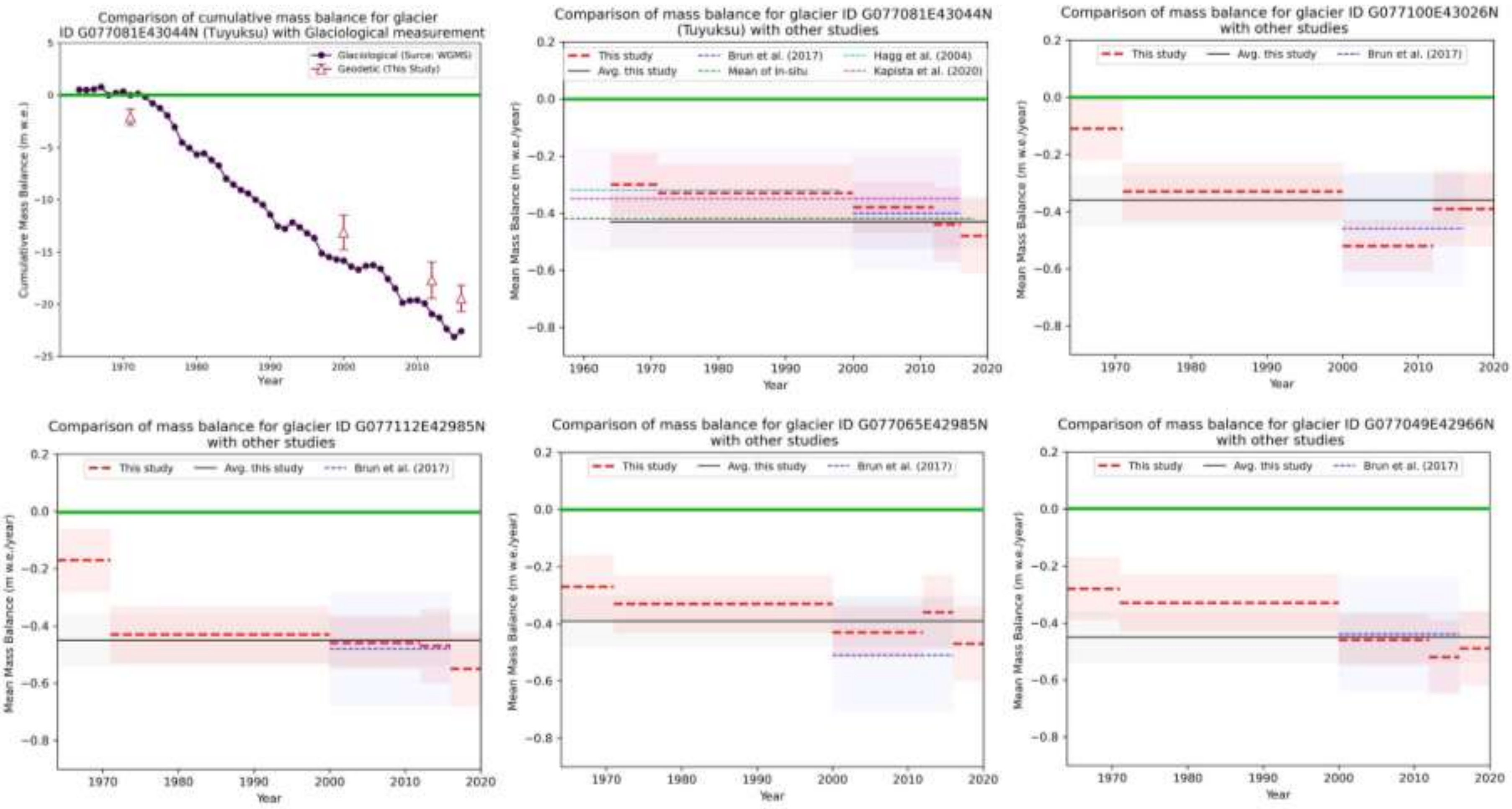

Supplementary Figure 25 Comparison with mass balances derived by other studies for individual glaciers in the Northern Tien Shan region (Overall represents the entire observation period of our study).

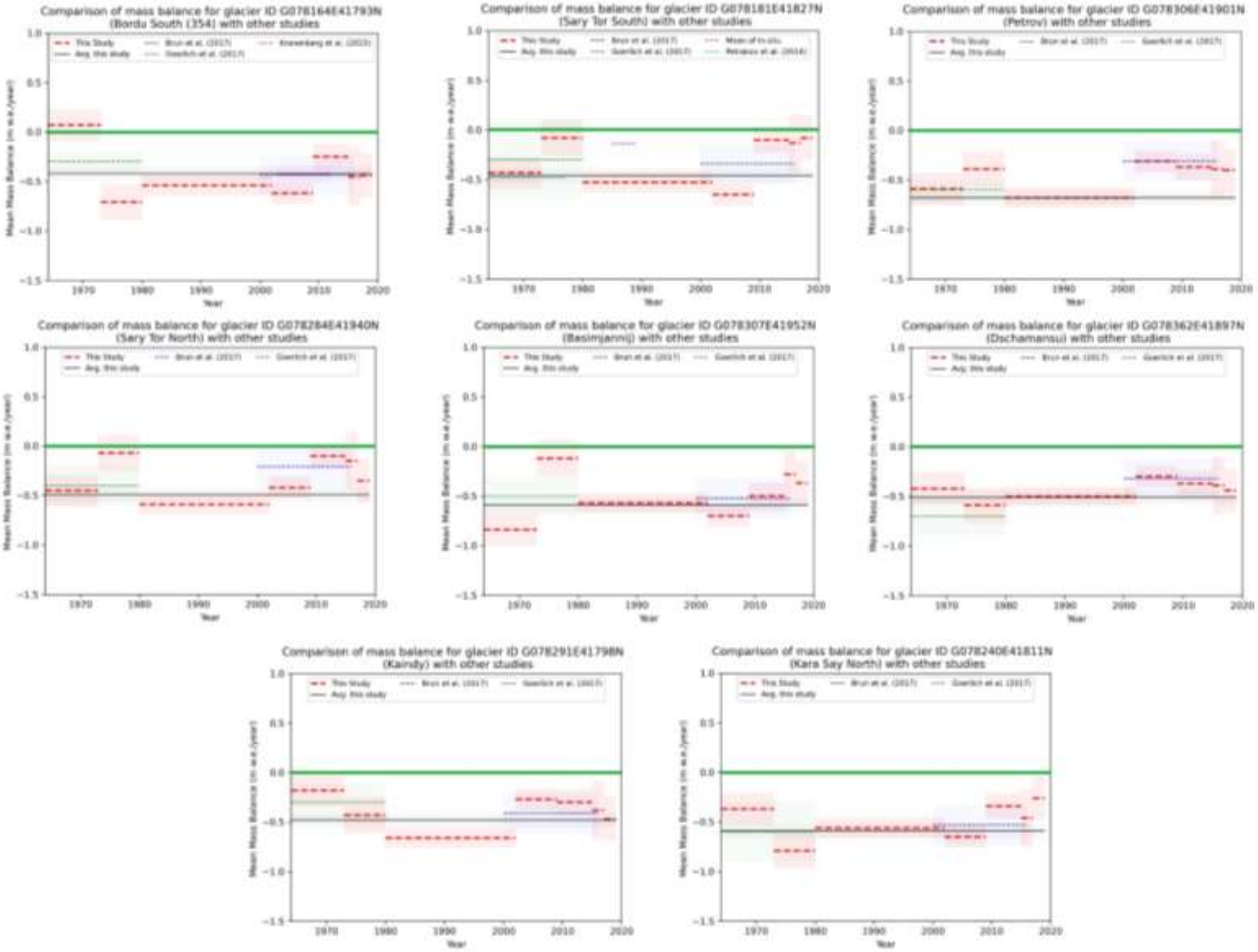

Supplementary Figure 26 Comparison with mass balances derived by other studies for individual glaciers in the Ak Shirak region (Overall represents the entire observation period of our study).

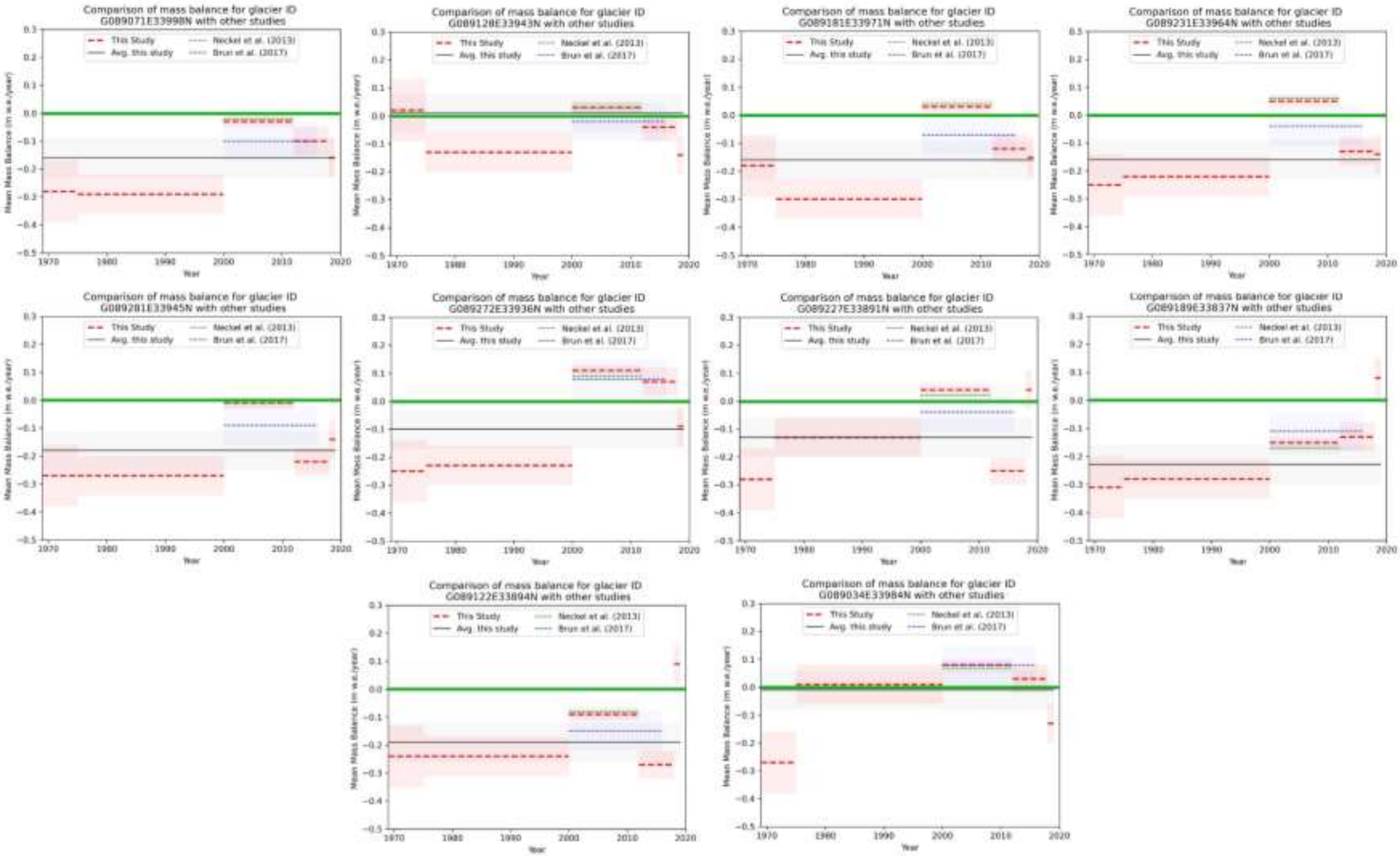

965

Supplementary Figure 27 Comparison with mass balances derived by other studies for individual glaciers in the Purogangri Ice Cap (PIC) region (Overall represents the entire observation period of our study).

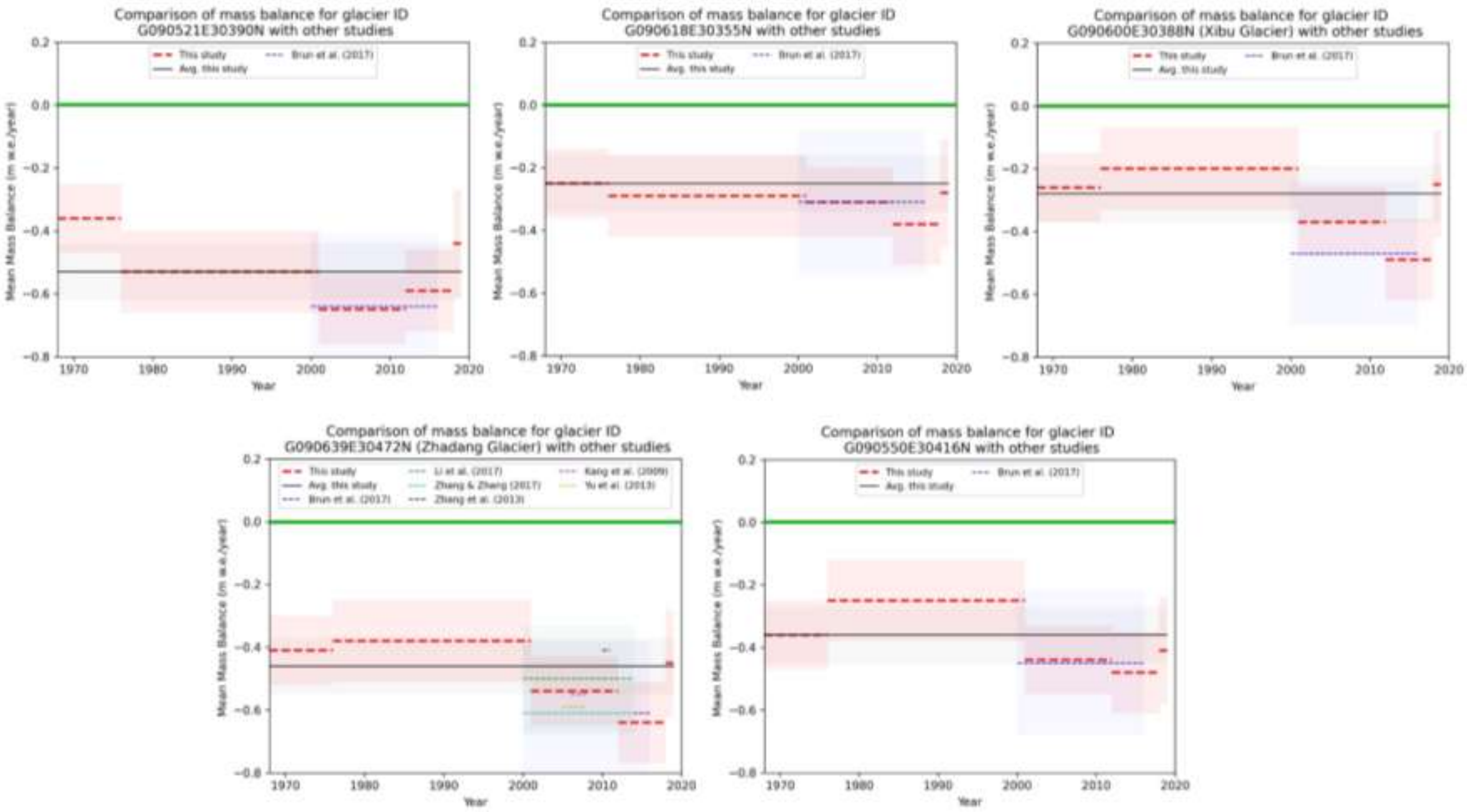

Supplementary Figure 28 Comparison with mass balances derived by other studies for individual glaciers in the Western Nyainqentanglha region (Overall represents the entire observation period of our study).

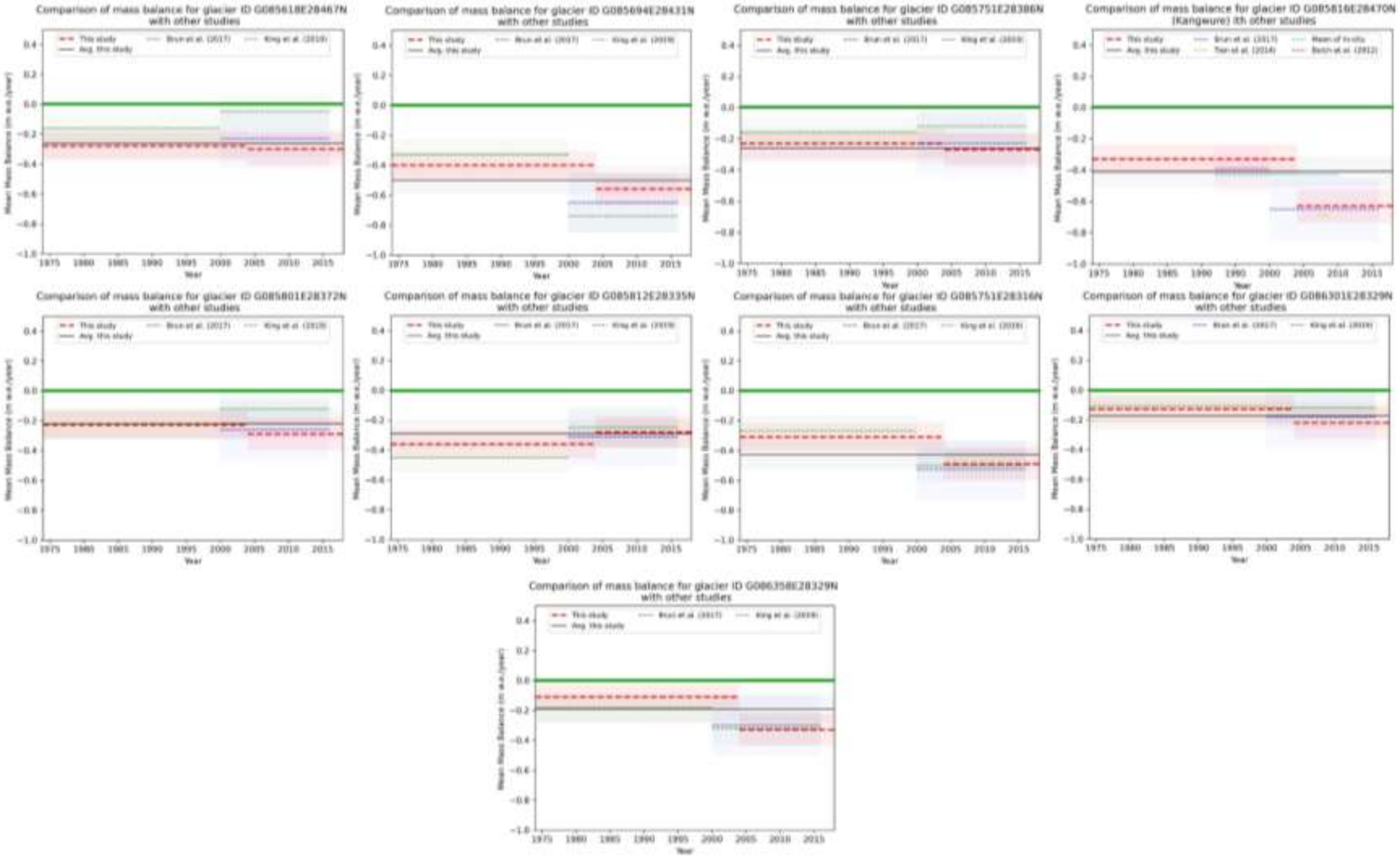

975

Supplementary Figure 29 Comparison with mass balances derived by other studies for individual glaciers in the Poiqu region (Overall represents the entire observation period of our study).

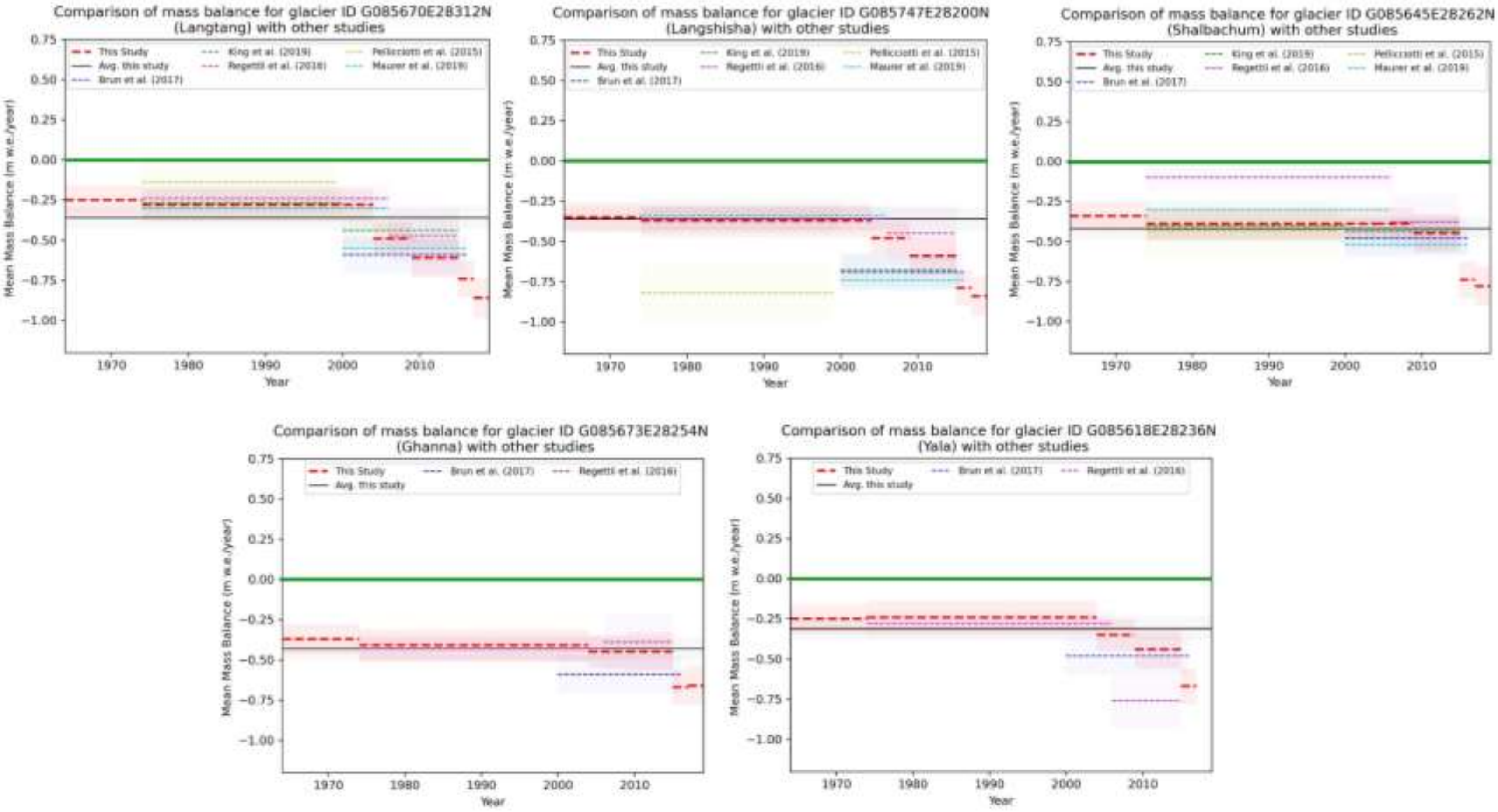

Supplementary Figure 30 Comparison with mass balances derived by other studies for individual glaciers in the Langtang region (Overall represents the entire observation period of our study).

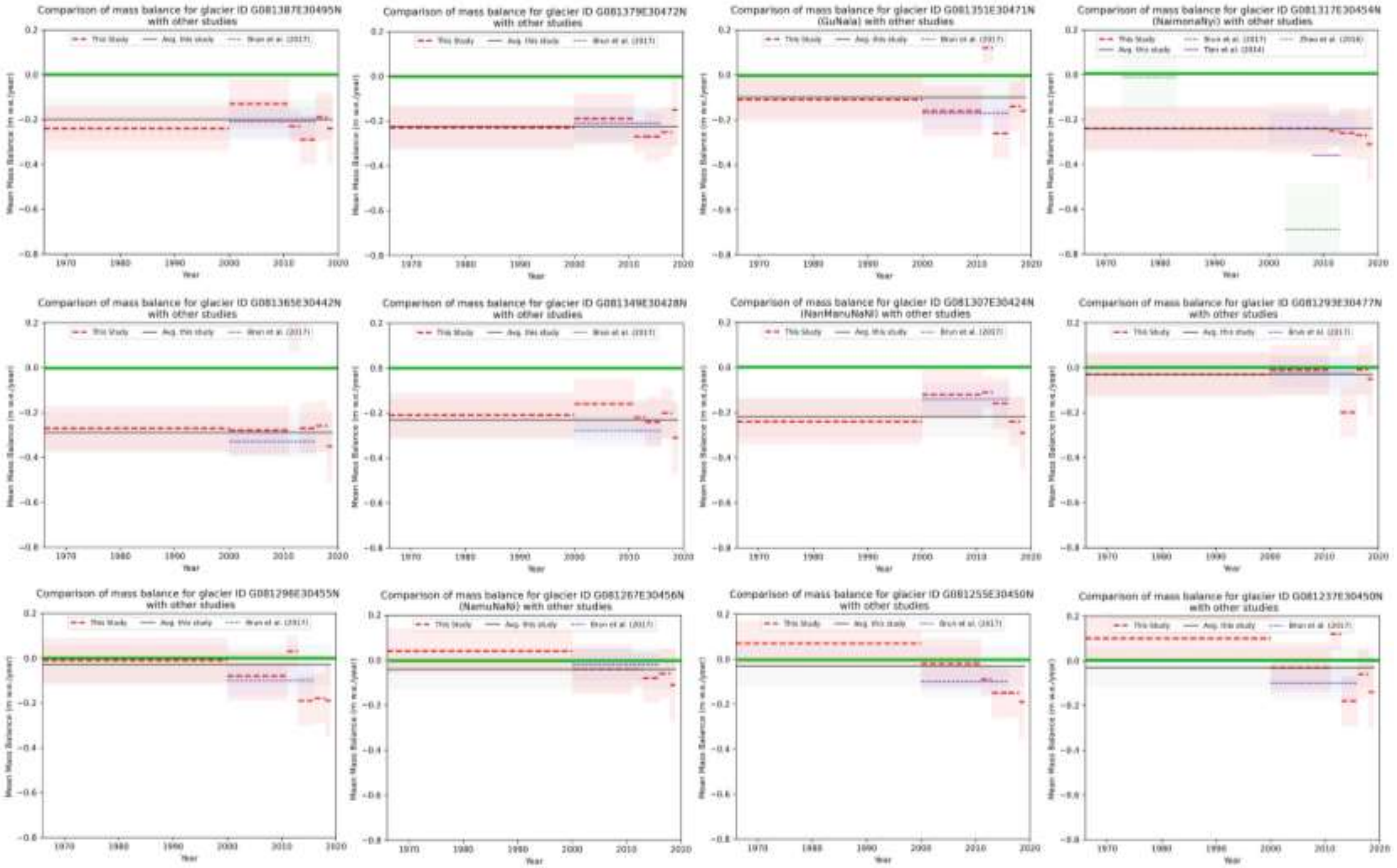

Supplementary Figure 31 Comparison with mass balances derived by other studies for individual glaciers in the Gurla Mandhata region (Overall represents the entire observation period of our study).

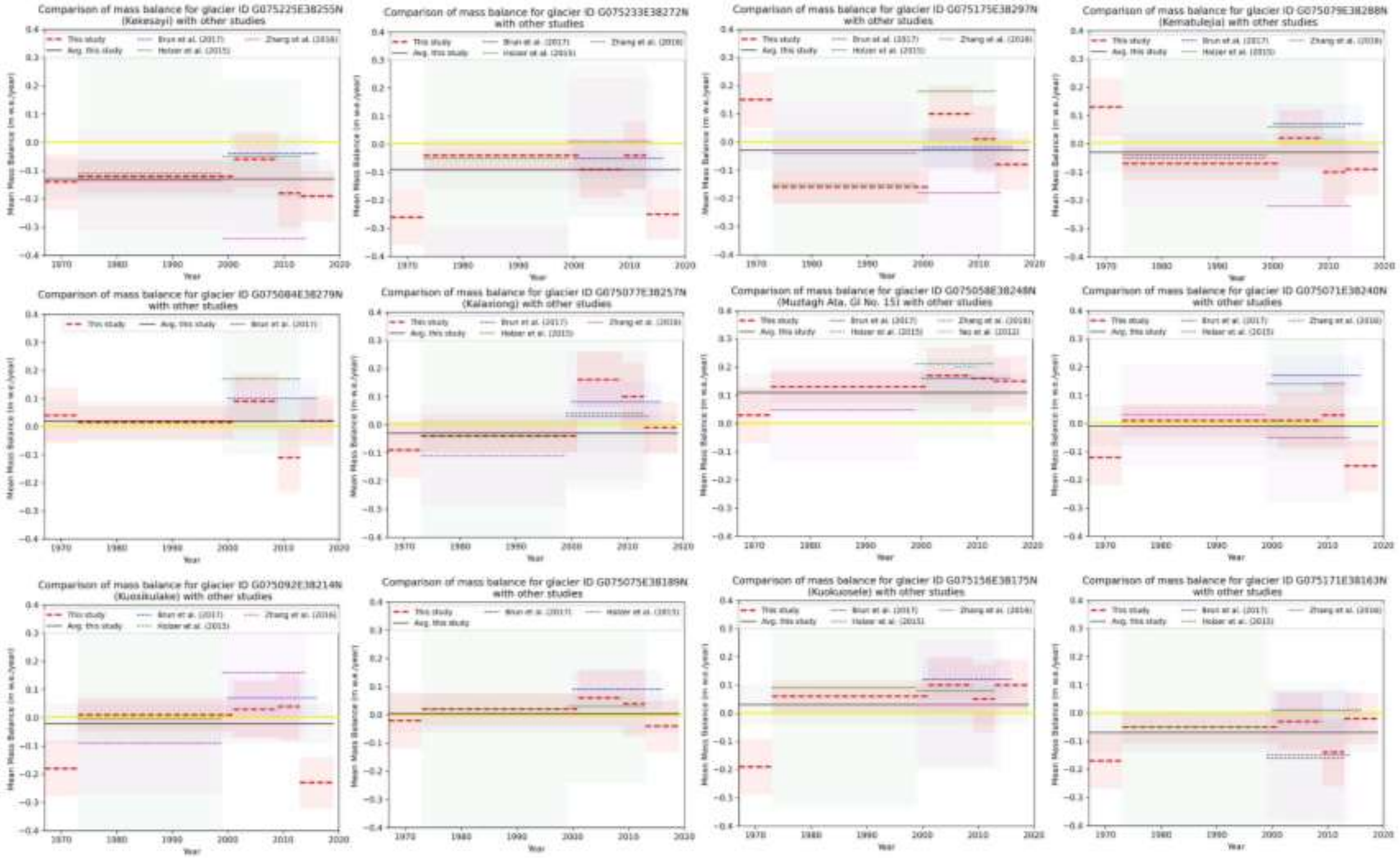

Supplementary Figure 32 Comparison with mass balances derived by other studies for individual glaciers in the Muztagh Ata Massif region (Overall represents the entire observation period of our study).

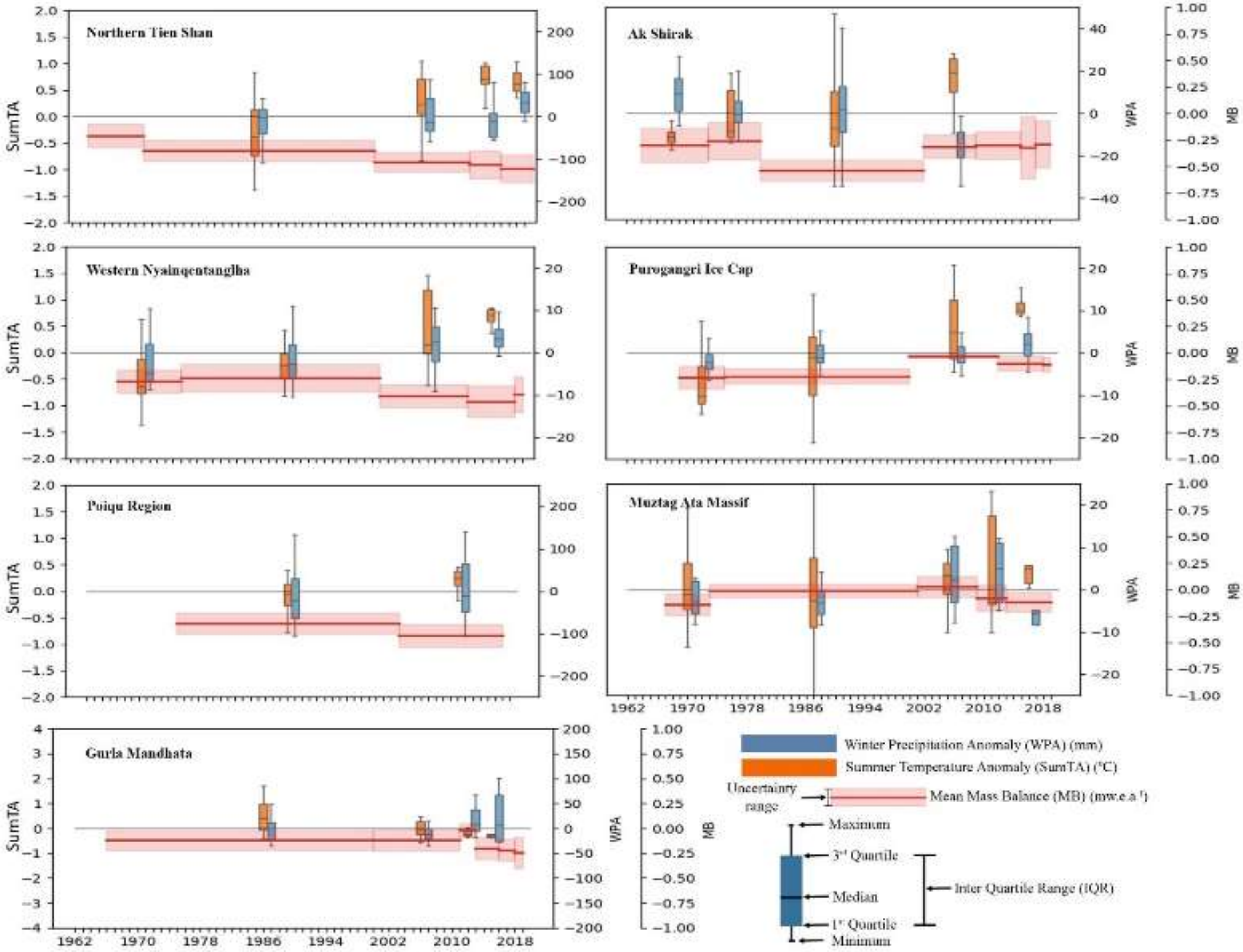

Supplementary Figure 33 Summer temperature and winter precipitation anomalies of the available weather station data along with geodetic glacier mass balance estimates for each of the study sites and time periods.

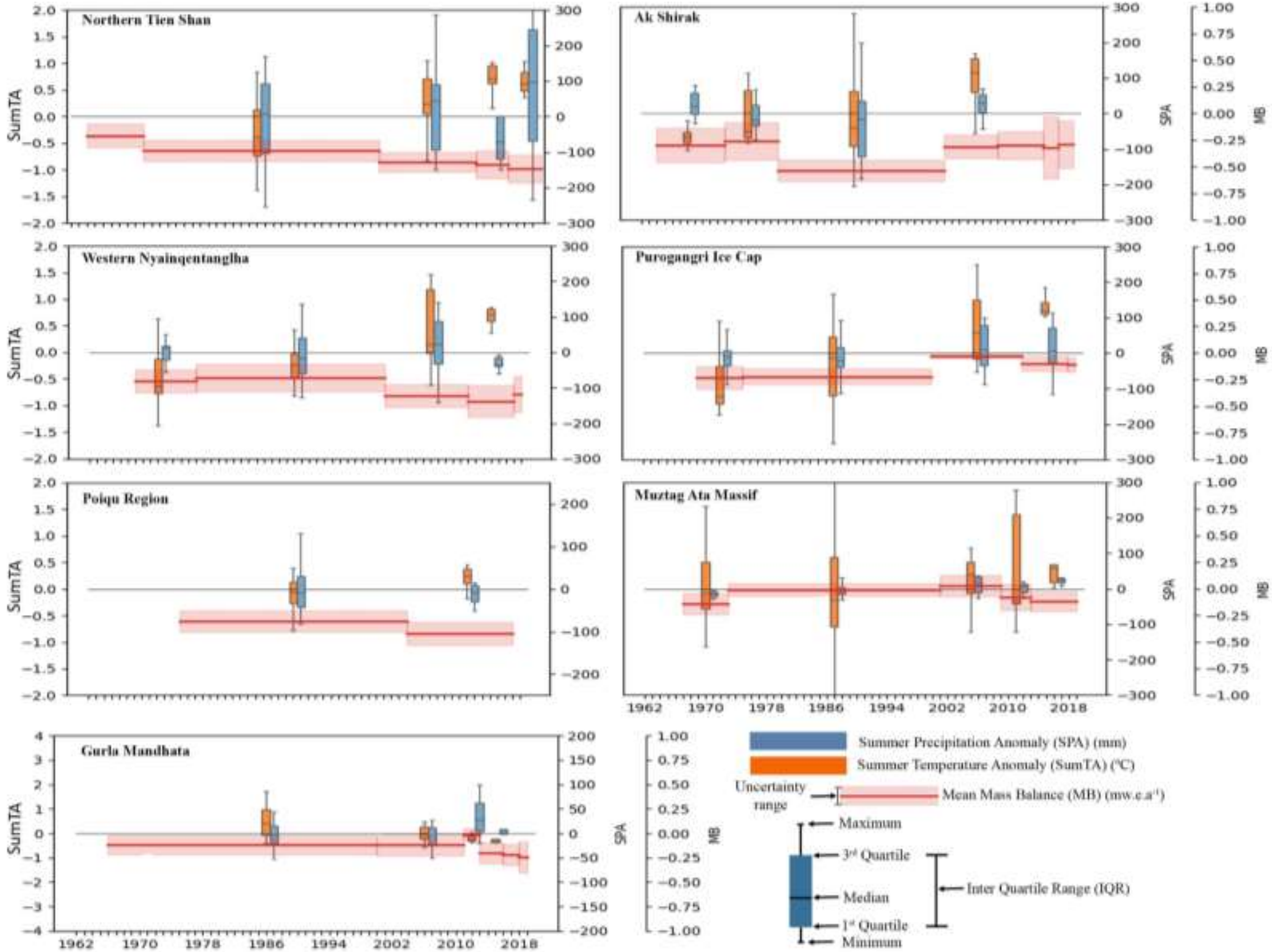

Supplementary Figure 34 Summer temperature and summer precipitation anomalies of the available weather station data along with geodetic glacier mass balance estimates for each of the study sites and time periods.

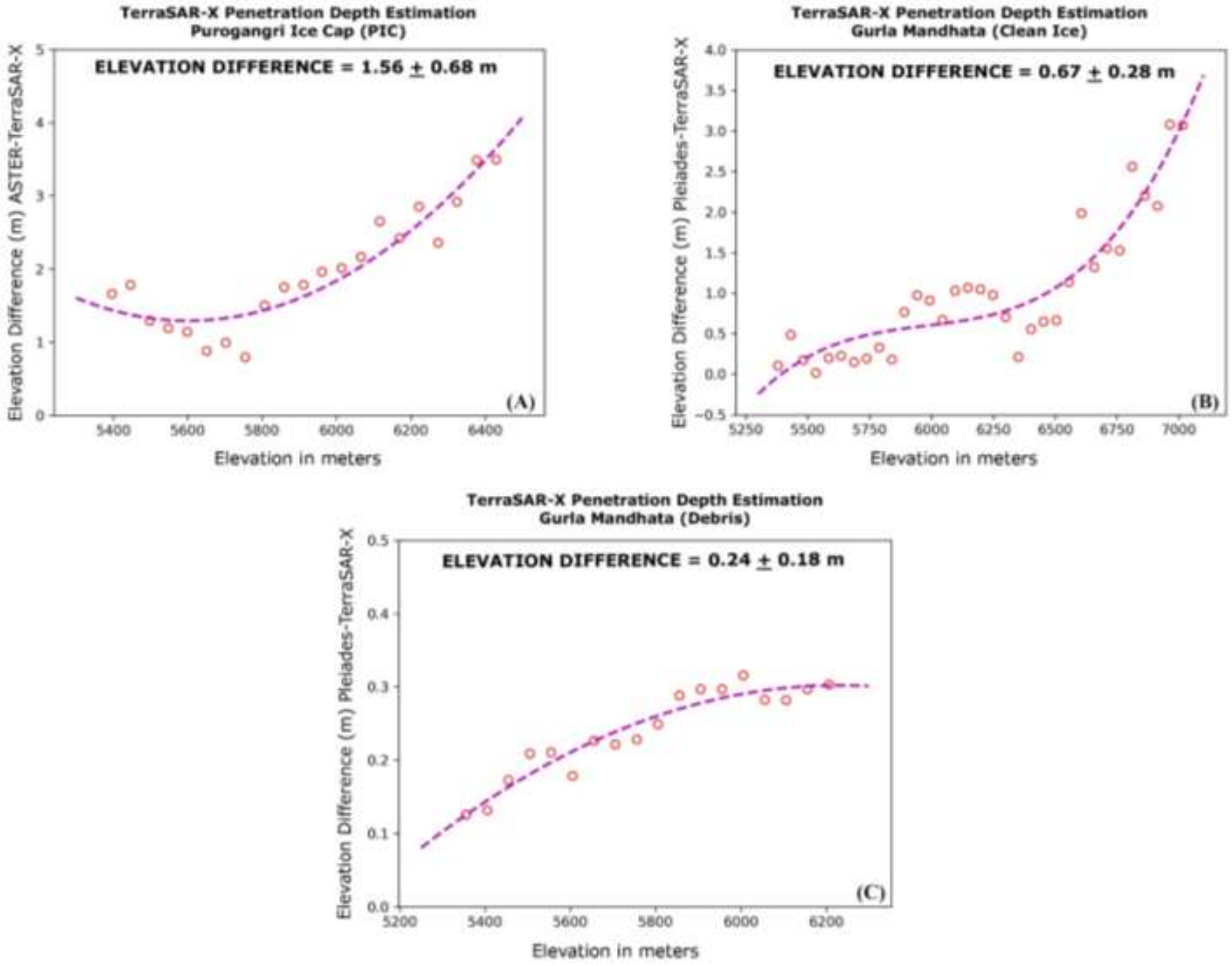

Supplementary Figure 35 Surface elevation difference calculated based on ASTER (acquisition date: 09.11.2012) and TanDEM-X (26.01.2012) DEMs for the Purogangri Ice Cap (PIC) region and based on Pléiades (26.10.2013) and TanDEM-X (28.10.2013) DEMs for the Gurla Mandhata region. The difference provides information about magnitude and characteristics of the X-band radar penetration depth.

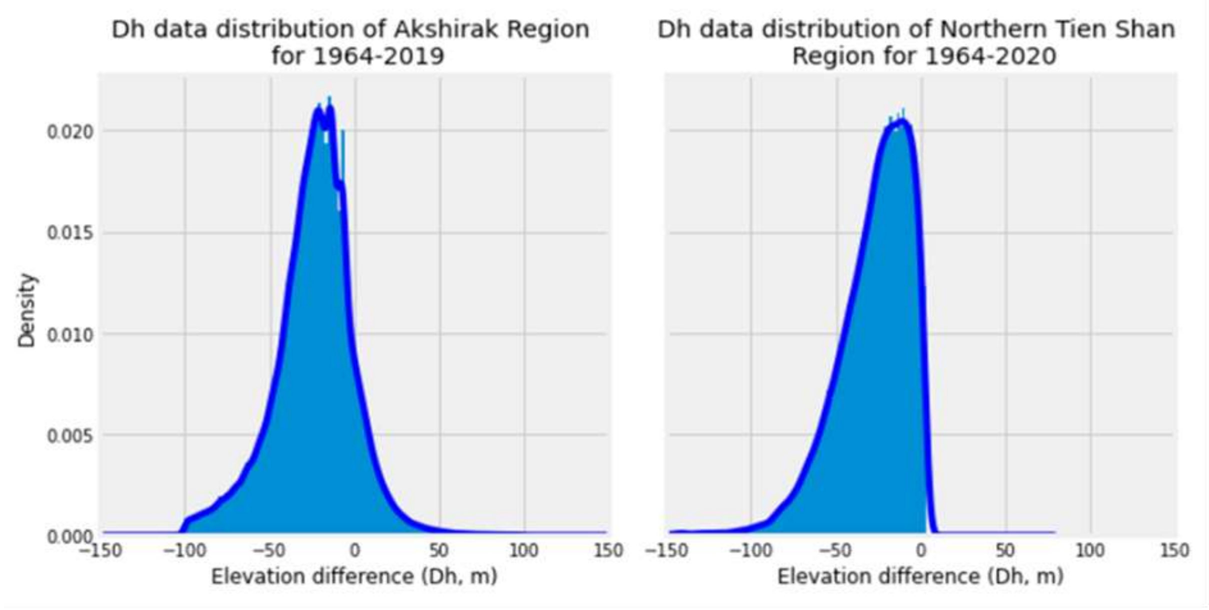

Supplementary Figure 36 Distribution of elevation difference data over the full study period in Ak-Shirak and Northern Tien Shan.

Supplementary Table 1. Details about the remotely sensed data used in this study (MB: Glacier Mass Balance; Mapping: Glacier Mapping).

**Region**	**Sensors**	**Date**	**Scenes/Product ID**	**Spatial resolution (m)**	**Usage**
**Northern Tien Shan**	Corona KH-4A	06-10-1964	DS1011-1007DA065 & DS1011-1007DF061	4	MB + Mapping
	Corona KH-4B	17-09-1971	DS1115-1104DF118, DS1115-1104DF119, DS1115-1104DA124 & DS1115-1104DA125	2	MB + Mapping
	ASTER	13-10-2000	AST_L1A#003_10132000060449_06242001163949	15	MB + Mapping
	GeoEye	09-08-2012	/GCP-12AUG09053708-P1BS_R1C1-053976794020_01_P001 & 12AUG09053708-P1BS_R2C1-053976794020_01_P001	0.5	MB + Mapping
	Pléiades	27-08-2016	DS_PHR1B_201608270545199 & DS_PHR1B_201608270545099	0.5	MB + Mapping
	Pléiades	16-09-2020	DS_PHR1A_202009160553131_FR1_PX_E077N43_0101_01880	0.5	MB + Mapping
**Ak-Shirak**	Corona KH-4	27-11-1964	DS1014-2134DA088, DS1014-2134DA089, DS1014-2134DA090, DS1014-2134DF084, DS1014-2134DF085 & DS1014-2134DF086	4	MB + Mapping
	Hexagon KH9	31-07-1973	DZB1206-500069L010001, DZB1206-500069L011001 & DZB1206-500069L012001	7.6	MB + Mapping
	Hexagon KH9	21-08-1980	DZB1216-500277L021001, DZB1216-500277L022001 & DZB1216-500277L023001	7.6	MB + Mapping
	ASTER	03-10-2002	AST_L1A_00310032002055218_20191127113618_21508	15	MB + Mapping
	ASTER	29-09-2009	AST_L1A_00309292009054509_20191127113618_21486 & AST_L1A_00309292009054500_20191127113601_20427	15	MB + Mapping
	SPOT-6	01-10-2015	DS_SPOT6_201510010514407_FR1_FR1_SE1_SE1_E078N42_01709	1.5	MB + Mapping
	SPOT-6	07-08-2017	DS_SPOT6_201708070515034_FR1_FR1_FR1_FR1_E078N42_01871	1.5	MB + Mapping
	ASTER	27-10-2019	AST_L1A_00310272019054443_20191127113628_23299 & AST_L1A_00310272019054451_20191127113618_21527	15	MB + Mapping
**Purogangri Ice Cap**	Corona KH-4B	13-12-1969	DS1108-2136DA033, DS1108-2136DA034, DS1108-2136DF027 & DS1108-2136DF028	4	MB + Mapping

	Hexagon KH-9	20-12-1975	DZB1211-500057L026001 & DZB1211-500057L027001	7.6	M**B** + Mapping
	SRTM-X	2000	E080N30_XSAR_DEM	30	MB
	Landsat-7	30-10-2000	LE07_L1TP_140036_20001030_20170209_01_T1	15	Mapping
	TanDEM-X	26-01-2012	TDM1_SAR__COS_BIST_SM_S_SRA_20120126T120304_20120126T120308 & TDM1_SAR__COS_BIST_SM_S_SRA_20120126T120307_20120126	3	MB
	ASTER	09-11-2012	AST_L1A#00311092012045114_11102012031108.hdf	15	Penetration + Mapping
	Pléiades	27-09-2018 & 03-10-2018	DS_PHR1B_201810030458515_FR1_PX_E089N33_0320_00596, DS_PHR1B_201810030458408_FR1_PX_E089N33_0223_02694 & DS_PHR1A_201809270454385_FR1_PX_E089N33_0422_02106	0.5	M**B** + Mapping
	Pléiades	30-8-2019, 31-08-2019 & 13-09-2019	DS_PHR1B_201908300502220_FR1_PX_E089N33_0221_01200, DS_PHR1A_201908310454596_FR1_PX_E089N33_0422_02106 & DS_PHR1B_201909130454493_FR1_PX_E089N33_0224_01804	0.5	M**B** + Mapping
**Western Nyainqentanglha**	Corona KH-4B	15-11-1968	DS1105-2184DA078, DS1105-2184DA079, DS1105-2184DA080, DS1105-2184DF071, DS1105-2184DF072 & DS1105-2184DF073	4	M**B** + Mapping
	Hexagon KH-9	07-01-1976	DZB1211-500125L010001 & DZB1211-500125L011001	7.6	M**B** + Mapping
	ASTER	20-11-2001	AST_L1A#003_11202001045037_11302001114506 &	15	M**B** + Mapping
	Landsat-7 ETM+	04-12-2012	LE07_L1TP_138039_20121204_20161128_01_T1_B8	15	Mapping
	TanDEM-X	05-12-2012	TDM1_SAR__COS_BIST_SM_S_SRA_20121205T115340_20121205T115348	3	MB
	ASTER	24-10-2012	AST_L1A#00310242012045210_10252012032357	15	Penetration + Mapping
	Pléiades	11-10-2018	DS_PHR1A_201810110448021_FR1_PX_E090N30_0710_01728 & DS_PHR1A_201810110448119_FR1_PX_E090N30_0910_02106	0.5	M**B** + Mapping
	Pléiades	29-10-2019, 11-11-2019 & 12-11-2019	DS_PHR1A_201911120444266_FR1_PX_E090N30_0810_02258, DS_PHR1B_201911110451504_FR1_PX_E090N30_0810_02258 & DS_PHR1A_201910290452058_FR1_PX_E090N30_0910_01804	0.5	M**B** + Mapping

**Poiqu region**	Hexagon KH-9	23-11-1974	DZB1209-500101L006001 & DZB1209-500101L007001	7.6	M**B** + Mapping
	ASTER	04-10-2004	AST_L1A_00309232004045839_20191216041641_19157, AST_L1A_00309232004045848_20191216041641_19161, AST_L1A_00310162004050426_20191216041651_19216 & AST_L1A_00310162004050435_20191216041651_19218	15	M**B** + Mapping
	Pléiades	25-09-2018 to 04-11-2018	DS_PHR1A_201809250511185_FR1_PX_E085N28_1110_03316, DS_PHR1B_201809260504010_FR1_PX_E086N28_0105_05430, DS_PHR1A_201809270456239_FR1_PX_E086N28_0510_01804, DS_PHR1B_201810030500231_FR1_PX_E086N28_0309_02182, DS_PHR1B_201810150507481_FR1_PX_E085N28_1103_02921, DS_PHR1B_201810150507593_FR1_PX_E086N27_0123_00862, DS_PHR1B_201810220504174_FR1_PX_E086N28_0102_01804, DS_PHR1B_201811030511344_FR1_PX_E085N28_0715_03053, DS_PHR1A_201811040503518_FR1_PX_E085N28_1108_01768 & DS_PHR1A_201810020508169_FR1_PX_E085N28_0911_05204	0.5	M**B** + Mapping
	Pléiades	14-10-2019, 27-10-2019 & 18-12-2019	DS_PHR1B_201910140507258_FR1_PX_E085N28_1108_04054, DS_PHR1A_201910270507451_FR1_PX_E085N28_0905_00746 & DS_PHR1A_201912180507284_FR1_PX_E085N28_0908_01728	0.5	M**B** + Mapping
**Langtang region**	Corona KH-4A	26-11-1964	DS1014-2118DA188, DS1014-2118DA189, DS1014-2118DA190, DS1014-2118DA191, DS1014-2118DF187, DS1014-2118DF188, DS1014-2118DF189	4	M**B** + Mapping
	Hexagon KH-9	23-11-1974	DZB1209-500101L006001 & DZB1209-500101L007001	7.6	M**B** + Mapping
	ASTER	04-10-2004	ST_L1A_00310162004050426_20191216041651_19216 & AST_L1A_00310162004050435_20191216041651_19218	15	M**B** + Mapping
	ASTER	23-10-2009	AST_L1A#00310232009045933_10262009104316.hdf, AST_L1A#00310232009045924_10262009104307.hdf	15	M**B** + Mapping
	SPOT 7	06-10-2015	DS_SPOT7_201510060438227_FR1_FR1_SE1_SE1_E086N28_01303	1.5	M**B** + Mapping
	ASTER	20-10-2017	AST_L1A#00310202017050548_10212017083708.hdf	15	M**B** + Mapping
	ASTER	26-10-2019	AST_L1A#00310262019050517_10272019083336.hdf	15	M**B** + Mapping

**Gurla-Mandhata (Naimona'Nyi)**	Corona KH-4A	09-11-1966	DS1037-1007DA075 & DS1037-1007DF076	4	**MB** + Mapping
	ASTER	26-10-2000	AST_L1A#003_10262000053706_07052001062219	15	**MB** + Mapping
	ASTER	25-10-2011	AST_L1A#00310252011052300_10262011041614	15	**MB** + Mapping
	Pléiades	12-10-2013, 18-10-2013 & 26-10-2013	DS_PHR1B_201310260519175_SE1_PX_E081N30_0609_03316, DS_PHR1A_201310180529398_SE1_PX_E081N30_0411_01787 & DS_PHR1B_201310120526421_SE1_PX_E081N30_0409_03164	0.5	**MB** + Mapping
	TanDEM-X	28-10-2013	TDM1_SAR__COS_BIST_SM_S_SRA_20131028T003823_20131028T003828	3	Penetration
	HMA DEM	04-09-2016	HMA_DEM8m_AT_20160904_0822_102001005554CF00_1020010053E4E800	8	MB
	Landsat-7	23-11-2016	LE07_L1TP_144039_20161123_20170302_01_T1	15	Mapping
	Pléiades	16-09-2018 & 06-10-2018	DS_PHR1A_201809160529541_FR1_PX_E081N30_0311_01124 & DS_PHR1B_201810060526179_FR1_PX_E081N30_0411_01542	0.5	**MB** + Mapping
	Pléiades	16-09-2019	DS_PHR1B_201909160522124_FR1_PX_E081N30_0411_01578 & DS_PHR1B_201909160522213_FR1_PX_E081N30_0311_01124	0.5	**MB** + Mapping
**Muztagh-Ata**	Corona KH-4A	10-08-1967	DS1043-1039DA072, DS1043-1039DA073, DS1043-1039DF070 & DS1043-1039DF071	4	**MB** + Mapping
	Hexagon KH-9	04-08-1973	DZB1206-500082L017001, DZB1206-500082L018001 & DZB1206-500082L019001	7.6	**MB** + Mapping
	ASTER	17-11-2001	AST_L1A#003_11172001055620_11282001134310 & AST_L1A#003_11172001055629_1128200113432	15	MB + Mapping
	ALOS PRISM	10-09-2009	ALPSMF193302775, ALPSMN193302830 & ALPSMB193302885	2.5	**MB** + Mapping
	Pléiades	19-06-2013, 20-06-2013 & 03-08-2013	DS_PHR1A_201306200550211_FR1_PX_E075N38_0306_03390, DS_PHR1B_201306190558223_FR1_PX_E075N38_0106_02977 & DS_PHR1B_201308030602439_SE1_PX_E075N38_0104_01352	0.5	**MB** + Mapping
	Pléiades	05-09-2019 & 11-09-2019	DS_PHR1B_201909050554111_FR1_PX_E075N38_0306_03316 & DS_PHR1A_201909110558233_FR1_PX_E075N38_0106_02862	0.5	**MB** + Mapping

Supplementary Table 2. Elevation change (ΔH) and mass budget (MB) of selected glaciers and all glaciers in the North Tien Shan region for the different time periods.

Sl. No.	Glacier	Component	1964-1971	1971-2000	2000-2012	2012-2016	2016-2020	1971-2012	1971-2016	1964-2020
**1**	**G077081E430 44N (Tuyuksu)**	ΔH (m)	-2.51 ± 1.08	-11.39 ± 3.8	-5.34 ± 1.50	-2.07 ± 0.68	-2.24 ± 0.71	-17.03 ± 3.5	-17.87 ± 4.2	-28.21 ± 6.7
		MB (m w.e.a^{-1})	-0.30 ± 0.11	-0.33 ± 0.10	-0.38 ± 0.09	-0.44 ± 0.13	-0.48 ± 0.13	-0.35 ± 0.07	-0.34 ± 0.07	-0.43 ± 0.09
**2**	**G077100E430 26N**	ΔH (m)	-0.90 ± 1.08	-11.18 ± 3.8	-7.29 ± 1.50	-1.83 ± 0.68	-1.83 ± 0.71	-19.06 ± 3.5	-19.34 ± 4.2	-23.94 ± 6.7
		MB (m w.e.a^{-1})	-0.11 ± 0.11	-0.33 ± 0.10	-0.52 ± 0.09	-0.39 ± 0.13	-0.39 ± 0.13	-0.40 ± 0.07	-0.37 ± 0.07	-0.36 ± 0.09
**3**	**G077112E429 85N**	ΔH (m)	-1.41 ± 1.08	-14.73 ± 3.8	-6.54 ± 1.50	-2.22 ± 0.68	-2.58 ± 0.71	-24.28 ± 3.5	-25.96 ± 4.2	-29.62 ± 6.7
		MB (m w.e.a^{-1})	-0.17 ± 0.11	-0.43 ± 0.10	-0.46 ± 0.09	-0.47 ± 0.13	-0.55 ± 0.13	-0.50 ± 0.07	-0.49 ± 0.07	-0.45 ± 0.09
**4**	**G077065E429 85N**	ΔH (m)	-2.21 ± 1.08	-11.09 ± 3.8	-6.12 ± 1.50	-1.69 ± 0.68	-1.75 ± 0.71	-18.25 ± 3.5	-19.82 ± 4.2	-26.02 ± 6.7
		MB (m w.e.a^{-1})	-0.27 ± 0.11	-0.33 ± 0.10	-0.43 ± 0.09	-0.36 ± 0.13	-0.47 ± 0.13	-0.38 ± 0.07	-0.37 ± 0.07	-0.39 ± 0.09
**5**	**G077049E429 66N**	ΔH (m)	-2.33 ± 1.08	-11.35 ± 3.8	-6.51 ± 1.50	-2.43 ± 0.68	-2.29 ± 0.71	-20.84 ± 3.5	-21.95 ± 4.2	-29.88 ± 6.7
		MB (m w.e.a^{-1})	-0.28 ± 0.11	-0.33 ± 0.10	-0.46 ± 0.09	-0.52 ± 0.13	-0.49 ± 0.13	-0.43 ± 0.07	-0.41 ± 0.07	-0.45 ± 0.09
**OVERALL (-1.16 ± 0.21 Gt)**		ΔH (m)	-1.45 ± 1.08	-10.98 ± 3.8	-6.12 ± 1.50	-2.11 ± 0.68	-2.30 ± 0.71	-18.64 ± 3.5	-19.91 ± 4.2	-26.12 ± 6.7
		MB (m w.e.a^{-1})	-0.18 ± 0.11	-0.32 ± 0.10	-0.43 ± 0.09	-0.45 ± 0.13	-0.49 ± 0.13	-0.39 ± 0.07	-0.38 ± 0.07	-0.40 ± 0.09

Supplementary Table 3. Elevation change (ΔH) and mass budget (MB) of selected glaciers and all glaciers in the Ak Shirak region for the different time periods (++surge type glaciers, ##lake-terminating glaciers and bold font indicates positive mass balance)

Sl. No.	Glacier	component	1964-1973	1973-1980	1980-2002	2002-2009	2009-2015	2015-2017	2017-2019	1964-2019
**1**	**G078164E41793N (Bordu South)**	ΔH (m)	**+0.71 ± 1.92**	-5.82 ± 1.70	-13.89 ± 3.00	-5.11 ± 1.04	-1.80 ± 1.05	-1.06 ± 0.84	-1.04 ± 0.60	-27.79 ± 5.31
		MB (m w.e.a^{-1})	**+0.07 ± 0.16**	-0.71 ± 0.18	-0.54 ± 0.10	-0.62 ± 0.11	-0.25 ± 0.13	-0.45 ± 0.30	-0.44 ± 0.23	-0.43 ± 0.07
**2**	**G078181E41827N (Sary Tor South)**	ΔH (m)	-4.51 ± 1.92	-0.64 ± 1.70	-13.78 ± 3.00	-5.33 ± 1.04	-0.70 ± 1.05	-0.31 ± 0.84	-0.18 ± 0.60	-29.97 ± 5.31
		MB (m w.e.a^{-1})	-0.43 ± 0.16	-0.08 ± 0.18	-0.53 ± 0.10	-0.65 ± 0.11	-0.10 ± 0.13	-0.13 ± 0.30	-0.08 ± 0.23	-0.46 ± 0.07
**3++**	**G078205E41827N (Davidov)**	ΔH (m)	-6.39 ± 1.92	**+1.83 ± 1.70**	-16.26 ± 3.00	-	-	-	-	-
		MB (m w.e.a^{-1})	-0.60 ± 0.16	**+0.22 ± 0.18**	-0.63 ± 0.10	-	-	-	-	-
**4##**	**G078306E41901N (Petrov)**	ΔH (m)	-6.23 ± 1.92	-3.18 ± 1.70	-17.58 ± 3.00	-2.54 ± 1.04	-2.61 ± 1.05	-0.91 ± 0.84	-0.95 ± 0.60	-43.74 ± 5.31
		MB (m w.e.a^{-1})	-0.59 ± 0.16	-0.39 ± 0.18	-0.68 ± 0.10	-0.31 ± 0.11	-0.37 ± 0.13	-0.39 ± 0.30	-0.40 ± 0.23	-0.68 ± 0.07
**5++**	**G078284E41940N (Sary Tor North)**	ΔH (m)	-4.77 ± 1.92	-0.54 ± 1.70	-15.36 ± 3.00	-3.50 ± 1.04	-0.70 ± 1.05	-0.36 ± 0.84	-0.82 ± 0.60	-31.96 ± 5.31
		MB (m w.e.a^{-1})	-0.45 ± 0.16	-0.07 ± 0.18	-0.59 ± 0.10	-0.42 ± 0.11	-0.10 ± 0.13	-0.15 ± 0.30	-0.35 ± 0.23	-0.49 ± 0.07
**6++**	**G078307E41952N (Basimjannij)**	ΔH (m)	-8.91 ± 1.92	-0.99 ± 1.70	-14.70 ± 3.00	-5.80 ± 1.04	-3.51 ± 1.05	-0.65 ± 0.84	-0.88 ± 0.60	-38.06 ± 5.31
		MB (m w.e.a^{-1})	-0.84 ± 0.16	-0.12 ± 0.18	-0.57 ± 0.10	-0.70 ± 0.11	-0.50 ± 0.13	-0.28 ± 0.30	-0.37 ± 0.23	-0.59 ± 0.07
**7**	**G078362E41897N (Dschamansu)**	ΔH (m)	-4.43 ± 1.92	-4.85 ± 1.70	-12.92 ± 3.00	-2.48 ± 1.04	-2.62 ± 1.05	-0.93 ± 0.84	-1.03 ± 0.60	-33.19 ± 5.31
		MB (m w.e.a^{-1})	-0.42 ± 0.16	-0.59 ± 0.18	-0.50 ± 0.10	-0.30 ± 0.11	-0.37 ± 0.13	-0.39 ± 0.30	-0.44 ± 0.23	-0.51 ± 0.07
**8++**	**G078291E41798N (Kaindy)**	ΔH (m)	-1.88 ± 1.92	-3.57 ± 1.70	-17.10 ± 3.00	-3.04 ± 1.04	-2.14 ± 1.05	-0.89 ± 0.84	-1.10 ± 0.60	-31.09 ± 5.31
		MB (m w.e.a^{-1})	-0.18 ± 0.16	-0.43 ± 0.18	-0.66 ± 0.10	-0.27 ± 0.11	-0.30 ± 0.13	-0.38 ± 0.30	-0.47 ± 0.23	-0.48 ± 0.07
**9++**	**G078240E41811N (Kara Say North)**	ΔH (m)	-3.92 ± 1.92	-6.51 ± 1.70	-14.57 ± 3.00	-5.33 ± 1.04	-2.39 ± 1.05	-1.09 ± 0.84	-0.61 ± 0.60	-38.11 ± 5.31
		MB (m w.e.a^{-1})	-0.37 ± 0.16	-0.79 ± 0.18	-0.56 ± 0.10	-0.65 ± 0.11	-0.34 ± 0.13	-0.46 ± 0.30	-0.26 ± 0.23	-0.59 ± 0.07
**Overall (-8.09 ± 1.08 Gt)**		ΔH (m)	-3.21 ± 1.92	-2.15 ± 1.70	-13.97 ± 3.00	-2.59 ± 1.04	-2.12 ± 1.05	-0.76 ± 0.84	-0.68 ± 0.60	-26.14 ± 5.31
		MB (m w.e.a^{-1})	-0.30 ± 0.16	-0.26 ± 0.18	-0.54 ± 0.10	-0.31 ± 0.11	-0.30 ± 0.13	-0.32 ± 0.30	-0.29 ± 0.23	-0.40 ± 0.07

Supplementary Table 4. Elevation change (ΔH) and mass budget (MB) of selected glaciers and all glaciers in the Purogangri Ice Cap (PIC) region for the different time periods (bold font indicates positive mass balance).

Sl. No.	Glacier	Component	1969-1975	1975-2000	2000-2012	2012-2018	2018-2019	1969-2019
**1**	**G089071E33998N**	ΔH (m)	-1.95 ± 0.93	-8.67 ± 2.21	-0.41 ± 0.36	-0.70 ± 0.42	-0.19 ± 0.10	-9.41 ± 5.11
		MB (m w.e.a^{-1})	-0.28 ± 0.11	-0.29 ± 0.07	-0.03 ± 0.02	-0.10 ± 0.05	-0.16 ± 0.08	-0.16 ± 0.07
**2**	**G089128E33943N**	ΔH (m)	+0.16 ± 0.93	-3.72 ± 2.21	+0.39 ± 0.36	-0.26 ± 0.42	-0.16 ± 0.10	+0.74 ± 5.11
		MB (m w.e.a^{-1})	**+0.02 ± 0.11**	-0.13 ± 0.06	**+0.03 ± 0.02**	-0.04 ± 0.05	-0.14 ± 0.08	**+0.01 ± 0.07**
**3**	**G089181E33971N**	ΔH (m)	-1.24 ± 0.93	-8.77 ± 2.21	+0.45 ± 0.36	-0.83 ± 0.42	-0.18 ± 0.10	-9.21 ± 5.11
		MB (m w.e.a^{-1})	-0.18 ± 0.11	-0.30 ± 0.07	**+0.03 ± 0.02**	-0.12 ± 0.05	-0.15 ± 0.08	-0.16 ± 0.07
**4**	**G089231E33964N**	ΔH (m)	-1.78 ± 0.93	-6.44 ± 2.21	+0.68 ± 0.36	-0.91 ± 0.42	-0.17 ± 0.10	-9.47 ± 5.11
		MB (m w.e.a^{-1})	-0.25 ± 0.11	-0.22 ± 0.07	**+0.05 ± 0.02**	-0.13 ± 0.05	-0.14 ± 0.08	-0.16 ± 0.07
**5**	**G089281E33945N**	ΔH (m)	-1.88 ± 0.93	-7.95 ± 2.21	-0.19 ± 0.36	-1.58 ± 0.42	-0.16 ± 0.10	-10.55 ± 5.11
		MB (m w.e.a^{-1})	-0.27 ± 0.11	-0.27 ± 0.07	-0.01 ± 0.02	-0.22 ± 0.05	-0.14 ± 0.08	-0.18 ± 0.07
**6**	**G089272E33936N**	ΔH (m)	-1.79 ± 0.93	-6.69 ± 2.21	+1.50 ± 0.36	+0.49 ± 0.42	-0.11 ± 0.10	-5.98 ± 5.11
		MB (m w.e.a^{-1})	-0.25 ± 0.11	-0.23 ± 0.07	**+0.11 ± 0.02**	**+0.07 ± 0.05**	-0.09 ± 0.08	-0.10 ± 0.07
**7**	**G089227E33891N**	ΔH (m)	-2.01 ± 0.93	-3.92 ± 2.21	+0.62 ± 0.36	-1.79 ± 0.42	+0.05 ± 0.10	-7.86 ± 5.11
		MB (m w.e.a^{-1})	-0.28 ± 0.11	-0.13 ± 0.06	**+0.04 ± 0.02**	-0.25 ± 0.05	**+0.04 ± 0.08**	-0.13 ± 0.07
**8**	**G089189E33837N**	ΔH (m)	-2.18 ± 0.93	-8.25 ± 2.21	-2.08 ± 0.36	-0.89 ± 0.42	+0.09 ± 0.10	-13.65 ± 5.11
		MB (m w.e.a^{-1})	-0.31 ± 0.11	-0.28 ± 0.07	-0.15 ± 0.02	-0.13 ± 0.05	**+0.08 ± 0.08**	-0.23 ± 0.07
**9**	**G089122E33894N**	ΔH (m)	-1.68 ± 0.93	-6.93 ± 2.21	-1.25 ± 0.36	-1.92 ± 0.42	+0.11 ± 0.10	-11.43 ± 5.11
		MB (m w.e.a^{-1})	-0.24 ± 0.11	-0.24 ± 0.07	-0.09 ± 0.02	-0.27 ± 0.05	**+0.09 ± 0.08**	-0.19 ± 0.07
**10**	**G089034E33984N**	ΔH (m)	-1.89 ± 0.93	+0.40 ± 2.21	+1.15 ± 0.36	-0.18 ± 0.42	-0.15 ± 0.10	-0.56 ± 5.11
		MB (m w.e.a^{-1})	-0.27 ± 0.11	**+0.01 ± 0.06**	**+0.08 ± 0.02**	-0.03 ± 0.05	-0.13 ± 0.08	-0.01 ± 0.07
**OVERALL (-2.49 ± 1.48 Gt)**		ΔH (m)	-1.61 ± 0.93	-6.52 ± 2.21	-0.38 ± 0.36	-0.83 ± 0.42	-0.13 ± 0.10	-8.55 ± 5.11
		MB (m w.e.a^{-1})	-0.23 ± 0.11	-0.22 ± 0.07	-0.03 ± 0.02	-0.12 ± 0.05	-0.11 ± 0.08	-0.15 ± 0.07

Supplementary Table 5. Elevation change (ΔH) and mass budget (MB) of selected glaciers and all glaciers in the Western Nyainqentanglha region for the different time periods (**debris cover glacier).

Sl. No.	Glacier	Component	1968-1976	1976-2001	2001-2012	2012-2018	2018-2019	1968-2019
**1**	**G090521E30390N**	ΔH (m)	-3.36 ± 1.23	-15.58 ± 4.56	-8.46 ± 1.64	-4.15 ± 1.08	-0.52 ± 0.23	**-31.82 ± 6.11**
		MB (m w.e.a^{-1})	-0.36 ± 0.11	-0.53 ± 0.13	-0.65 ± 0.11	-0.59 ± 0.15	-0.44 ± 0.18	**-0.53 ± 0.09**
**2**	**G090618E30355N**	ΔH (m)	-2.39 ± 1.23	-8.54 ± 4.56	-4.01 ± 1.64	-2.69 ± 1.08	-0.33 ± 0.23	**-14.87 ± 6.11**
		MB (m w.e.a^{-1})	-0.25 ± 0.11	-0.29 ± 0.13	-0.31 ± 0.11	-0.38 ± 0.15	-0.28 ± 0.18	**-0.25 ± 0.09**
**3****	**G090600E30388N (Xibu)**	ΔH (m)	-2.45 ± 1.23	-5.98 ± 4.56	-4.79 ± 1.64	-3.49 ± 1.08	-0.29 ± 0.23	**-17.07 ± 6.11**
		MB (m w.e.a^{-1})	-0.26 ± 0.11	-0.20 ± 0.13	-0.37 ± 0.11	-0.49 ± 0.15	-0.25 ± 0.18	**-0.28 ± 0.09**
**4**	**G090639E30472N (Zhadang)**	ΔH (m)	-3.83 ± 1.23	-11.31 ± 4.56	-6.93 ± 1.64	-4.55 ± 1.08	-0.53 ± 0.23	**-27.79 ± 6.11**
		MB (m w.e.a^{-1})	-0.41 ± 0.11	-0.38 ± 0.13	-0.54 ± 0.11	-0.64 ± 0.15	-0.45 ± 0.18	**-0.46 ± 0.09**
**5**	**G090550E30416N**	ΔH (m)	-3.43 ± 1.23	-7.34 ± 4.56	-5.73 ± 1.64	-3.66 ± 1.08	-0.48 ± 0.23	**-21.55 ± 6.11**
		MB (m w.e.a^{-1})	-0.36 ± 0.11	-0.25 ± 0.13	-0.44 ± 0.11	-0.48 ± 0.15	-0.41 ± 0.18	**-0.36 ± 0.09**
**OVERALL (-2.73 ± 0.68 Gt)**		ΔH (m)	-2.77 ± 1.23	-7.14 ± 4.56	-5.25 ± 1.64	-3.35 ± 1.08	-0.46 ± 0.23	**-19.23 ± 6.11**
		MB (m w.e.a^{-1})	-0.29 ± 0.11	-0.24 ± 0.13	-0.41 ± 0.11	-0.47 ± 0.15	-0.39 ± 0.18	**-0.32 ± 0.09**

Supplementary Table 6. Elevation change (ΔH) and mass budget (MB) of selected glaciers and all glaciers in the Poiqu region for the different time periods (**debris cover glacier).

Sl. No.	Glacier	Component	1974-2004	2004-2018	2018-2019	1974-2018
1	G085521E28579N	ΔH (m)	-9.31 ± 3.90	-4.93 ± 2.03	-	-13.28 ± 3.96
		MB (m w.e.a^{-1})	-0.26 ± 0.10	-0.30 ± 0.11	-	-0.26 ± 0.07
2	G085618E28467N	ΔH (m)	-9.95 ± 3.90	-4.91 ± 2.03	-	-13.64 ± 3.96
		MB (m w.e.a^{-1})	-0.28 ± 0.10	-0.30 ± 0.11	-	-0.26 ± 0.07
3**	G085694E28431N	ΔH (m)	-14.14 ± 3.90	-9.21 ± 2.03	-	-25.75 ± 3.96
		MB (m w.e.a^{-1})	-0.40 ± 0.10	-0.56 ± 0.11	-	-0.50 ± 0.07
4**	G085751E28386N	ΔH (m)	-8.03 ± 3.90	-4.51 ± 2.03	-	-13.56 ± 3.96
		MB (m w.e.a^{-1})	-0.23 ± 0.10	-0.27 ± 0.11	-	-0.26 ± 0.07
5	G085816E28470N (Kangwure)	ΔH (m)	-11.64 ± 3.90	-10.35 ± 2.03	-0.90 ±0.21	-20.98 ± 3.96
		MB (m w.e.a^{-1})	-0.33 ± 0.10	-0.63 ± 0.11	-0.77 ± 0.16	-0.41 ± 0.07
6**	G085801E28372N	ΔH (m)	-7.98 ± 3.90	-4.81 ± 2.03	-	-11.38 ± 3.96
		MB (m w.e.a^{-1})	-0.23 ± 0.10	-0.29 ± 0.11	-	-0.22 ± 0.07
7	G085812E28335N	ΔH (m)	-12.59 ± 3.90	-4.75 ± 2.03	-	-15.07 ± 3.96
		MB (m w.e.a^{-1})	-0.36 ± 0.10	-0.29 ± 0.11	-	-0.29 ± 0.07
8**	G085751E28316N	ΔH (m)	-11.02 ± 3.90	-8.01 ± 2.03	-	-22.25 ± 3.96
		MB (m w.e.a^{-1})	-0.27 ± 0.10	-0.49 ± 0.11	-	-0.43 ± 0.07
12	G086301E28329N	ΔH (m)	-4.74 ± 3.90	-3.59 ± 2.03	-	-9.52 ± 3.96
		MB (m w.e.a^{-1})	-0.13 ± 0.10	-0.22 ± 0.11	-	-0.18 ± 0.07
13	G086358E28329N	ΔH (m)	-4.05 ± 3.90	-5.44 ± 2.03	-	-10.08 ± 3.96
		MB (m w.e.a^{-1})	-0.11 ± 0.10	-0.33 ± 0.11	-	-0.19 ± 0.07
**Overall (-7.65 ± 1.11 Gt)**		ΔH (m)	-10.73 ± 3.90	-6.84 ± 2.03	-	-18.38 ± 3.96
		MB (m w.e.a^{-1})	-0.30 ± 0.10	-0.42 ± 0.11	-	-0.36 ± 0.07

Supplementary Table 7. Elevation change (ΔH) and mass budget (MB) of selected glaciers and all glaciers in the Langtang region for the different time periods (**debris cover glacier).

Sl. No.	Name of the Glaciers	Mass Balance (m w.e.a^{-1})						
		**1964-1974**	**1974-2004**	**2004-2009**	**2009-2015**	**2015-2017**	**2017-2019**	**1964-2019**
**1********	**G085670E28312N (Langtang)**	-0.25 ± 0.09	-0.28 ± 0.10	-0.49 ± 0.10	-0.61 ± 0.12	-0.74 ± 0.13	-0.86 ± 0.14	**-0.36 ± 0.07**
**2********	**G085747E28200N (Langshisha)**	-0.35 ± 0.09	-0.37 ± 0.10	-0.48 ± 0.10	-0.59 ± 0.12	-0.79 ± 0.13	-0.84 ± 0.14	**-0.46 ± 0.07**
**3********	**G085645E28262N (Shalbachum)**	-0.34 ± 0.09	-0.39 ± 0.10	-0.39 ± 0.10	-0.45 ± 0.12	-0.74 ± 0.13	-0.78 ± 0.14	**-0.42 ± 0.07**
**4********	**G085556E28239N (Lirung)**	-0.21 ± 0.09	-	-	-	-	-	-
**5********	**G085673E28254N (Ghanna)**	-0.37 ± 0.09	-0.41 ± 0.10	-0.45 ± 0.10	-0.45 ± 0.12	-0.67 ± 0.13	-0.66 ± 0.14	**-0.43 ± 0.07**
**6**	**G085573E28258N (Kimoshung)**	-0.06 ± 0.09	-	-	-	-	-	-
**7**	**G085618E28236N (Yala)**	-0.25 ± 0.09	-0.24 ± 0.10	-0.35 ± 0.10	-0.44 ± 0.12	-0.67 ± 0.13	-	**-0.31 ± 0.07**
**OVERALL**		**-0.20 ± 0.09**	**-0.24 ± 0.10**	**-0.37 ± 0.10**	**-0.55 ± 0.12**	**-0.57 ± 0.13**	**-0.59 ± 0.14**	**-0.32 ± 0.07**
		**1964/1974-2004**		**2004-2018/2019**				
**Langtang**		**-0.23 ± 0.10**		**-0.50 ± 0.11**				
**Poiqu**		**-0.30 ± 0.10**		**-0.42 ± 0.11**				

Supplementary Table 8. Elevation change (ΔH) and mass budget (MB) of selected glaciers and all glaciers in the Gurla Mandhata region for the different time periods (**debris cover glacier and bold font indicates positive mass balance).

Sl. No.	Glacier	Component	1966-2000	2000-2011	2011-2013	2013-2016	2016-2018	2018-2019	1966-2019
**1**	**G081387E30495N**	ΔH (m)	-9.58 ± 4.52	-1.64 ± 1.60	-0.54 ± 0.19	-1.01 ± 0.44	-0.45 ± 0.29	-0.28 ± 0.22	-12.69 ± 6.33
		MB (m w.e.a^{-1})	-0.24 ± 0.10	-0.13 ± 0.11	-0.23 ± 0.08	-0.29 ± 0.12	-0.19 ± 0.12	-0.24 ± 0.17	-0.20 ± 0.09
**2**	**G081379E30472N**	ΔH (m)	-9.28 ± 4.52	-2.43 ± 1.60	-0.64 ± 0.19	-0.94 ± 0.44	-0.58 ± 0.29	-0.18 ± 0.22	-14.17 ± 6.33
		MB (m w.e.a^{-1})	-0.23 ± 0.10	-0.19 ± 0.11	-0.27 ± 0.08	-0.27 ± 0.12	-0.25 ± 0.12	-0.15 ± 0.17	-0.23 ± 0.09
**3**	**G081351E30471N (GuNala)**	ΔH (m)	-4.28 ± 4.52	-2.06 ± 1.60	+0.29 ± 0.19	-0.92 ± 0.44	-0.34 ± 0.29	-0.19 ± 0.22	-6.34 ± 6.33
		MB (m w.e.a^{-1})	-0.11 ± 0.10	-0.16 ± 0.11	**+0.12 ± 0.08**	-0.26 ± 0.12	-0.14 ± 0.12	-0.16 ± 0.17	-0.10 ± 0.09
**4**	**G081317E30454N (Gurla)**	ΔH (m)	-9.55 ± 4.52	-3.08 ± 1.60	-0.58 ± 0.19	-0.91 ± 0.44	-0.64 ± 0.29	-0.36 ± 0.22	-15.07 ± 6.33
		MB (m w.e.a^{-1})	-0.24 ± 0.10	-0.24 ± 0.11	-0.25 ± 0.08	-0.26 ± 0.12	-0.27 ± 0.12	-0.31 ± 0.17	-0.24 ± 0.09
**5**	**G081365E30442N**	ΔH (m)	-10.88 ± 4.52	-3.60 ± 1.60	+0.32 ± 0.19	-0.95 ± 0.44	-0.61 ± 0.29	-0.41 ± 0.22	-18.18 ± 6.33
		MB (m w.e.a^{-1})	-0.27 ± 0.10	-0.28 ± 0.11	**+0.14 ± 0.08**	-0.27 ± 0.12	-0.26 ± 0.12	-0.35 ± 0.17	-0.29 ± 0.09
**6**	**G081349E30428N**	ΔH (m)	-8.44 ± 4.52	-2.01 ± 1.60	-0.52 ± 0.19	-0.86 ± 0.44	-0.48 ± 0.29	-0.36 ± 0.22	-14.25 ± 6.33
		MB (m w.e.a^{-1})	-0.21 ± 0.10	-0.16 ± 0.11	-0.22 ± 0.08	-0.24 ± 0.12	-0.20 ± 0.12	-0.31 ± 0.17	-0.23 ± 0.09
**7****	**G081307E30424N (NanManuNaNi)**	ΔH (m)	-9.61 ± 4.52	-1.55 ± 1.60	-0.26 ± 0.19	-0.56 ± 0.44	-0.56 ± 0.29	-0.34 ± 0.22	-13.49 ± 6.33
		MB (m w.e.a^{-1})	-0.24 ± 0.10	-0.12 ± 0.11	-0.11 ± 0.08	-0.16 ± 0.12	-0.24 ± 0.12	-0.29 ± 0.17	-0.22 ± 0.09
**8**	**G081293E30477N**	ΔH (m)	-1.33 ± 4.52	-0.04 ± 1.60	+0.32 ± 0.19	-0.72 ± 0.44	-0.02 ± 0.29	-0.06 ± 0.22	-1.99 ± 6.33
		MB (m w.e.a^{-1})	-0.03 ± 0.10	-0.01 ± 0.11	**+0.14 ± 0.08**	-0.20 ± 0.12	-0.01 ± 0.12	-0.05 ± 0.17	-0.03 ± 0.09
**9**	**G081296E30455N**	ΔH (m)	-0.28 ± 4.52	-1.06 ± 1.60	+0.08 ± 0.19	-0.67 ± 0.44	-0.42 ± 0.29	-0.22 ± 0.22	-1.98 ± 6.33
		MB (m w.e.a^{-1})	-0.01 ± 0.10	-0.08 ± 0.11	**+0.03 ± 0.08**	-0.19 ± 0.12	-0.18 ± 0.12	-0.19 ± 0.17	-0.03 ± 0.09
**10**	**G081267E30456N (NamuNaNi)**	ΔH (m)	+1.42 ± 4.52	-0.51 ± 1.60	-0.09 ± 0.19	-0.27 ± 0.44	-0.14 ± 0.29	-0.13 ± 0.22	-1.57 ± 6.33
		MB (m w.e.a^{-1})	**+0.04 ± 0.10**	-0.04 ± 0.11	-0.04 ± 0.08	-0.08 ± 0.12	-0.06 ± 0.12	-0.11 ± 0.17	-0.04 ± 0.09
**11**	**G081255E30450N**	ΔH (m)	+2.74 ± 4.52	-0.22 ± 1.60	-0.22 ± 0.19	-0.53 ± 0.44	-0.36 ± 0.29	-0.22 ± 0.22	-1.82 ± 6.33
		MB (m w.e.a^{-1})	**+0.07 ± 0.10**	-0.02 ± 0.11	-0.09 ± 0.08	-0.15 ± 0.12	-0.15 ± 0.12	-0.19 ± 0.17	-0.03 ± 0.09
**12**	**G081237E30450N**	ΔH (m)	+3.85 ± 4.52	-0.42 ± 1.60	+0.29 ± 0.19	-0.63 ± 0.44	-0.14 ± 0.29	-0.16 ± 0.22	-4.10 ± 6.33
		MB (m w.e.a^{-1})	**+0.10 ± 0.10**	-0.03 ± 0.11	**+0.12 ± 0.08**	-0.18 ± 0.12	-0.06 ± 0.12	-0.14 ± 0.17	-0.07 ± 0.09
**OVERALL (-0.52 ± 0.36 Gt)**		ΔH (m)	-4.96 ± 4.52	-1.49 ± 1.60	-0.04 ± 0.19	-0.71 ± 0.44	-0.51 ± 0.29	-0.31 ± 0.22	-8.36 ± 6.33
		MB (m w.e.a^{-1})	-0.12 ± 0.10	-0.12 ± 0.11	-0.02 ± 0.08	-0.20 ± 0.12	-0.22 ± 0.12	-0.26± 0.17	-0.13 ± 0.09

Supplementary Table 9. Elevation change (ΔH) and mass budget (MB) of selected glaciers and all glaciers in the Muztagh Ata Massif region for the different time periods (**debris cover glacier and bold font indicates positive mass balance)

Sl. No.	Glacier	Component	1967-1973	1973-2001	2001-2009	2009-2013	2013-2019	1973-2013	1973-2009	1967-2019
1**	G075225E38255 N (Kekesayi)	ΔH (m)	-1.02 ± 0.81	-4.07 ± 2.22	-0.54 ± 1.10	-0.84 ± 0.68	-1.33 ± 0.89	-4.47 ± 4.82	-3.94 ± 3.89	-8.78 ± 5.12
		MB (m w.e.a^{-}	-0.14 ± 0.10	-0.12 ± 0.06	-0.06 ± 0.10	-0.18 ± 0.12	-0.19 ± 0.09	-0.09 ± 0.09	-0.09 ± 0.08	-0.14 ± 0.07
2**	G075233E38272 N	ΔH (m)	-1.83 ± 0.81	-1.25 ± 2.22	-0.85 ± 1.10	-0.18 ± 0.68	-1.78 ± 0.89	-1.33 ± 4.82	-2.98 ± 3.89	-5.21 ± 5.12
		MB (m w.e.a^{-}	-0.26 ± 0.10	-0.04 ± 0.06	-0.09 ± 0.10	-0.04 ± 0.12	-0.25 ± 0.09	-0.03 ± 0.09	-0.07 ± 0.08	-0.09 ± 0.07
3**	G075175E38297 N	ΔH (m)	+1.08 ± 0.81	-5.41 ± 2.22	+0.90 ± 1.10	+0.06 ± 0.68	-0.58 ± 0.89	-3.25 ± 4.82	-4.43 ± 3.89	-2.01 ± 5.12
		MB (m w.e.a^{-}	**+0.15 ± 0.10**	-0.16 ± 0.06	**+0.10 ± 0.10**	**+0.01 ± 0.12**	-0.08 ± 0.09	-0.07 ± 0.09	-0.10 ± 0.08	-0.03 ± 0.07
4	G075079E38288 N (Kematulejia)	ΔH (m)	+0.95 ± 0.81	-2.22 ± 2.22	+0.17 ± 1.10	-0.46 ± 0.68	-0.61 ± 0.89	-0.45 ± 4.82	-1.89 ± 3.89	-2.07 ± 5.12
		MB (m w.e.a^{-}	**+0.13 ± 0.10**	-0.07 ± 0.06	**+0.02 ± 0.10**	-0.10 ± 0.12	-0.09 ± 0.09	-0.01 ± 0.09	-0.04 ± 0.08	-0.03 ± 0.07
5	G075084E38279 N	ΔH (m)	+0.26 ± 0.81	+0.74 ± 2.22	+0.87 ± 1.10	-0.53 ± 0.68	+0.14 ± 0.89	+1.19 ± 4.82	+1.57 ± 3.89	+1.51 ± 5.12
		MB (m w.e.a^{-}	**+0.04 ± 0.10**	**+0.02 ± 0.06**	**+0.09 ± 0.10**	-0.11 ± 0.12	**+0.02 ± 0.09**	**+0.03 ± 0.09**	**+0.04 ± 0.08**	**+0.02 ± 0.07**
6**	G075077E38257 N (Kalaxiong)	ΔH (m)	-0.63 ± 0.81	-1.25 ± 2.22	+1.47 ± 1.10	+0.47 ± 0.68	-0.05 ± 0.89	-0.45 ± 4.82	+1.05 ± 3.89	-1.56 ± 5.12
		MB (m w.e.a^{-}	-0.09 ± 0.10	-0.04 ± 0.06	**+0.16 ± 0.10**	**+0.10 ± 0.12**	-0.01 ± 0.09	-0.01 ± 0.09	**+0.02 ± 0.08**	-0.03 ± 0.07
7	G075058E38248 N (Gl No. 15)	ΔH (m)	+0.23 ± 0.81	+4.37 ± 2.22	+1.63 ± 1.10	+0.75 ± 0.68	+1.09 ± 0.89	+5.68 ± 4.82	+4.74 ± 3.89	+6.76 ± 5.12
		MB (m w.e.a^{-}	**+0.03 ± 0.10**	**+0.13 ± 0.06**	**+0.17 ± 0.10**	**+0.16 ± 0.12**	**+0.15 ± 0.09**	**+0.12 ± 0.09**	**+0.11 ± 0.08**	**+0.11 ± 0.07**
8	G075071E38240 N	ΔH (m)	-0.83 ± 0.81	+0.23 ± 2.22	+0.13 ± 1.10	+0.13 ± 0.68	-1.04 ± 0.89	+1.92 ± 4.82	+0.66 ± 3.89	-0.72 ± 5.12
		MB (m w.e.a^{-}	-0.12 ± 0.10	**+0.01 ± 0.06**	**+0.01 ± 0.10**	**+0.03 ± 0.12**	-0.15 ± 0.09	**+0.04 ± 0.09**	**+0.02 ± 0.08**	-0.01 ± 0.07
9	G075092E38214 N (Kuosikulake)	ΔH (m)	-1.28 ± 0.81	+0.34 ± 2.22	+0.32 ± 1.10	+0.21 ± 0.68	-1.62 ± 0.89	+2.21 ± 4.82	+1.02 ± 3.89	-1.11 ± 5.12
		MB (m w.e.a^{-}	-0.18 ± 0.10	**+0.01 ± 0.06**	**+0.03 ± 0.10**	**+0.04 ± 0.12**	-0.23 ± 0.09	**+0.05 ± 0.09**	**+0.02 ± 0.08**	-0.02 ± 0.07
10	G075075E38189 N	ΔH (m)	-0.12 ± 0.81	+0.65 ± 2.22	+0.56 ± 1.10	+0.21 ± 0.68	-0.28 ± 0.89	+1.19 ± 4.82	+1.10 ± 3.89	-0.21 ± 5.12
		MB (m w.e.a^{-}	-0.02 ± 0.10	**+0.02 ± 0.06**	**+0.06 ± 0.10**	**+0.04 ± 0.12**	-0.04 ± 0.09	**+0.03 ± 0.09**	**+0.03 ± 0.08**	0.00 ± 0.07
11	G075156E38175 N (Kuokuosele)	ΔH (m)	-1.07 ± 0.81	+2.04 ± 2.22	+0.94 ± 1.10	+0.25 ± 0.68	+0.68 ± 0.89	+3.71 ± 4.82	+3.78 ± 3.89	+1.96 ± 5.12
		MB (m w.e.a^{-}	-0.19 ± 0.10	**+0.06 ± 0.06**	**+0.10 ± 0.10**	**+0.05 ± 0.12**	**+0.10 ± 0.09**	**+0.08 ± 0.09**	**+0.09 ± 0.08**	**+0.03 ± 0.07**
12**	G075171E38163 N	ΔH (m)	-1.17 ± 0.81	-1.56 ± 2.22	-0.24 ± 1.10	-0.68 ± 0.68	-0.14 ± 0.89	-3.51 ± 4.82	-1.34 ± 3.89	-4.14 ± 5.12
		MB (m w.e.a^{-}	-0.15 ± 0.10	-0.05 ± 0.06	-0.03 ± 0.10	-0.14 ± 0.12	-0.02 ± 0.09	-0.07 ± 0.09	-0.03 ± 0.08	-0.07 ± 0.07
**OVERALL (-1.01 ± 1.4 Gt)**		ΔH (m)	-0.96 ± 0.81	-0.49 ± 2.22	0.24 ± 1.10	-0.46 ± 0.68	-0.87 ± 0.89	-1.22 ± 4.82	-1.13 ± 3.89	-3.39 ± 5.12
		MB (m w.e.a^{-}	-0.14 ± 0.10	-0.01 ± 0.06	+0.03 ± 0.10	-0.10 ± 0.12	-0.12 ± 0.09	-0.03 ± 0.09	-0.03 ± 0.08	-0.06 ± 0.07

Supplementary Table 10. Area change (ΔA) and length change rate (ΔL) of selected glaciers and all glaciers in the North Tien Shan region for the different time periods.

Sl. No.	Glacier	Component	1964-1971	1971-2000	2000-2012	2012-2016	1971-2012	1971-2016	1964-2020
1	G077081E43044N (Tuyuksu)	ΔA (km^2)	-0.06 ± 0.01	-0.21 ± 0.02	-0.45 ± 0.02	-0.05 ± 0.01	-0.66 ± 0.04	-0.71 ± 0.05	-0.81 ± 0.24
		ΔL (ma^{-1})	-34.23 ± 1.23	-12.56 ± 1.07	-31.34 ± 2.41	-25.45 ± 1.98	-18.06 ± 1.49	-18.71 ± 1.38	-21.93 ± 2.34
2	G077100E43026N	ΔA (km^2)	-0.15 ± 0.01	-0.25 ± 0.01	-0.84 ± 0.03	-0.19 ± 0.01	-1.10 ± 0.07	-1.29 ± 0.07	-1.47 ± 0.48
3	G077112E42985N	ΔA (km^2)	-0.19 ± 0.01	-0.29 ± 0.01	-1.03 ± 0.02	-0.23 ± 0.01	-1.31 ± 0.08	-1.54 ± 0.03	-2.01 ± 0.45
4	G077065E42985N	ΔA (km^2)	-0.08 ± 0.01	-0.04 ± 0.01	-0.27 ± 0.01	-0.07 ± 0.01	-0.31 ± 0.01	-0.37 ± 0.01	-0.49 ± 0.17
5	G077049E42966N	ΔA (km^2)	-0.17 ± 0.01	-0.13 ± 0.01	-0.41 ± 0.02	-0.15 ± 0.02	-0.53 ± 0.02	-0.67 ± 0.03	-0.88 ± 0.31
**OVERALL**		ΔA (km^2)	-2.23 ± 0.11	-1.95 ± 0.10	-9.01 ± 0.46	-2.06 ± 0.11	-10.95 ± 0.54	-13.01 ± 0.66	-15.91 ± 1.9

Supplementary Table 11. Area change (ΔA) and length change rate (ΔL) of selected glaciers and all glaciers in the Ak-Shirak region for the different time periods (++surge type glaciers, ##lake-terminating glaciers and bold font indicates positive length and area change).

Sl. No.	Glacier	Component	1964-1973	1973-1980	1980-2002	2002-2009	2009-2015	2015-2017	2017-2019	1964-2019
**1**	**G078164E41793N (Bordu South)**	ΔA (km^2)	-0.14 ± 0.04	-0.13 ± 0.04	-0.16 ± 0.04	-0.03 ± 0.01	-0.17 ± 0.04	-0.06 ± 0.01	-0.04 ± 0.01	-0.72 ± 0.19
		ΔL (ma^{-1})	-5.84 ± 1.51	-7.04 ± 1.88	-9.10 ± 0.96	-11.17 ± 3.72	-11.59 ± 3.06	-15.31 ± 1.69	-12.57 ± 8.52	-9.19 ± 0.38
**2**	**G078181E41827N (Sary Tor South)**	ΔA (km^2)	-0.03 ± 0.06	-0.13 ± 0.06	-0.19 ± 0.08	-0.09 ± 0.04	-0.16 ± 0.06	-0.05 ± 0.02	-0.04 ± 0.02	-0.69 ± 0.25
		ΔL (ma^{-1})	-0.40 ± 1.51	-7.15 ± 1.88	-11.15 ± 0.96	-35.33 ± 3.72	-19.37 ± 3.06	-9.54 ± 1.69	-13.45 ± 8.52	-12.9 ± 0.38
**3++**	**G078205E41827N (Davidov)**	ΔA (km^2)	**+0.14 ± 0.04**	**+0.15 ± 0.04**	**+0.89 ± 0.25**					
		ΔL (ma^{-1})	**+12.0 ± 1.5**	**+25.5 ± 1.9**	-16.3 ± 0.96					
**4##**	**G078306E41901N (Petrov)**	ΔA (km^2)	-0.55 ± 0.10	-0.59 ± 0.10	-0.33 ± 0.50	-0.25 ± 0.10	-0.22 ± 0.10	-0.10 ± 0.10	-0.13 ± 0.10	-4.16 ± 0.90
		ΔL (ma^{-1})	-23.4 ± 1.51	-31.78 ± 1.88	-39.74 ± 0.96	-21.94 ± 3.72	-13.79 ± 3.06	-5.02 ± 1.69	-4.33 ± 8.52	-28.4 ± 0.38
**5++**	**G078284E41940N (Sary Tor North)**	ΔA (km^2)	-0.04 ± 0.01	-0.05 ± 0.01	-0.52 ± 0.14	-0.13 ± 0.03	-0.26 ± 0.06	-0.04 ± 0.01	-0.05 ± 0.01	-1.08 ± 0.26
		ΔL (ma^{-1})	-0.74 ± 1.51	-14.75 ± 1.88	-30.83 ± 0.96	-31.15 ± 3.72	-45.58 ± 3.06	-106.8 ± 1.69	-123.0 ± 8.52	-31.6 ± 0.38
**6++**	**G078307E41952N (Basimjannij)**	ΔA (km^2)	-0.51 ± 0.02	-0.11 ± 0.05	-0.31 ± 0.13	**+0.26 ± 0.10**	**+0.03 ± 0.01**	-0.13 ± 0.04	**+0.05 ± 0.02**	-0.72 ± 0.27
		ΔL (ma^{-1})	-13.4 ± 1.51	-31.47 ± 1.88	-43.02 ± 0.96	**+18.79 ± 3.72**	**+7.52 ± 3.06**	**+17.46 ± 1.69**	-3.96 ± 8.52	-19.7 ± 0.38
**7**	**G078362E41897N (Dschamansu)**	ΔA (km^2)	-0.02 ± 0.01	-0.11 ± 0.03	-0.13 ± 0.04	-0.41 ± 0.11	-0.21 ± 0.05	-0.11 ± 0.02	-0.08 ± 0.02	-1.05 ± 0.28
		ΔL (ma^{-1})	-3.73 ± 1.51	-2.23 ± 1.88	-17.25 ± 0.96	-48.80 ± 3.72	-40.76 ± 3.06	-41.78 ± 1.69	-39.96 ± 8.52	-21.4 ± 0.38
**8++**	**G078291E41798N (Kaindy)**	ΔA (km^2)	**+0.52 ± 0.14**	**+0.10 ± 0.03**	-0.23 ± 0.06	-1.01 ± 0.28	-0.31 ± 0.08	-0.10 ± 0.02	-0.03 ± 0.01	-1.05 ± 0.28
		ΔL (ma^{-1})	**+38.9 ± 1.51**	-1.51 ± 1.88	-21.98 ± 0.96	-16.86 ± 3.72	-62.86 ± 3.06	-17.70 ± 1.69	-6.58 ± 8.52	-11.4 ± 0.38
**9++**	**G078240E41811N (Kara Say North)**	ΔA (km^2)	-0.32 ± 0.10	-0.16 ± 0.10	-1.65 ± 0.40	-0.57 ± 0.10	-0.48 ± 0.10	-0.09 ± 0.10	-0.06 ± 0.10	-3.34 ± 0.70
		ΔL (ma^{-1})	-23.2 ± 1.51	-5.54 ± 1.88	-29.52 ± 0.96	-12.16 ± 3.72	-31.49 ± 3.06	-24.76 ± 1.69	-45.60 ± 8.52	-23.9 ± 0.38
**Overall**		ΔA (km^2)	**-4.90 ± 1.18**	**-3.63 ± 1.15**	**-11.52 ± 1.45**	**-5.79 ± 1.22**	**-8.74 ± 1.31**	**-2.43 ± 0.81**	**-1.43 ± 0.51**	**-38.4 ± 1.43**

Supplementary Table 12. Area change (ΔA) of selected glaciers and all glaciers in the Purogangri Ice Cap (PIC) region for the different time periods (bold font indicates areal gain).

**Sl. No.**	**Glacier**	**Area Change** (km^2)					
		**1969-1975**	**1975-2000**	**2000-2012**	**2012-2018**	**2018-2019**	**1969-2019**
**1**	**G089071E33998N**	-0.21 ± 0.10	-4.38 ± 0.54	-1.59 ± 0.36	-1.34 ± 0.18	-0.40 ± 0.06	-7.58 ± 1.02
**2**	**G089128E33943N**	-0.53 ± 0.10	-1.23 ± 0.14	-0.86 ± 0.10	-0.87 ± 0.10	-0.20 ± 0.02	-3.68 ± 0.5
**3**	**G089181E33971N**	-0.19 ± 0.10	-0.63 ± 0.09	**+0.05 ± 0.01**	-0.83 ± 0.12	-0.19 ± 0.03	-2.19 ± 0.5
**4**	**G089231E33964N**	**+0.24 ± 0.10**	-0.67 ± 0.10	**+0.26 ± 0.05**	-0.46 ± 0.07	-0.06 ± 0.01	-1.11 ± 0.2
**5**	**G089281E33945N**	-0.17 ± 0.10	-0.44 ± 0.13	-0.18 ± 0.06	-0.26 ± 0.09	0.00 ± 0.01	-1.05 ± 0.34
**6**	**G089272E33936N**	**+0.24 ± 0.10**	-0.91 ± 0.15	-0.35 ± 0.06	-0.16 ± 0.03	0.00 ± 0.01	-1.18 ± 0.21
**7**	**G089227E33891N**	-0.25 ± 0.10	-1.40 ± 0.21	**+1.07 ± 0.21**	-0.33 ± 0.05	0.00 ± 0.01	-1.61 ± 0.27
**8**	**G089189E33837N**	-0.60 ± 0.10	-2.95 ± 0.35	-0.94 ± 0.10	-1.05 ± 0.12	-0.08 ± 0.01	-5.73 ± 0.73
**9**	**G089122E33894N**	-0.11 ± 0.10	-1.88 ± 0.16	-0.31 ± 0.03	-0.80 ± 0.07	-0.07 ± 0.01	-3.87 ± 0.35
**10**	**G089034E33984N**	-0.07 ± 0.10	-1.40 ± 0.20	**+0.53 ± 0.09**	-0.14 ± 0.02	0.00 ± 0.01	-1.88 ± 0.29
**OVERALL**		-1.72 ± 0.10	-15.98 ± 2.04	-1.91 ± 0.17	-6.36 ± 0.10	**+0.09 ± 0.03**	-29.96 ± 4.18

Supplementary Table 13. Area change (ΔA) and Length change rate (ΔL) of selected glaciers and all glaciers in the Western Nyainqentanglha region for the different time periods (**debris cover glacier).

Sl. No.	Glacier	Component	1968-1976	1976-2001	2001-2012	2012-2019	1968-2019
**1**	**G090521E30390N**	ΔA (km^2)	-0.07 ± 0.01	-0.46 ± 0.02	-0.23 ± 0.01	-0.41 ± 0.01	-1.03 ± 0.20
		ΔL (ma^{-1})	-9.79 ± 2.47	-15.14 ± 1.07	-26.32 ± 2.69	-16.54 ± 3.93	-16.90 ± 0.44
**2**	**G090618E30355N**	ΔA (km^2)	-0.08 ± 0.01	-0.55 ± 0.03	-0.05 ± 0.01	-0.04 ± 0.01	-0.62 ± 0.10
		ΔL (ma^{-1})	-6.81 ± 2.47	-30.39 ± 1.07	-4.32 ± 2.69	-5.95± 3.93	-17.71 ± 0.44
**3****	**G090600E30388N (Xibu)**	ΔA (km^2)	-0.14 ± 0.01	-0.25 ± 0.01	-0.17 ± 0.01	-0.13 ± 0.01	-0.69 ± 0.10
		ΔL (ma^{-1})	-23.58 ± 2.47	-10.23 ± 1.07	-15.59 ± 2.69	-5.78 ± 3.93	-12.87 ± 0.44
**4**	**G090639E30472N (Zhadang)**	ΔA (km^2)	-0.09 ± 0.01	-0.31 ± 0.02	-0.08 ± 0.01	-0.19 ± 0.01	-0.67 ± 0.20
		ΔL (ma^{-1})	-15.73 ± 2.47	-7.65 ± 1.07	-9.99 ± 2.69	-17.68 ± 3.93	-10.80 ± 0.44
**5**	**G090550E30416N**	ΔA (km^2)	-0.24 ± 0.02	-0.43 ± 0.02	-0.02 ± 0.01	-0.87 ± 0.02	-1.08 ± 0.20
		ΔL (ma^{-1})	-15.73 ± 2.47	-16.16 ± 1.07	-11.54 ± 2.69	-11.75 ± 3.93	-14.49 ± 0.44
**OVERALL**		ΔA (km^2)	-3.08 ± 0.19	-9.14 ± 0.59	-4.43 ± 0.23	-8.01 ± 0.34	-24.67 ± 1.85

Supplementary Table 14. Area change (ΔA) and Length change rate (ΔL) of selected glaciers and all glaciers in the Poiqu region for the different time periods (**debris cover glacier).

Sl. No.	Glacier	Component	1974-2004	2004-2018	1974-2018
**1**	**G085521E28579N**	ΔA (km^2)	-0.81 ± 0.03	-0.91 ± 0.03	-1.71 ± 0.06
**2**	**G085618E28467N**	ΔA (km^2)	-0.93 ± 0.02	-0.22 ± 0.01	-1.15 ± 0.03
**3****	**G085694E28431N**	ΔA (km^2)	-0.29 ± 0.01	-0.27 ± 0.01	-0.56 ± 0.02
**4****	**G085751E28386N**	ΔA (km^2)	-0.08 ± 0.01	-0.73 ± 0.02	-0.73 ± 0.02
**5**	**G085816E28470N (Kangwure)**	ΔA (km^2)	-0.61 ± 0.03	-0.33 ± 0.02	-0.94 ± 0.04
**6****	**G085801E28372N**	ΔA (km^2)	-0.09 ± 0.02	-0.20 ± 0.01	-0.29 ± 0.01
**7**	**G085812E28335N**	ΔA (km^2)	-0.35 ± 0.07	-7.21 ± 0.14	-7.56 ± 0.17
**8****	**G085751E28316N**	ΔA (km^2)	-0.03 ± 0.01	-0.02 ± 0.01	-0.04 ± 0.01
**9**	**G085670E28312N (Langtang)**	ΔA (km^2)	-0.36 ± 0.02	-0.42 ± 0.02	-0.79 ± 0.03
		ΔL (ma^{-1})	-3.42 ± 1.01	-2.03 ± 2.18	-2.96 ± 0.39
**10**	**G085645E28262N (Shalbachum)**	ΔA (km^2)	-0.19 ± 0.01	-0.07 ± 0.01	-0.18 ± 0.01
		ΔL (ma^{-1})	-14.46 ± 1.01	-9.77 ± 2.18	-12.96 ± 0.39
**11**	**G085747E28200N (langshisha)**	ΔA (km^2)	-0.19 ± 0.01	-0.15 ± 0.01	-0.16 ± 0.01
		ΔL (ma^{-1})	-2.69 ± 1.01	-37.10 ± 2.18	-13.64 ± 0.39
**12****	**G086301E28329N**	ΔA (km^2)	-0.15 ± 0.01	-0.07 ± 0.01	-0.21 ± 0.01
**13****	**G086358E28329N**	ΔA (km^2)	-1.25 ± 0.03	-0.51 ± 0.02	-1.75 ± 0.04
**Overall**		ΔA (km^2)	-17.61 ± 0.76	-21.68 ± 0.72	-39.29 ± 1.52

Supplementary Table 15. Area change (ΔA) and Length change rate (ΔL) of selected glaciers and all glaciers in the Gurla-Mandhata region for the different time periods (**debris cover glacier)

SL. No.	Glacier	Component	1966-2000	2000-2011	2011-2013	2013-2016	2016-2018	2018-2019	1966-2019
**1**	**G081387E30495N**	ΔA (km^2)	-0.15 ± 0.10	-0.05 ± 0.01	-0.01 ± 0.01	-0.00 ± 0.01	+0.00 ± 0.01	-0.01 ± 0.01	-0.21 ± 0.08
**2**	**G081379E30472N**	ΔA (km^2)	-0.07 ± 0.01	-0.04 ± 0.01	-0.00 ± 0.01	-0.00 ± 0.01	-0.01 ± 0.01	-0.01 ± 0.01	-0.13 ± 0.03
**3**	**G081351E30471N (GuNala)**	ΔA (km^2)	-0.03 ± 0.01	-0.05 ± 0.01	-0.01 ± 0.01	-0.01 ± 0.01	+0.00 ± 0.01	-0.01 ± 0.01	-0.09 ± 0.02
**4**	**G081317E30454N (Gurla)**	ΔA (km^2)	-0.24 ± 0.10	-0.08 ± 0.02	-0.01 ± 0.01	-0.01 ± 0.01	-0.00 ± 0.01	-0.00 ± 0.01	-0.33 ± 0.08
		ΔL (ma^{-1})	-10.46 ± 0.82	-9.73 ± 2.97	-10.8 ± 12.4	-4.64 ± 8.23	-0.00 ± 12.56	-0.00 ± 7.83	-9.41 ± 0.33
**5**	**G081365E30442N**	ΔA (km^2)	-0.49 ± 0.10	-0.16 ± 0.03	-0.02 ± 0.01	-0.06 ± 0.01	-0.15 ± 0.03	-0.08 ± 0.02	-0.96 ± 0.17
**6**	**G081349E30428N**	ΔA (km^2)	-0.08 ± 0.01	-0.05 ± 0.02	-0.01 ± 0.01	-0.03 ± 0.01	-0.00 ± 0.01	-0.00 ± 0.01	-0.13 ± 0.05
**7****	**G081307E30424N (NanManuNaNi)**	ΔA (km^2)	-0.03 ± 0.01	+0.00 ± 0.00	-0.00 ± 0.01	-0.01 ± 0.01	-0.00 ± 0.01	-0.01 ± 0.01	-0.04 ± 0.01
**8**	**G081293E30477N**	ΔA (km^2)	-0.04 ± 0.01	-0.02 ± 0.01	-0.01 ± 0.01	-0.00 ± 0.01	-0.00 ± 0.01	-0.00 ± 0.01	-0.06 ± 0.03
**9**	**G081296E30455N**	ΔA (km^2)	-0.13 ± 0.10	-0.02 ± 0.01	-0.00 ± 0.01	-0.00 ± 0.01	-0.00 ± 0.01	-0.01 ± 0.01	-0.16 ± 0.04
**10**	**G081267E30456N (NamuNaNi)**	ΔA (km^2)	-0.02 ± 0.01	-0.01 ± 0.01	-0.01 ± 0.01	-0.01 ± 0.01	+0.00 ± 0.01	-0.01 ± 0.01	-0.05 ± 0.01
**11**	**G081255E30450N**	ΔA (km^2)	-0.03 ± 0.01	-0.02 ± 0.01	-0.00 ± 0.01	-0.01 ± 0.01	+0.00 ± 0.01	-0.01 ± 0.01	-0.06 ± 0.02
**12**	**G081237E30450N**	ΔA (km^2)	-0.01 ± 0.01	-0.01 ± 0.01	-0.01 ± 0.01	-0.01 ± 0.01	+0.00 ± 0.01	-0.00 ± 0.01	-0.02 ± 0.01
**OVERALL**		ΔA (km^2)	-2.51 ± 1.37	-1.26 ± 0.52	-0.22 ± 0.08	-0.08 ± 0.03	-0.14 ± 0.05	-0.14 ± 0.04	-4.34 ± 1.45

Supplementary Table 16. Area, Area change (km^2) & Length change (m) of selected glaciers and all glaciers in the Muztagh Ata Massif region for the different time periods ((**debris cover glacier and bold font indicates positive length change and area gain).

1**	**G075225E38255N (Kekesayi)**	A_{1967}=84.08±1.7; A_{1973}=84.05±1.2; A_{2001}=84.02±0.9; A_{2009}=84.06±0.2; A_{2013}=84.06±0.1; A_{2019}=84.06±0.1					ΔA = -0.03±0.8 km^2
		$L_{1967-1973}$ = -43.17±18	**$L_{1973-2001}$ = 0.00±9**	**$L_{2001-2009}$ = 0.00±16**	**$L_{2009-2013}$ = 0.00±3.6**	**$L_{2013-2019}$ = 0.00±2.2**	$L_{1967-2019}$ = -43.17±5.03 m
2**	**G075233E38272N**	A_{1967}=18.91±0.5; A_{1973}=18.9±0.5; A_{2001}=18.9±0.4; A_{2009}=18.9±0.1; A_{2013}=18.8±0.1; A_{2019}=18.7±0.1					ΔA = -0.13±0.8 km^2
		**$L_{1967-1973}$ = 0.00±18**	$L_{1973-2001}$ = -32.0±9	$L_{2001-2009}$ = -145.3±16	$L_{2009-2013}$ = -42.2±3.6	$L_{2013-2019}$ = -25.4±2.2	$L_{1967-2019}$ = -240.3±5.03 m
3**	**G075175E38297N**	A_{1967}=8.87±0.2; A_{1973}=8.86±0.2; A_{2001}=8.70±0.1; A_{2009}=8.67±0.1; A_{2013}=8.68±0.1; A_{2019}=8.66±0.1					ΔA = -0.22±0.8 km^2
		$L_{1967-1973}$ = -27.8±18	$L_{1973-2001}$ = -393±9	$L_{2001-2009}$ = -41.4±16	**$L_{2009-2013}$ = +20.7±3.6**	$L_{2013-2019}$ = -34.2±2.2	$L_{1967-2019}$ = -444.4±5.03 m
4	**G075079E38288N (Kematulejia)**	A_{1967}=11.86±0.3; A_{1973}=11.88±0.3; A_{2001}=11.70±0.2; A_{2009}=11.75±0.1; A_{2013}=11.87±0.1; A_{2019}=11.79±0.1					ΔA = -0.08±0.8 km^2
		**$L_{1967-1973}$ = +49.3±18**	$L_{1973-2001}$ = -226±9	**$L_{2001-2009}$ = 0.00±16**	**$L_{2009-2013}$ = +10.3±3.6**	**$L_{2013-2019}$ = +44.8±2.2**	$L_{1967-2019}$ = -122.4±5.03 m
5	**G075084E38279N**	A_{1967}=15.20±0.3; A_{1973}=15.12±0.3; A_{2001}=15.05±0.2; A_{2009}=15.06±0.1; A_{2013}=15.07±0.1; A_{2019}=15.06±0.1					ΔA = -0.15±0.8 km^2
		$L_{1967-1973}$ = -106.8±18	$L_{1973-2001}$ = -135±9	**$L_{2001-2009}$ = 0.00±16**	**$L_{2009-2013}$ = +20.8±3.6**	**$L_{2013-2019}$ = 0.00±2.2**	$L_{1967-2019}$ = -232.3±5.03 m
6**	**G075077E38257N (Kalaxiong)**	A_{1967}=23.90±0.3; A_{1973}=23.75±0.3; A_{2001}=23.71±0.2; A_{2009}=23.75±0.1; A_{2013}=23.75±0.1; A_{2019}=23.67±0.1					ΔA = -0.23±0.8 km^2
		$L_{1967-1973}$ = -231.8±18	**$L_{1973-2001}$ = 0.00±9**	**$L_{2001-2009}$ = +18.5±16**	**$L_{2009-2013}$ = 0.00±3.6**	**$L_{2013-2019}$ = 0.00±2.2**	$L_{1967-2019}$ = -227.9±5.03 m
7	**G075058E38248N (Gl No. 15)**	A_{1967} =0.91±0.1; A_{1973}=0.90±0.01; A_{2001}=0.91±0.1; A_{2009}=0.91±0.1; A_{2013}=0.91±0.1; A_{2019}=0.91±0.1					ΔA = -0.00±0.8 km^2
		**$L_{1967-1973}$ = 0.00±18**	**$L_{1973-2001}$ = 0.00±9**	**$L_{2001-2009}$ = 0.00±16**	**$L_{2009-2013}$ = 0.00±3.6**	**$L_{2013-2019}$ = 0.00±2.2**	**$L_{1967-2019}$ = 0.00±5.03 m**
8	**G075071E38240N**	A_{1967}=9.08±0.2; A_{1973}=9.04±0.2; A_{2001}=8.95±0.2; A_{2009}=8.93±0.1; A_{2013}=8.93±0.1; A_{2019}=8.92±0.1					ΔA = -0.16±0.8 km^2
		$L_{1967-1973}$ = -62.1±18	$L_{1973-2001}$ = -128±9	$L_{2001-2009}$ = -43.4±16	**$L_{2009-2013}$ = 0.00±3.6**	$L_{2013-2019}$ = -17.1±2.2	$L_{1967-2019}$ = -248.3±5.03 m
9	**G075092E38214N (Kuosikulake)**	A_{1967} =21.27±0.4; A_{1973}=21.3±0.4; A_{2001}=21.2±0.3; A_{2009}=21.02±0.1; A_{2013}=21.15±0.1; A_{2019}=21.04±0.5					ΔA = -0.23±0.8 km^2
		**$L_{1967-1973}$ = +88.3±18**	$L_{1973-2001}$ = -42.4±9	$L_{2001-2009}$ = -369.7±16	**$L_{2009-2013}$ = +252.4±3.6**	**$L_{2013-2019}$ = +471.2±2.2**	**$L_{1967-2019}$ = +416.7±5.03 m**
10	**G075075E38189N**	A_{1967}=3.05±0.1; A_{1973}=3.02±0.1; A_{2001}=2.9±0.1; A_{2009}=3.12±0.1; A_{2013}=3.17±0.1; A_{2019}=3.12±0.1					**ΔA = +0.07±0.01 km^2**
		$L_{1967-1973}$ = -80.9±18	$L_{1973-2001}$ = -133±9	**$L_{2001-2009}$ = +135.2±16**	**$L_{2009-2013}$ = +61.7±3.6**	$L_{2013-2019}$ = -26.2±2.2	$L_{1967-2019}$ = -39.6±5.03 m
11	**G075156E38175N (Kuokuosele)**	A_{1967}=22.92±0.4; A_{1973}=22.90±0.4; A_{2001}=23.1±0.3; A_{2009}=23.2±0.1; A_{2013}=23.3±0.1; A_{2019}=23.4±0.1					**ΔA = +0.47±0.8 km^2**
		$L_{1967-1973}$ = -37.6±18	**$L_{1973-2001}$ = +181±9**	**$L_{2001-2009}$ = +338.3±16**	**$L_{2009-2013}$ = +136.9±3.6**	**$L_{2013-2019}$ =+194.9±2.2**	**$L_{1967-2019}$ = +803.8±5.03** m
12**	**G075171E38163N**	A_{1967}=8.51±0.3; A_{1973}=8.50±0.3; A_{2001}=8.52±0.2; A_{2009}=8.52±0.1; A_{2013}=8.52±0.1; A_{2019}=8.51±0.1					ΔA = 0.00±0.8 km^2
		$L_{1967-1973}$ = -37.2±18	**$L_{1973-2001}$ = +54.3±9**	**$L_{2001-2009}$ = 0.00±16**	**$L_{2009-2013}$ = 0.00±3.6**	**$L_{2013-2019}$ = 0.00±2.2**	**$L_{1967-2019}$ = +20.8±5.03 m**
**ALL**		A_{1967}=347.5±9.2; A_{1973}=347.1±9.1; A_{2001}=346.4±6.8; A_{2009}=346.6±1.9; A_{2013}=346.8±0.9; A_{2019}=343.7±4.1					ΔA = -3.84±0.8 km^2

Supplementary Table 17. Summer, winter and annual temperature, precipitation and annual solid precipitation for different time periods based on ERA5 Land Climate data.

Region	Year	Avg. Summer temp (°C)	Avg. Winter temp (°C)	Avg. Annual temp (°C)	Summer Precipitation (mm/a)	Winter Precipitation (mm/a)	Annual Precipitation (mm/a)	Solid Precipitation (mm/a)
North Tien Shan	1971-2000	3.94	-9.79	-2.92	901	408	1309	551
	2000-2012	4.32	-9.29	-2.48	912	434	1347	531
	2012-2016	4.49	-9.08	2.29	889	438	1326	569
	2016-2020	4.51	-8.87	2.28	932	485	1402	519
Ak Shirak	1980-2002	-2.32	-17.59	-9.95	467	148	616	374
	2002-2009	-2.49	-17.08	-9.74	470	148	618	387
	2009-2015	-1.98	-17.61	-9.79	448	137	586	391
	2015-2017	-1.96	-16.79	-9.38	449	182	631	399
	2017-2019	-2.11	-16.81	-9.46	443	140	584	367
Purogangri Ice Cap	1975-2000	-2.65	-16.84	-9.75	461	81	541	272
	2000-2012	-2.89	-16.04	-9.47	516	80	596	314
	2012-2018	-2.25	-15.62	-8.94	519	81	600	284
	2018-2019	-	-	-	-	-	-	
Western Nyainqentanglha	1976-2001	1.97	-10.63	-4.12	742	96	838	234
	2001-2012	2.20	-9.86	-4.06	785	100	885	209
	2012-2018	2.65	-9.19	-3.07	764	97	861	210

	2018-2019	-	-	-	-	-	-	
**Poiqu region**	1974-2004	3.25	-8.51	-2.64	1328	343	1671	441
	2004-2018	3.42	-7.64	-2.10	1437	309	1746	382
**Langtang**	1974-2004	-1.3	-13.1	-6.2	1295	259	1554	514.2
	2004-2009	-1.5	-12.3	-6.0	1325	205	1530	480.3
	2009-2015	-1.5	-12.9	-6.3	1380	260	1639	481.7
	2015-2017	-1.1	-12.1	-5.7	1362	248	1610	476.7
	2017-2019	-1.2	-12.6	-5.9	1429	260	1690	460.8
**Gurla Mandhata**	1966-2000	-0.24	-16.45	-8.36	3219	1715	4934	2286
	2000-2011	-0.22	-15.64	-7.92	3742	1501	5243	2242
	2011-2013	0.04	-16.48	-8.23	4101	1704	5805	1964
	2013-2016	0.15	-15.44	-7.64	3211	1636	4847	2371
	2016-2018	0.82	-14.91	-7.54	4084	1184	5269	1568
	2018-2019	-	-	-	-	-	-	
**Muztagh Ata Massif**	1973-2001	-0.99	-17.00	-9.01	321	106	427	212
	2001-2009	-0.95	-16.92	-8.93	297	108	405	224
	2009-2013	-0.60	-17.34	-8.97	308	101	410	235
	2013-2019	-0.39	-16.53	-8.46	307	111	419	225

Supplementary Table 18. Characteristics of the available and analyzed weather stations for the seven study regions

Sl. No.	Station Name	Location	Latitude/Longitude	Station Elevation (m a.s.l)	Avg. Elevation of ERA5 Land Data (m a.s.l)	Approximate ELA (m a.s.l)	Data Availability
1	Tuyuksu	IleAlatau, Northern Tien Shan	43.07°N 78.08°E	3434	2800	3800-4000	1972-2020
2	Tien Shan	Arabel-Syrt, close to Ak-Shirak, Central Tien Shan	41.88°N 78.17°E	3600	4210	4200-4300	1930-2013
3	Bange	Purogangri Ice Cap, (300 km)	31.48^0 N 89.40^0 E	4700	5431	5748	1957-2017
4	Dangxiong	Western Nyainqentanglha	30.29^0 N 91.05^0 E	4200	5095	5770	1963-2017
5	Nielamu	Poiqu region	28.11^0 N 85.58^0 E	3810	4970	5600	1967-2017
6	Pulan	Gurla Mandhata	30.17^0 N 81.15^0 E	3900	5019	5500-5600	1973-2017
7	Tashikuergen	50 km South of Muztagh-Ata Massif	37.46^0 N 75.14^0 E	3091	4481	5285	1957-2017

Supplementary Table 19. Correlation matrix (r^2 and p- value) between the High Asia Refined Analysis (HAR V2) water vapor and precipitation data with ERA5 Land and corresponding weather station data for Northern Tien Shan, Gurla Mandhata & Muztagh-Ata region

Region	HAR V2	ERA5 Land				Weather Station		
		Summer precipitation	Winter precipitation	Annual precipitation	Solid precipitation	Summer precipitation	Winter precipitation	Annual precipitation
Northern Tien Shan	Water vapour (summer)	**0.44 & 0.1**				**0.44 & 0.2**		
	Water vapour (winter)		**0.55 & 0.1**				**0.18 & 0.3**	
	Water vapour (annual)			**0.58 & 0.02**				**0.37 & 0.2**
	Precipitation (summer)					**0.54 & 0.04**		
	Precipitation (winter)		**0.86 & 0.001**				**0.54 & 0.001**	
	Precipitation (annual)			**0.60 & 0.004**				**0.75 & 0.02**
	Precipitation (solid)				**0.69 & 0.01**			
Gurla Mandhata	Water vapour (summer)	**0.32 & 0.4**				**0.06 & 0.2**		
	Water vapour (winter)		**0.01 & 0.8**				**0.13 & 0.5**	
	Water vapour (annual)			**0.05 & 0.1**				**0.03 & 0.2**
	Precipitation (summer)					**0.02 & 0.4**		
	Precipitation (winter)		**0.84 & 0.01**				**0.54 & 0.06**	
	Precipitation (annual)			**0.63 & 0.01**				**0.05 & 0.5**
	Precipitation (solid)				**0.78 & 0.02**			
Muztagh Ata	Water vapour (summer)	**0.10 & 0.1**				**0.01 & 0.5**		
	Water vapour (winter)		**0.18 & 0.2**				**0.01 & 0.5**	
	Water vapour (annual)			**0.15 & 0.2**				**0.01 & 0.1**
	Precipitation (summer)					**0.12 & 0.1**		
	Precipitation (winter)		**0.85 & 0.004**				**0.08 & 0.1**	
	Precipitation (annual)			**0.81 & 0.01**				**0.43 & 0.04**
	Precipitation (solid)				**0.89 & 0.005**			

Supplementary Table 20. Comparison of glacier mass balances with other available studies and measurements in the Northern Tien Shan region.

Name & GLIMS ID	Year	MB (m w.e.a^{-1})	Area (km^2)	Study	Method
**Overall North Tien Shan**	1964-1971	-0.18 ± 0.11	50.37	This Study	Geodetic
	1971-2000	-0.32 ± 0.10			
	2000-2012	-0.43 ± 0.09			
	2012-2016	-0.45 ± 0.13			
	2016-2020	-0.49 ± 0.13			
	1964-2020	-0.40 ± 0.09			
	2000-2016	-0.41 ± 0.20		Brun et al. (2017)[33]	
**1. G077081E43044N (Tuyuksu Glacier)**	1958-1998	-0.42	-	Hagg et al. (2004) [47]	Glaciological
	1958-1998	-0.32			Map
	1964-2000	-0.33 ± 0.09	2.84	This Study	Geodetic
	2000-2012	-0.38 ± 0.09			
	2012-2016	-0.44 ± 0.12			
	2000-2016	-0.39 ± 0.10			
	2016-2020	-0.48 ± 0.13			
	1964-2016	-0.42 ± 0.08			
	1964-2020	-0.43 ± 0.09			
	1971-2012	-0.35 ± 0.07			
	1971-2016	-0.34 ± 0.07			
	2000-2016	-0.40 ± 0.20		Brun et al. (2017) [33]	
	1964-2016	-0.43		WGMS[48]	Glaciological
	1964-2000	-0.43			
	2000-2012	-0.40			
	2012-2016	-0.53			
	1971-2012	-0.51			
	1971-2016	-0.50			
	1957-2018	-0.40			
	1958-2016	-0.35 ± 0.18		Kapista et al. (2020)[49]	Glaciological+ geodetic
**2. G077100E43026N**	1964-2000	-0.19 ± 0.09	2.9	This Study	Geodetic
	2000-2012	-0.52 ± 0.10			
	2012-2016	-0.39 ± 0.13			
	2016-2020	-0.39 ± 0.13			
	2000-2016	-0.48 ± 0.10			

	1964-2020	-0.36 ± 0.09			
	2000-2016	-0.46 ± 0.20		Brun et al. (2017) [33]	
**3. G077112E42985N**	1964-2000	-0.31 ± 0.09	8.4	This Study	Geodetic
	2000-2012	-0.46 ± 0.09			
	2012-2016	-0.47 ± 0.13			
	2016-2020	-0.55 ± 0.13			
	2000-2016	-0.47 ± 0.10			
	1964-2020	-0.45 ± 0.09			
	2000-2016	-0.48 ± 0.20		Brun et al. (2017) [33]	
**4. G077065E42985N**	1964-2000	-0.30 ± 0.09	2.4	This Study	Geodetic
	2000-2012	-0.43 ± 0.09			
	2012-2016	-0.36 ± 0.12			
	2016-2020	-0.47 ± 0.13			
	2000-2016	-0.45 ± 0.10			
	1964-2020	-0.39 ± 0.09			
	2000-2016	-0.51 ± 0.20		Brun et al. (2017) [33]	
**5. G077049E42966N**	1964-2000	-0.28 ± 0.09	3.2	This Study	Geodetic
	2000-2012	-0.46 ± 0.09			
	2012-2016	-0.49 ± 0.13			
	2016-2020	-0.49 ± 0.13			
	2000-2016	-0.47 ± 0.10			
	1964-2020	-0.45 ± 0.09			
	2000-2016	-0.45 ± 0.20		Brun et al. (2017) [33]	

Supplementary Table 21. Comparison of glacier mass balances with other available studies and measurements in the Ak-Shirak region

Name & GLIMS ID	Year	MB (m w.e.a^{-1})	Area (km^2)	Study	Method
**Overall Ak-Shirak**	1964-1980	-0.28 ± 0.16	364.8	This study	Geodetic
	1973-2002	-0.47 ± 0.12			
	1980-2002	-0.54 ± 0.10			
	2002-2017	-0.31 ± 0.14			
	2017-2019	-0.29 ± 0.23			
	1964-2019	-0.40 ± 0.07			
	1964-1980	-0.40 ± 0.10	-	Goerlich et al. (2017)[7]	
	1975-1999	-0.51 ± 0.36	-	Pieczonka & Bolch (2015)[8]	
	1977-1999	-0.59 ± 0.31	-	Aizen et al. (2007)[50]	
	2000-2016	-0.32 ± 0.20	364.8	Brun et al. (2017) [33]	
**1. G078164E41793N (Bordu South (354)**	1964-1980	-0.27 ± 0.10	6.54	This study	Geodetic
	1980-2002	-0.54 ± 0.10			
	2002-2017	-0.45 ± 0.14			
	1964-2019	-0.43 ± 0.07			
	1964-1980	-0.30 ± 0.10	-	Goerlich et al. (2017)[7]	
	2000-2016	-0.43 ± 0.20	6.54	Brun et al. (2017) [33]	
	2003-2012	-0.43 ± 0.09	-	Kronenberg et al. (2015)[22]	Model based
**2. G078181E41827N (Sary Tor South)**	1964-1980	-0.27 ± 0.10	2.93	This study	Geodetic
	1980-2002	-0.53 ± 0.10			
	2002-2017	-0.36 ± 0.14			
	1964-2019	-0.46 ± 0.07			
	1964-1980	-0.30 ± 0.40	-	Goerlich et al. (2017)[7]	
	2000-2016	-0.34 ± 0.20	2.93	Brun et al. (2017) [33]	
	1985-1989	-0.14	-	WGMS4[8]	Glaciological
	1943-1977	-0.48	-	Petrakov et al. (2014)[51]	Model based
**4. G078306E41901N (Petrov)**	1964-1980	-0.50 ± 0.10	62.2	This study	Geodetic
	1980-2002	-0.68 ± 0.10			
	2002-2017	-0.34 ± 0.14			
	1964-2019	-0.68 ± 0.07			
	1964-1980	-0.60 ± 0.10	-	Goerlich et al. (2017)[7]	
	2000-2016	-0.31 ± 0.20	62.2	Brun et al. (2017) [33]	
**5. G078284E41940N (Sary Tor North)**	1964-1980	-0.28 ± 0.10	8.59	This study	Geodetic
	1980-2002	-0.59 ± 0.10			

	2002-2017	-0.26 ± 0.13			
	1964-2019	-0.49 ± 0.07			
	1964-1980	-0.40 ± 0.20	-	Goerlich et al. (2017)[7]	
	2000-2016	-0.21 ± 0.20	8.59	Brun et al. (2017) [33]	
**6. G078307E41952N (Basimjannij)**	1964-1980	-0.53 ± 0.11	4.49	This study	Geodetic
	1980-2002	-0.57 ± 0.10			
	2002-2017	-0.56 ± 0.14			
	1964-2019	-0.59 ± 0.07			
	1964-1980	-0.50 ± 0.20	-	Goerlich et al. (2017)[7]	
	2000-2016	-0.52 ± 0.20	4.49	Brun et al. (2017) [33]	
**7. G078362E41897N (Dschamansu)**	1964-1980	-0.49 ± 0.10	23.17	This study	Geodetic
	1980-2002	-0.50 ± 0.11			
	2002-2017	-0.34 ± 0.14			
	1964-2019	-0.51 ± 0.07			
	1964-1980	-0.70 ± 0.20	-	Goerlich et al. (2017)[7]	
	2000-2016	-0.32 ± 0.20	23.17	Brun et al. (2017) [33]	
**8. G078291E41798N (Kaindy)**	1964-1980	-0.29 ± 0.10	18.83	This study	Geodetic
	1980-2002	-0.66 ± 0.10			
	2002-2017	-0.34 ± 0.14			
	1964-2019	-0.48 ± 0.07			
	1964-1980	-0.30 ± 0.30	-	Goerlich et al. (2017)[7]	
	2000-2016	-0.41 ± 0.20	18.83	Brun et al. (2017) [33]	
**10. G078240E41811N (Kara Say North)**	1964-1980	-0.55 ± 0.11	33.49	This study	Geodetic
	1980-2002	-0.56 ± 0.10			
	2002-2017	-0.50 ± 0.14			
	1964-2019	-0.59 ± 0.07			
	1964-1980	-0.60 ± 0.30	-	Goerlich et al. (2017)[7]	
	2000-2016	-0.53 ± 0.20	33.49	Brun et al. (2017) [33]	

Supplementary Table 22. Comparison of glacier mass balances with other available studies and measurements in the Purogangri Ice Cap (PIC) region.

Name & GLIMS ID	Year	MB (m w.e.a^{-1})	Area (km^2)	Study	Method
Overall Purogangri Ice Cap	1969-1975	-0.23 ± 0.11	344	This Study	Geodetic
	1975-2000	-0.22 ± 0.07			
	2000-2012	-0.03 ± 0.02			
	2012-2018	-0.12 ± 0.05			
	1969-2019	-0.15 ± 0.07			
	2000-2016	-0.07 ± 0.05		Brun et al. (2017) [33]	Geodetic
	1974-2000	-0.21 ± 0.07	398.5	Lei et al. (2012)[52]	Topographic maps
	2001-2011	-0.044	420.0	Huintjes et al. (2015)[53]	Model-based
	2000-2011	-0.05 ± 0.08	420.0	Zhang et al. (2018)[25]	Albedo-based
	2012-2015	-0.29 ± 0.13			
	2000-2016	-0.11 ± 0.07			
	2012-2016	-0.27 ± 0.03	420.0	Liu et al. (2016)[54]	Geodetic
	2000-2012	-0.04 ± 0.02	408.0	Neckel et al. (2013)[37]	Geodetic
1. G089071E33998N	1969-1975	-0.28 ± 0.11	46.9	This Study	Geodetic
	1975-2000	-0.29 ± 0.07			
	2000-2012	-0.03 ± 0.02			
	2000-2018	-0.05 ± 0.03			
	1969-2019	-0.16 ± 0.07			
	2000-2012	-0.02 ± 0.02		Neckel et al. (2013)[37]	
	2000-2016	-0.10 ± 0.07		Brun et al. (2017) [33]	
2. G089128E33943N	1969-1975	+0.02 ± 0.11	56.7	This Study	Geodetic
	1975-2000	-0.13 ± 0.07			
	2000-2012	+0.03 ± 0.02			
	2000-2018	+0.02 ± 0.03			
	1969-2019	+0.01 ± 0.07			
	2000-2012	+0.03 ± 0.02		Neckel et al. (2013)[37]	
	2000-2016	-0.02 ± 0.07		Brun et al. (2017) [33]	
3. G089181E33971N	1969-1975	-0.18 ± 0.11	30.8	This Study	Geodetic

	1975-2000	-0.30 ± 0.07			
	2000-2012	+0.03 ± 0.02			
	2000-2018	-0.02 ± 0.03			
	1969-2019	-0.16 ± 0.07			
	2000-2012	+0.04 ± 0.02		Neckel et al. (2013)[37]	
	2000-2016	-0.07 ± 0.07		Brun et al. (2017) [33]	
**4. G089231E33964N**	1969-1975	-0.25 ± 0.11	16.8	This Study	Geodetic
	1975-2000	-0.22 ± 0.07			
	2000-2012	+0.05 ± 0.02			
	2000-2018	-0.02 ± 0.03			
	1969-2019	-0.16 ± 0.07			
	2000-2012	+0.06 ± 0.02		Neckel et al. (2013)[37]	
	2000-2016	-0.04 ± 0.07		Brun et al. (2017) [33]	
**5. G089281E33945N**	1969-1975	-0.27 ± 0.11	4.5	This Study	Geodetic
	1975-2000	-0.27 ± 0.07			
	2000-2012	-0.01 ± 0.02			
	2000-2018	-0.08 ± 0.03			
	1969-2019	-0.18 ± 0.07			
	2000-2012	-0.01 ± 0.02		Neckel et al. (2013)[37]	
	2000-2016	-0.09 ± 0.07		Brun et al. (2017) [33]	
**6. G089272E33936N**	1969-1975	-0.25 ± 0.11	12.7	This Study	Geodetic
	1975-2000	-0.23 ± 0.07			
	2000-2012	+0.11 ± 0.02			
	2000-2018	+0.09 ± 0.03			
	1969-2019	-0.10 ± 0.07			
	2000-2012	+0.09 ± 0.02		Neckel et al. (2013)[37]	
	2000-2016	+0.08 ± 0.07		Brun et al. (2017) [33]	
**7. G089227E33891N**	1969-1975	-0.28 ± 0.11	24.8	This Study	Geodetic
	1975-2000	-0.13 ± 0.07			
	2000-2012	+0.04 ± 0.02			
	2000-2018	-0.06 ± 0.03			
	1969-2019	-0.13 ± 0.07			

	2000-2012	+0.02 ± 0.02		Neckel et al. (2013)[37]	
	2000-2016	-0.04 ± 0.07		Brun et al. (2017) [33]	
**8. G089189E33837N**	1969-1975	-0.31 ± 0.11	39.9	This Study	Geodetic
	1975-2000	-0.28 ± 0.07			
	2000-2012	-0.15 ± 0.02			
	2000-2018	-0.14 ± 0.03			
	1969-2019	-0.23 ± 0.07			
	2000-2012	-0.17 ± 0.02		Neckel et al. (2013)[37]	
	2000-2016	-0.11 ± 0.07		Brun et al. (2017) [33]	
**9. G089122E33894N**	1969-1975	-0.24 ± 0.11	57.1	This Study	Geodetic
	1975-2000	-0.24 ± 0.07			
	2000-2012	-0.09 ± 0.02			
	2000-2018	-0.14 ± 0.03			
	1969-2019	-0.19 ± 0.07			
	2000-2012	-0.08 ± 0.02		Neckel et al. (2013)[37]	
	2000-2016	-0.15 ± 0.07		Brun et al. (2017) [33]	
**10. G089034E33984N**	1969-1975	-0.27 ± 0.11	33.0	This Study	Geodetic
	1975-2000	+0.01 ± 0.06			
	2000-2012	+0.08 ± 0.02			
	2000-2018	+0.05 ± 0.03			
	1969-2019	-0.01 ± 0.07			
	2000-2012	+0.07 ± 0.02		Neckel et al. (2013)[37]	
	2000-2016	+0.08 ± 0.07		Brun et al. (2017) [33]	

Supplementary Table 23. Comparison of glacier mass balances with other available studies and measurements in the Western Nyainqentanglha region.

Name & GLIMS ID	Year	MB (m w.e.a^{-1})	Area (km^2)	Study	Method
Overall region	1968-1976	-0.29 ± 0.11	167.3	This study	Geodetic
	1976-2001	-0.24 ± 0.13			
	2001-2012	-0.41 ± 0.11			
	2012-2018	-0.47 ± 0.15			
	2018-2019	-0.39 ± 0.18			
	1968-2019	-0.32 ± 0.09			
	1966-2013	-0.67 ± 0.54	-	Kang et al. (2015)[56]	Ice core tritium and mercury records
	1976-2000	-0.25 ± 0.15	316.8	Zhou et al. (2018) [38]	Geodetic
	1999-2010	-0.34 ± 0.12	-	Gardelle et al. (2013)[57]	Geodetic
	2000-2014	-0.30 ± 0.07	-	Zhang & Zhang (2017)[58]	Geodetic
	2000-2014	-0.24 ± 0.13	-	Li & Lin (2017)[55]	Geodetic
	2000-2016	-0.42 ± 0.23	-	Brun et al. (2017)[33]	Geodetic
	2002-2009	-1.19 ± 0.09	-	Wu et al. (2014)[39]	ICESat
	2003-2008	-0.32 ± 0.21	-	Gardner et al. (2013)[59]	Geodetic
	2003-2009	-0.20 ± 0.29	-	Neckel et al. (2014)[53]	ICESat (Only accumulation)
1. G090521E30390N	1968-1976	-0.36 ± 0.11	11.5	This study	Geodetic
	1976-2001	-0.53 ± 0.14			
	2001-2012	-0.65 ± 0.11			
	2012-2018	-0.59 ± 0.15			
	1968-2019	-0.53 ± 0.09			
	2000-2016	-0.64 ± 0.23		Brun et al. (2017) [33]	
2. G090618E30355N	1968-1976	-0.25 ± 0.11	7.3	This study	Geodetic
	1976-2001	-0.29 ± 0.13			
	2001-2012	-0.31 ± 0.11			
	2012-2018	-0.38 ± 0.15			
	1968-2019	-0.25 ± 0.09			
	2000-2016	-0.31 ± 0.23		Brun et al. (2017) [33]	
3. G090600E30388N (Xibu Glacier)	1968-1976	-0.26 ± 0.11	27.5	This study	Geodetic
	1976-2001	-0.20 ± 0.13			

	2001-2012	-0.37 ± 0.11			
	2012-2018	-0.49 ± 0.15			
	1968-2019	-0.28 ± 0.09			
	2000-2016	-0.47 ± 0.23		Brun et al. (2017) [33]	
**4. G090639E30472N (Zhadang Glacier)**	1968-1976	-0.41 ± 0.11	1.6	This study	Geodetic
	1976-2001	-0.38 ± 0.13			
	2001-2012	-0.54 ± 0.11			
	2012-2018	-0.64 ± 0.15			
	1968-2019	-0.46 ± 0.09			
	2000-2016	-0.61 ± 0.23		Brun et al. (2017) [33]	
	2000-2014	-0.50 ± 0.17	-	Li et al. (2017)	Geodetic
	2000-2014	-0.61 ± 0.07	-	Zhang & Zhang (2017)[58]	Geodetic
	2001-2011	-1.10	-	Huintjes et al. (2015)[53]	Model based
	2005-2008	-0.59	-	Yu et al. (2013)[62]	Glaciological
	2006-2008	-0.55	-	Kang et al. (2009)[56]	Glaciological
	2010-2011	-0.41	-	Zhang et al. (2013)[61]	Model based
	2010-2012	-1.82	-	Zhu et al. (2015)[60]	Model based
**5. G090550E30416N**	1968-1976	-0.36 ± 0.11	7.6	This study	Geodetic
	1976-2001	-0.25 ± 0.13			
	2001-2012	-0.44 ± 0.11			
	2012-2018	-0.48 ± 0.15			
	1968-2019	-0.36 ± 0.09			
	2000-2016	-0.45 ± 0.23		Brun et al. (2017) [33]	

Supplementary Table 24. Comparison of glacier mass balances with other available studies and measurements in the Poiqu region.

Name & GLIMS ID	Year	MB (m w.e.a^{-1})	Area (km^2)	Study	Method
**Overall Region**	1974-2004	-0.30 ± 0.10	490	This study	Geodetic
	2004-2018	-0.42 ± 0.11			
	1974-2018	-0.36 ± 0.07			
	2000-2016	-0.43 ± 0.12	-	Brun et al. (2017) [33]	
	1974-2000	-0.27 ± 0.10		King et al. (2019)[42]	
	2000-2016	-0.37 ± 0.11			
	1974-2006	-0.24 ± 0.08		Regettli et al. (2016)[41]	
	2006-2015	-0.45 ± 0.18			
	1974-2000	-0.28 ± 0.11		Zhou et al. (2018) [38]	
	1974-2006	-0.32 ± 0.19		Zhao et al. (2016)[63]	Model based
	1974-2006	-0.40 ± 0.19		Zhao et al. (2016)[64]	Geodetic
**1. G085521E28579N**	1974-2004	-0.26 ± 0.10	14.7	This study	Geodetic
	2004-2018	-0.30 ± 0.11			
	1974-2018	-0.26 ± 0.07			
	2000-2016	-0.27 ± 0.20	-	Brun et al. (2017) [33]	
	1974-2000	-0.26 ± 0.10		King et al. (2019)[42]	
	2000-2016	-0.10 ± 0.11			
**2. G085618E28467N**	1974-2004	-0.28 ± 0.10	14.3	This study	Geodetic
	2004-2018	-0.30 ± 0.11			
	1974-2018	-0.26 ± 0.07			
	2000-2016	-0.23 ± 0.20	-	Brun et al. (2017) [36]	
	1974-2000	-0.16 ± 0.10		King et al. (2019)[42]	
	2000-2016	-0.05 ± 0.11			
**3. G085694E28431N**	1974-2004	-0.40 ± 0.10	34.1	This study	Geodetic
	2004-2018	-0.56 ± 0.11			
	1974-2018	-0.50 ± 0.07			
	2000-2016	-0.65 ± 0.20	-	Brun et al. (2017) [33]	
	1974-2000	-0.33 ± 0.10		King et al. (2019)[42]	
	2000-2016	-0.74 ± 0.11			
**4. G085751E28386N**	1974-2004	-0.23 ± 0.10	22.2	This study	Geodetic
	2004-2018	-0.27 ± 0.11			
	1974-2018	-0.26 ± 0.07			
	2000-2016	-0.23 ± 0.20	-	Brun et al. (2017) [33]	
	1974-2000	-0.16 ± 0.10		King et al. (2019)[42]	
	2000-2016	-0.12 ± 0.11			
**5. G085816E28470N (Kangwure Glacier)**	1974-2004	-0.33 ± 0.10	1.3	This study	Geodetic
	2004-2018	-0.63 ± 0.11			
	1974-2018	-0.41 ± 0.07			
	2000-2016	-0.65 ± 0.20	-	Brun et al. (2017) [33]	
	1974-2000	-		King et al. (2019)[42]	
	2000-2016	-			

	2007-2009	-0.69		Tien et al. (2014)[65]	Glaciological
	1992-2010	-0.43		WGMS[48]	
	1992-2000	-0.39 ± 0.14		Bolch et al. (2012)[66]	Geodetic
**6. G085801E28372N**	1974-2004	-0.23 ± 0.10	25.8	This study	Geodetic
	2004-2018	-0.29 ± 0.11			
	1974-2018	-0.22 ± 0.07			
	2000-2016	-0.26 ± 0.20	-	Brun et al. (2017) [33]	
	1974-2000	-0.22 ± 0.10		King et al. (2019)[42]	
	2000-2016	-0.12 ± 0.11			
**7. G085812E28335N**	1974-2004	-0.36 ± 0.10	22.2	This study	Geodetic
	2004-2018	-0.29 ± 0.11			
	1974-2018	-0.29 ± 0.07			
	2000-2016	-0.31 ± 0.20	-	Brun et al. (2017) [33]	
	1974-2000	-0.45 ± 0.10		King et al. (2019)[42]	
	2000-2016	-0.25 ± 0.11			
**8. G085751E28316N**	1974-2004	-0.31 ± 0.10	34.5	This study	Geodetic
	2004-2018	-0.49 ± 0.11			
	1974-2018	-0.43 ± 0.07			
	2000-2016	-0.53 ± 0.20	-	Brun et al. (2017) [33]	
	1974-2000	-0.27 ± 0.10		King et al. (2019)[42]	
	2000-2016	-0.50 ± 0.11			
**9. G086301E28329N**	1974-2004	-0.13 ± 0.10	14.8	This study	Geodetic
	2004-2018	-0.22 ± 0.11			
	1974-2018	-0.18 ± 0.07			
	2000-2016	-0.18 ± 0.20	-	Brun et al. (2017) [33]	
	1974-2000	-0.11 ± 0.10		King et al. (2019)[42]	
	2000-2016	-0.12 ± 0.11			
**10. G086358E28329N**	1974-2004	-0.11 ± 0.10	24.3	This study	Geodetic
	2004-2018	-0.33 ± 0.11			
	1974-2018	-0.19 ± 0.07			
	2000-2016	-0.30 ± 0.20	-	Brun et al. (2017) [33]	
	1974-2000	-0.19 ± 0.10		King et al. (2019)[42]	
	2000-2016	-0.32 ± 0.10			
**Everest Region**	1970-2007	-0.32 ± 0.08	-	Bolch et al. (2011)[67]	Geodetic
	2000-2008	-0.40 ± 0.25		Nuimura et al. (2012)[68]	
	2000-2016	0.52 ± 0.22		King et al. (2017)[69]	
	2003-2008	-0.31 ± 0.14		ä et al. (2015)[70]	
	1974-2006	-0.40 ± 0.17		Ye et al. (2015)[71]	
	1962-2001	-0.28 ± 0.12		King et al. (2020)[72]	
	2001-2018	-0.35 ± 0.12			

Supplementary Table 25. Comparison of glacier mass balances with other available studies and measurements in the Langtang region.

Name & GLIMS ID	Year	MB (m w.e.a^{-1})	Area (km^2)	Study	Method
**Overall Region**	1964-1974	-0.20 ± 0.09	88	This study	Geodetic
	1974-2004	-0.24 ± 0.10			
	2004-2009	-0.37 ± 0.10			
	2009-2015	-0.55 ± 0.12			
	2015-2017	-0.57 ± 0.13			
	2017-2019	-0.59 ± 0.14			
	1964-2019	-0.32 ± 0.07			
	2000-2016	-0.46 ± 0.12	-	Brun et al. (2017) [33]	
	1974-2000	-0.29 ± 0.10		King et al. (2019)[42]	
	2000-2015	-0.39 ± 0.10			
	1974-2006	-0.21 ± 0.08		Ragettli et al. (2016)[41]	
	2006-2015	-0.38 ± 0.17			
**1. G085670E28312N (Langtang)**	1964-1974	-0.25± 0.09	37.8	This study	Geodetic
	1974-2004	-0.28 ± 0.10			
	2004-2009	-0.49 ± 0.10			
	2009-2015	-0.61 ± 0.12			
	2015-2017	-0.74 ± 0.13			
	2017-2019	-0.86 ± 0.14			
	1964-2019	-0.36 ± 0.07			
	2000-2016	-0.59 ± 0.20	-	Brun et al. (2017) [33]	
	1974-2000	-0.27 ± 0.10		King et al. (2019)[42]	
	2000-2016	-0.44 ± 0.11			
	1974-2006	-0.24 ± 0.08		Ragettli et al. (2016)[41]	
	2006-2015	-0.47 ± 0.13			
	1974-1999	-0.14 ± 0.18		Pellicciotti et al. (2015)[73]	
	1974-2006	-0.30 ± 0.05		Maurer et al. (2019)[74]	
	2000-2016	-0.55 ± 0.06			
**2. G085747E28200N (Langshisha)**	1964-1974	-0.35± 0.09	11.8	This study	Geodetic
	1974-2004	-0.37 ± 0.10			
	2004-2009	-0.48 ± 0.10			
	2009-2015	-0.59 ± 0.12			
	2015-2017	-0.79 ± 0.13			
	2017-2019	-0.84 ± 0.14			
	1964-2019	-0.36 ± 0.07			
	2000-2016	-0.69 ± 0.20	-	Brun et al. (2017) [33]	
	1974-2000	-0.34 ± 0.10		King et al. (2019)[42]	
	2000-2016	-0.68 ± 0.11			
	1974-2006	-0.36 ± 0.08		Ragettli et al. (2016)[41]	
	2006-2015	-0.45 ± 0.18			
	1974-1999	-0.82 ± 0.18		Pellicciotti et al. (2015)[73]	
	1974-2006	-0.34 ± 0.06		Maurer et al. (2019)[74]	

	2000-2016	-0.74 ± 0.09			
**3. G085645E28262N (Shalbachum)**	1964-1974	-0.34 ± 0.09	10.9	This study	Geodetic
	1974-2004	-0.39 ± 0.10			
	2004-2009	-0.39 ± 0.10			
	2009-2015	-0.45 ± 0.12			
	2015-2017	-0.74 ± 0.13			
	2017-2019	-0.78 ± 0.14			
	1964-2019	-0.42 ± 0.07			
	2000-2016	-0.48 ± 0.20	-	Brun et al. (2017)[33]	
	1974-2000	-0.40 ± 0.10		King et al. (2019)[42]	
	2000-2016	-0.44 ± 0.10			
	1974-2006	-0.10 ± 0.08		Ragettli et al. (2016)[41]	
	2006-2015	-0.38 ± 0.18			
	1974-1999	-0.43 ± 0.18		Pellicciotti et al. (2015)[73]	
	1974-2006	-0.30 ± 0.06		Maurer et al. (2019)[74]	
	2000-2016	-0.52 ± 0.07			
**4. G085673E28254N (Ghanna)**	1964-1974	-0.37 ± 0.09	14.8	This study	Geodetic
	1974-2004	-0.41 ± 0.10			
	2004-2009	-0.45 ± 0.10			
	2009-2015	-0.45 ± 0.12			
	2015-2017	-0.67 ± 0.13			
	2017-2019	-0.66 ± 0.14			
	1964-2019	-0.43 ± 0.07			
	2000-2016	-0.59 ± 0.20	-	Brun et al. (2017) [33]	
	1974-2006	-0.43 ± 0.07		Ragettli et al. (2016)[41]	
	2006-2015	-0.39 ± 0.26			
**5. G085618E28236N (Yala)**	1964-1974	-0.25 ± 0.09	24.3	This study	Geodetic
	1974-2004	-0.24 ± 0.10			
	2004-2009	-0.35 ± 0.10			
	2009-2015	-0.44 ± 0.12			
	2015-2017	-0.67 ± 0.13			
	2017-2019				
	1964-2019	-0.31 ± 0.07			
	2000-2016	-0.48 ± 0.20	-	Brun et al. (2017) [33]	
	1974-2000	-0.28 ± 0.07		Ragettli et al. (2016)[41]	
	2000-2016	-0.76 ± 0.24			

Supplementary Table 26. Comparison of mass balances with other available studies and measurements in the Gurla Mandhata region.

Name & GLIMS ID	Year	MB (m w.e.a^{-1})	Area (km^2)	Study	Method
**Overall Region**	1966-2000	-0.12 ± 0.10	73.3	This study	Geodetic
	2000-2011	-0.12 ± 0.10			
	2011-2013	-0.02 ± 0.08			
	2013-2016	-0.20 ± 0.12			
	2016-2018	-0.22 ± 0.12			
	2018-2019	-0.26 ± 0.17			
	2000-2016	-0.15 ± 0.09			
	1966-2019	-0.13 ± 0.09			
	2000-2016	-0.14 ± 0.07		Brun et al. (2017) [33]	
	2000-2013	-0.07 ± 0.11		Holzer et al. (2014)[43]	
**1. G081387E30495N**	1966-2000	-0.24 ± 0.10	1.45	This study	Geodetic
	2000-2016	-0.24 ± 0.09			
	2016-2018	-0.19 ± 0.12			
	2018-2019	-0.24 ± 0.17			
	1966-2019	-0.20 ± 0.09			
	2000-2016	-0.21 ± 0.07		Brun et al. (2017) [33]	
**2. G081379E30472N**	1966-2000	-0.23 ± 0.10	5.2	This study	Geodetic
	2000-2016	-0.22 ± 0.09			
	2016-2018	-0.25 ± 0.12			
	2018-2019	-0.15 ± 0.17			
	1966-2019	-0.23 ± 0.09			
	2000-2016	-0.21 ± 0.07		Brun et al. (2017) [33]	
**3. G081351E30471N (GuNala)**	1966-2000	-0.11 ± 0.10	4.2	This study	Geodetic
	2000-2016	-0.18 ± 0.09			
	2016-2018	-0.14 ± 0.12			
	2018-2019	-0.16 ± 0.17			
	1966-2019	-0.10 ± 0.09			
	2000-2016	-0.17 ± 0.07		Brun et al. (2017) [33]	
**4. G081317E30454N (Gurla/ Naimona’Nyi)**	1966-2000	-0.24 ± 0.10	9.1	This study	Geodetic
	2000-2016	-0.24 ± 0.09			
	2016-2018	-0.27 ± 0.12			
	2018-2019	-0.31 ± 0.17			
	1966-2019	-0.22 ± 0.09			
	2000-2016	-0.21 ± 0.07		Brun et al. (2017) [33]	
	2008-2013	-0.36	-	Tien et al. (2014)[65]	GPS Data
	1973-1983	-0.01 ± 0.15	-	Zhao et al. (2016)[63]	Model Based
	2003-2013	-0.69 ± 0.21			
**5. G081365E30442N**	1966-2000	-0.27 ± 0.10	7.5	This study	Geodetic
	2000-2016	-0.30 ± 0.09			
	2016-2018	-0.26 ± 0.12			

	2018-2019	-0.35 ± 0.17			
	1966-2019	-0.29 ± 0.09			
	2000-2016	-0.33 ± 0.07		Brun et al. (2017) [33]	
**6. G081349E30428N**	1966-2000	-0.21 ± 0.10	1.7	This study	Geodetic
	2000-2016	-0.30 ± 0.09			
	2016-2018	-0.20 ± 0.12			
	2018-2019	-0.31 ± 0.17			
	1966-2019	-0.23 ± 0.09			
	2000-2016	-0.28 ± 0.07		Brun et al. (2017) [33]	
**7. G081307E30424N (NanManuNaNi)**	1966-2000	-0.24 ± 0.10	6.3	This study	Geodetic
	2000-2016	-0.12 ± 0.09			
	2016-2018	-0.24 ± 0.12			
	2018-2019	-0.29 ± 0.17			
	1966-2019	-0.22 ± 0.09			
	2000-2016	-0.14 ± 0.07		Brun et al. (2017) [33]	
**8. G081293E30477N**	1966-2000	-0.03 ± 0.10	1.9	This study	Geodetic
	2000-2016	-0.04 ± 0.09			
	2016-2018	-0.01 ± 0.12			
	2018-2019	-0.05 ± 0.17			
	1966-2019	-0.03 ± 0.09			
	2000-2016	-0.02 ± 0.07		Brun et al. (2017) [33]	
**9. G081296E30455N**	1966-2000	-0.01 ± 0.10	3.2	This study	Geodetic
	2000-2016	-0.08 ± 0.09			
	2016-2018	-0.18 ± 0.12			
	2018-2019	-0.19 ± 0.17			
	1966-2019	-0.03 ± 0.09			
	2000-2016	-0.10 ± 0.07		Brun et al. (2017) [33]	
**10. G081267E30456N (NamuNaNi)**	1966-2000	-0.04 ± 0.10	5.1	This study	Geodetic
	2000-2016	-0.03 ± 0.09			
	2016-2018	-0.06 ± 0.12			
	2018-2019	-0.11 ± 0.17			
	1966-2019	-0.04 ± 0.09			
	2000-2016	-0.02 ± 0.07		Brun et al. (2017) [33]	
**11. G081255E30450N**	1966-2000	-0.07 ± 0.10	2.9	This study	Geodetic
	2000-2016	-0.05 ± 0.09			
	2016-2018	-0.15 ± 0.12			
	2018-2019	-0.19 ± 0.17			
	1966-2019	-0.03 ± 0.09			
	2000-2016	-0.04 ± 0.07		Brun et al. (2017) [33]	
**12. G081237E30450N**	1966-2000	-0.10 ± 0.10	2.0	This study	Geodetic
	2000-2016	-0.04 ± 0.09			
	2016-2018	-0.06 ± 0.12			
	2018-2019	-0.14 ± 0.17			
	1966-2019	-0.07 ± 0.09			
	2000-2016	-0.03 ± 0.07		Brun et al. (2017) [33]	

Supplementary Table 27. Comparison of glacier mass balances with other available studies and measurements in the Muztagh Ata Massif region.

Name & GLIMS ID	Year	MB (m w.e. a^{-1})	Area (km^2)	Study	Method
**Overall Region**	1967-1973	-0.14 ± 0.10	347.6	This study	Geodetic
	1973-2001	-0.01 ± 0.06			
	2001-2009	+0.03 ± 0.10			
	2009-2013	-0.10 ± 0.12			
	2013-2019	-0.12 ± 0.11			
	1967-2019	-0.06 ± 0.07			
	2001-2013	+0.03 ± 0.09			
	2000-2016	+0.07 ± 0.07		Brun et al. (2017) [33]	
	1973-1999	-0.04 ± 0.42	272.7	Holzer et al. (2015)[32]	
	1999-2013	+0.04 ± 0.27			
	1973-1999	-0.15 ± 0.18	375.9	Zhang et al. (2016) [44]	
	1999-2014	-0.13 ± 0.23			
**1. G075225E38255N (Kekesayi)**	1967-1973	-0.14 ± 0.10	84.0	This study	Geodetic
	1973-2001	-0.12 ± 0.06			
	2001-2013	-0.05 ± 0.09			
	2013-2019	-0.19 ± 0.11			
	1967-2019	-0.14 ± 0.07			
	2000-2016	-0.04 ± 0.07		Brun et al. (2017) [33]	
	1973-1999	-0.11 ± 0.42	54.5	Holzer et al. (2015)[32]	
	1999-2013	-0.04 ± 0.27			
	1973-1999	-0.13 ± 0.18	81.2	Zhang et al. (2016) [44]	
	1999-2014	-0.34 ± 0.23			
**2. G075233E38272N**	1967-1973	-0.26 ± 0.10	18.9	This study	Geodetic
	1973-2001	-0.04 ± 0.06			
	2001-2013	-0.07 ± 0.09			
	2013-2019	-0.25 ± 0.11			
	1967-2019	-0.09 ± 0.07			
	2000-2016	-0.05 ± 0.07		Brun et al. (2017) [33]	
	1973-1999	-0.05 ± 0.42	9.2	Holzer et al. (2015)[32]	
	1999-2013	-0.0 ± 0.27			
	1973-1999	-0.47 ± 0.18	13.1	Zhang et al. (2016) [44]	
	1999-2014	+0.01 ± 0.23			
**3. G075175E38297N**	1967-1973	+0.15 ± 0.10	8.7	This study	Geodetic
	1973-2001	-0.16 ± 0.06			
	2001-2013	-0.05 ± 0.09			
	2013-2019	-0.08 ± 0.11			
	1967-2019	-0.03 ± 0.07			
	2000-2016	-0.02 ± 0.07		Brun et al. (2017)[33]	

	1973-1999	-0.15 ± 0.42	6.5	Holzer et al. (2015)[30]	
	1999-2013	+0.18 ± 0.27			
	1973-1999	-0.04 ± 0.18	7.9	Zhang et al. (2016) [44]	
	1999-2014	-0.18 ± 0.23			
**4. G075079E38288N (Kematulejia)**	1967-1973	+0.13 ± 0.10	11.7	This study	Geodetic
	1973-2001	-0.07 ± 0.06			
	2001-2013	+0.09 ± 0.09			
	2013-2019	-0.09 ± 0.11			
	1967-2019	-0.03 ± 0.07			
	2000-2016	+0.07 ± 0.07		Brun et al. (2017) [33]	
	1973-1999	-0.05 ± 0.42	8.4	Holzer et al. (2015)[32]	
	1999-2013	+0.06 ± 0.27			
	1973-1999	-0.04 ± 0.18	9.3	Zhang et al. (2016) [44]	
	1999-2014	-0.22 ± 0.23			
**5. G075084E38279N**	1967-1973	+0.04 ± 0.10	15.1	This study	Geodetic
	1973-2001	+0.02 ± 0.06			
	2001-2013	+0.06 ± 0.09			
	2013-2019	+0.02 ± 0.11			
	1967-2019	+0.02 ± 0.07			
	2000-2016	+0.10 ± 0.07		Brun et al. (2017) [33]	
	1999-2013	+0.17 ± 0.27	11.1	Holzer et al. (2015)[32]	
**6. G075077E38257N (Kalaxiong)**	1967-1973	-0.09 ± 0.10	23.7	This study	Geodetic
	1973-2001	-0.04 ± 0.06			
	2001-2013	+0.07 ± 0.09			
	2013-2019	-0.01 ± 0.11			
	1967-2019	-0.03 ± 0.07			
	2000-2016	+0.08 ± 0.07		Brun et al. (2017) [33]	
	1973-1999	-0.04 ± 0.42	15.4	Holzer et al. (2015)[32]	
	1999-2013	+0.04 ± 0.27			
	1973-1999	-0.11 ± 0.18	19.9	Zhang et al. (2016) [44]	
	1999-2014	+0.03 ± 0.23			
**7. G075058E38248N (Muztagh Ata Glacier No. 15)**	1967-1973	+0.03 ± 0.10	0.91	This study	Geodetic
	1973-2001	+0.13 ± 0.06			
	2001-2013	+0.17 ± 0.09			
	2013-2019	+0.15 ± 0.11			
	1967-2019	+0.11 ± 0.07			
	2000-2016	+0.16 ± 0.07		Brun et al. (2017) [33]	
	1999-2013	+0.21 ± 0.27	0.9	Holzer et al. (2015)[32]	
	1973-1999	+0.05 ± 0.18	1.1	Zhang et al. (2016) [44]	
	2006-2010	+0.20	-	Yao et al. (2012)[23]	Glaciological
**8. G075071E38240N**	1967-1973	-0.12 ± 0.10	8.9	This study	Geodetic
	1973-2001	+0.01 ± 0.06			
	2001-2013	+0.14 ± 0.09			
	2013-2019	-0.15 ± 0.11			
	1967-2019	-0.01 ± 0.07			

	2000-2016	+0.17 ± 0.07		Brun et al. (2017) [33]	
	1999-2013	+0.14 ± 0.27	8.1	Holzer et al. (2015)[32]	
	1973-1999	+0.03 ± 0.18	5.8	Zhang et al. (2016) [44]	
	1999-2014	-0.05 ± 0.23			
**9. G075092E38214N (Kuosikulake)**	1967-1973	-0.18 ± 0.10	21.2	This study	Geodetic
	1973-2001	+0.01 ± 0.06			
	2001-2013	+0.10 ± 0.09			
	2013-2019	-0.23 ± 0.11			
	1967-2019	-0.02 ± 0.07			
	2000-2016	+0.07 ± 0.07		Brun et al. (2017) [33]	
	1973-1999	0.0 ± 0.42	12.8	Holzer et al. (2015)[32]	
	1973-1999	-0.09 ± 0.18	16.1	Zhang et al. (2016)[44]	
	1999-2014	+0.16 ± 0.23			
**10. G075075E38189N**	1967-1973	-0.02 ± 0.10	2.9	This study	Geodetic
	1973-2001	+0.02 ± 0.06			
	2001-2013	+0.05 ± 0.09			
	2013-2019	-0.04 ± 0.11			
	1967-2019	-0.00 ± 0.07			
	2000-2016	+0.09 ± 0.07		Brun et al. (2017) [33]	
	1973-1999	+0.02 ± 0.42	2.5	Holzer et al. (2015)[32]	
	1999-2013	+0.03 ± 0.27			
**11. G075156E38175N (Kuokuosele)**	1967-1973	-0.15 ± 0.10	23.0	This study	Geodetic
	1973-2001	+0.06 ± 0.06			
	2001-2013	+0.11 ± 0.09			
	2013-2019	+0.10 ± 0.11			
	1967-2019	+0.03 ± 0.07			
	2000-2016	+0.12 ± 0.07		Brun et al. (2017) [33]	
	1973-1999	+0.09 ± 0.42	16.4	Holzer et al. (2015)[32]	
	1999-2013	+0.08 ± 0.27			
	1999-2014	+0.03 ± 0.23	20.3	Zhang et al. (2016) [44]	
**12. G075171E38163N**	1967-1973	-0.17 ± 0.10	8.5	This study	Geodetic
	1973-2001	-0.05 ± 0.06			
	2001-2013	-0.02 ± 0.09			
	2013-2019	-0.02 ± 0.11			
	1967-2019	-0.07 ± 0.07			
	2000-2016	+0.01 ± 0.07		Brun et al. (2017) [33]	
	1973-1999	-0.05 ± 0.42	5.8	Holzer et al. (2015)[32]	
	1999-2013	-0.16 ± 0.27			
	1999-2014	-0.15 ± 0.23	8.0	Zhang et al. (2016) [44]	

Supplementary Table 28. Comparison of the mass balance of surge-type and non-surge-type glaciers in the Ak-Shirak and Muztagh-Ata regions.

**Ak-Shirak Region**				
**Time period**	**Mass balance (m w.e. a^{-1}) surge-type glaciers (N1 = 9)**	**Mass balance (m w.e. a^{-1}) non-surge-type glaciers (N2 = 168)**	**Mass balance (m w.e. a^{-1}) of lake-terminating (Petrov) glacier (N3 = 1)**	**Overall Mass balance (m w.e. a^{-1})**
**1964-1973**	-0.27 ± 0.16	-0.32 ± 0.16	-0.59 ± 0.16	-0.30 ± 0.16
**1973-1980**	-0.22 ± 0.18	-0.28 ± 0.18	-0.39 ± 0.18	-0.26 ± 0.18
**1980-2002**	-0.62 ± 0.10	-0.51 ± 0.10	-0.68 ± 0.10	-0.54 ± 0.10
**2002-2009**	-0.45 ± 0.11	-0.25 ± 0.11	-0.31 ± 0.11	-0.31 ± 0.11
**2009-2015**	-0.26 ± 0.13	-0.32 ± 0.13	-0.37 ± 0.13	-0.30 ± 0.13
**2015-2017**	-0.35 ± 0.30	-0.26 ± 0.30	-0.39 ± 0.30	-0.32 ± 0.30
**2017-2019**	-0.38 ± 0.23	-0.25 ± 0.23	-0.40 ± 0.23	-0.29 ± 0.23
**1964-2019**	-0.43 ± 0.07	-0.39 ± 0.07	-0.68 ± 0.07	-0.40 ± 0.07
**Muztagh-Ata Region**				
	**(N1 = 3)**	**(N2 = 163)**		
**1967-1973**	-0.15 ± 0.10	-0.13 ± 0.10		-0.14 ± 0.10
**1973-2001**	+0.04 ± 0.06	-0.02 ± 0.06		-0.01 ± 0.06
**2001-2009**	+0.07 ± 0.10	+0.02 ± 0.10		+0.03 ± 0.10
**2009-2013**	+0.04 ± 0.12	-0.12 ± 0.12		-0.10 ± 0.12
**2013-2019**	-0.12 ± 0.09	-0.12 ± 0.09		-0.12 ± 0.09
**1967-2019**	+0.01 ± 0.07	-0.07 ± 0.07		-0.06 ± 0.07

Supplementary Table 29: Comparison of Elevation difference in different time period using regional mean hypsometry and regional median hypsometry gap filling methods for Ak-Shirak region

Time period								
**Gap Filling Method**	**1964-1973**	**1973-1980**	**1980-2002**	**2002-2009**	**2009-2015**	**2015-2017**	**2017-2019**	**1964-2019**
**Mean Hypsometry**	-3.30 ± 1.92	-2.05 ± 1.70	-13.93 ± 3.00	-2.54 ± 1.04	-2.13 ± 1.05	-0.73 ± 0.84	-0.64 ± 0.60	-26.04 ± 5.31
**Median Hypsometry**	-3.21 ± 1.92	-2.15 ± 1.70	-13.98 ± 3.00	-2.59 ± 1.04	-2.12 ± 1.05	-0.76 ± 0.84	-0.68 ± 0.60	-26.13 ± 5.31

Supplementary Table 30: Mean and median elevation change considering outlier outside ±100 m or ±150 m for Ak-Shirak and Northern Tien Shan region

	**Ak-Shirak**		**Northern Tien Shan**	
	Mean dh (m)	Median dh (m)	Mean dh (m)	Median dh (m)
Dh > 100 m	-23.76	-22.07	-26.90	-23.25
Dh >150 m	-25.90	-22.58	-27.40	-23.39

Supplementary References

1. McDonald, R. A. CORONA-success for space reconnaissance, a look into the Cold War, and a revolution for intelligence. *Photogramm. Eng. Remote Sens.* **61**, 689–720 (1995).
2. Galiatsatos, N., Donoghue, D. N. M. & Philip, G. High resolution elevation data derived from stereoscopic CORONA imagery with minimal ground Control: an approach using IKONOS and SRTM Data. *Photogramm. Eng. Remote Sens*. **74**, 1093–1106 (2008).
3. Altmaier, A. & Kany, C. Digital surface model generation from CORONA satellite images. *ISPRS J. Photogramm*. *Remote Sens*. **56**, 221–235 (2002).
4. Pieczonka, T., Bolch, T. & Buchroithner, M. F. Generation and evaluation of multitemporal digital terrain models of the Mt. Everest area from different optical sensors. *ISPRS J. Photogramm*. **66**, 927–940 (2011).
5. Sohn, H. G., Kim, G. H. & Yom, J. H. Mathematical modelling of historical reconnaissance Corona KH-4B imagery. *Photogramm. Rec.* **19** (105), 51-66 (2004).
6. Dashora, A., Lohani, B. & Malik, J. N. A repository of earth resource information - CORONA satellite programme. *Curr. Sci*. **92**, 926–932 (2007).
7. Goerlich, F., Bolch, T., Mukherjee, K. & Pieczonka, T. Glacier mass loss during the 1960s and 1970s in the Ak-Shirak range (Kyrgyzstan) from multiple stereoscopic Corona and Hexagon imagery. *Remote Sens.* **9**, 275-292 (2017).
8. Pieczonka, T. & Bolch, T. Region-wide glacier mass budgets and area changes for the Central Tien Shan between ~1975 and 1999 using Hexagon KH-9 imagery. *Glob. Planet. Change*. **128**, 1-13 (2015).
9. Surazakov, A. & Aizen, V. Positional accuracy evaluation of declassified Hexagon KH-9 mapping camera imagery. *Photogramm. Eng. Remote Sens.* **76**, 603–608 (2010).
10. Khalsa, S. J. R., Dyurgerov, M. B., Khromova, T., Raup, B. H. & Barry, R. G. Space-based mapping of glacier changes using ASTER and GIS tools. *IEEE Trans. Geosci. Remote Sens.* **42**, 2177-2183 (2004).
11. Maliet, E. SPOT-6 and SPOT-7: Offering SPOT data continuity. *In 64th International Astronautical Congress (IAC 2013)*, Beijing, China, 23-27 September 2013.
12. Zhou, Y. *et al.* Quantifying glacier mass change and its contribution to lake growths in central Kunlun during 2000-2015 from multi-source remote sensing data. *J. Hydrol.* **570**, 38-50 (2019).

13. Gleyzes, M. A., Perret, L. & Kubik, P. Pleiades system architecture and main performances. *In International Archives of the Photogrammetry Remote Sensing and Spatial Information Sciences Congress*, Melbourne, Australia, 2012.

14. Durand, A., Michel, J., de Franchis, C., Allenbach, B. & Giros, A. Assessment of three DSM generation approaches using Pleiades-HR data. *In 33rd EARSeL Symposium towards Horizon 2020*, Matera, Italy, 2013.

15. Krieger, G. *et al.* TanDEM-X: a satellite formation for high resolution SAR interferometry. *IEEE Trans. Geosci. Remote Sens.* **45**, 3317-3341 (2007).

16. Buckreuss, S., Werninghaus, R. & Pitz, R. W. The German satellite mission TerraSAR-X. *IEEE Aero. El. Sys. Mag.* **24**, 4-9 (2009).

17. Wang, X., Tolksdorf, V., Otto, M. & Scherer, D. WRF-based dynamical downscaling of ERA5 reanalysis data for High Mountain Asia: Towards a new version of the High Asia Refined Analysis. *Int. J. Climate*. 1-20 (2020), doi.: https://doi.org/10.1002/joc.6686.

18. Maussion, F. et al. Precipitation seasonality and variability over the Tibetan Plateau as resolved by the High Asia Reanalysis. *J. Climate.*, **27**, 1910-1927 (2014).

19. Bolch, T. Climate change and glacier retreat in northern Tien Shan (Kazakhstan/Kyrgyzstan) using remote sensing data. *Global. Planet. Change.* **56**, 1-12 (2007).

20. Aizen, V. B., Aizen, E. M. & Melack, J. M. Climate, snow cover, glaciers, and runoff in the Tien Shan, central Asia. *J. Am. Water. Resour. Assoc.* **31**, 1113–1129 (1995).

21. Ozmonov, A., Bolch, T., Xi, C., Kurban, A. & Guo, W. Glacier characteristics and changes in the Sary-Jaz river Basin (Central Tien Shan) 1990–2010. *Remote. Sens. Lett.* **4**, 725–734 (2013).

22. Kronenberg, M. *et al.* Mass-balance reconstruction for Glacier No. 354, Tien Shan, from 2003 to 2014. *Ann. Glaciol.* **57**, 92–102 (2015).

23. Yao, T. *et al*. Different glacier status with atmospheric circulations in Tibetan Plateau and surroundings. *Nat. Clim. Change.* **2**, 663–667 (2012).

24. Liu, S. *et al*. Mass balance sensitivity to climate change of the Glacier No. 1 at the headwaters of the Ürümqi river, Tianshan mountains. *J. Glaciol. Geocryol.* **20**, 9–13 (1998).

25. Zhang, Z., Jiang, L., Liu, L., Sun, Y. & Wang, H. Annual glacier-wide mass balance (2000-2016) of the interior Tibetan Plateau reconstructed from MODIS albedo products. *Remote Sens.* **10**, 1031-1051 (2018).

26. Spiess, M., Maussion, F., Möller, M., Scherer, D. & Schneider, C. MODIS Derived Equilibrium Line Altitude Estimates for Purogangri Ice Cap, Tibetan Plateau, and their Relation to Climatic Predictors (2001–2012). *Geog. Ann. Series A Phys. Geog.* **97**, 599–614 (2015).
27. Kang, S. *et al.* Correspondence: Early onset of rainy season suppresses glacier melt: a case study on Zhadang glacier, Tibetan Plateau. *J. Glaciol.* **55**, 755–758 (2009).
28. Bolch, T. *et al.* A glacier inventory for the western Nyainqentanglha Range and Nam Co Basin, Tibet, and glacier changes 1976-2009. *Cryosphere*. **4**, 419–433 (2010).
29. Caidong, C. & Sorteberg, A. Modelled mass balance of the Xibu glacier, Tibetan Plateau: sensitivity to climatic change. *J. Glaciol.* **56**, 235-248 (2010).
30. Chen, X., Cui, P., Yong, L., Yang, Z. & Qi, Y. Changes in glacial lakes and glaciers of post-1986 in the Poiqu River basin, Nyalam, Xizang (Tibet). *Geomorphology*. **88**, 298–311 (2007).
31. Owen, L. A., Yi, C., Finkel, R. C. & Davis, N. K. Quaternary glaciation of Gurla Mandhata (Naimon'anyi). *Quat. Sci. Rev.* **29**, 1817-1830 (2010).
32. Holzer, N. *et al*. Four decades of glacier variations at Muztagh Ata (eastern Pamir): a multi-sensor study including Hexagon KH-9 and Pléiades data. *Cryosphere*. **9**, 2071–2088 (2015).
33. Brun, F., Berthier, E., Wagnon, P., Kääb, A. & Teichler, D. A spatially resolved estimate of High Mountain Asia glacier mass balances from 2000 to 2016. *Nat. Geosci*. **10**, 668-673 (2017).
34. Mukherjee, K. *et al.* Surge-type glaciers in the Tien Shan (Central Asia). *Arct. Antarct. Alp. Res.* **49**, 147–171 (2017).
35. Jamieson, S. S. R., Ewertowski, M. W. & Evana, D. J. A. Rapid advance of the mountain glaciers in response to mine-related debris loading. *J. Geophys. Res. Earth Surf.* **120**, 1418-1435 (2015).
36. Lei, Y., Yao, T., Yi, C. & Wang, W. Glacier mass loss induced the rapid growth of Linggo Co on the central Tibetan Plateau. *J. Glaciol.* **58**, 177–184 (2012).
37. Neckel, N., Braun, A., Kropáček, J. & Hochschild, V. Recent mass balance of the Purogangri Ice Cap, central Tibetan Plateau, by means of differential X-band SAR interferometry. *Cryosphere.* **7**, 1623–1633 (2013).
38. Zhou, Y., Li, Z., Li, J., Zhao, R. & Ding, X. Glacier mass balance in the Qinghai–Tibet Plateau and its surroundings from the mid-1970s to 2000 based on Hexagon KH-9 and SRTM DEMs. *Remote Sens. Environ.* **210**, 96–112 (2018).

39. Wu, H., Wang, N., Jiang, X. & Guo Z. Variation in water level and glacier mass balance in Nam Co lake, Nyainqentanglha range, Tibetan Plateau, based on ICESat data for 2003-2009. *Ann. Glaciol.* 55 (66), 239-247 (2014).
40. Neckel, N., Kropacek, J., Bolch, T., Hochschild, V. Glacier mass changes on the Tibetan Plateau 2003-2009 derived from ICESat laser altimetry measurements. *Environ. Res. Lett.* 9 014009 (2014).
41. Ragettli, S., Bolch, T. & Pellicciotti, F. Heterogeneous glacier thinning patterns over the last 40 years in Langtang Himal, Nepal. *Cryosphere*. **10** (5), 2075–2097 (2016).
42. King, O., Bhattacharya, A., Bhambri, R. & Bolch, T. Glacial lakes exacerbate Himalayan glacier mass loss. *Sci. Rep*. **9**, 18145 (2019).
43. Holzer, N., Buchroithner, M. F., Gourmelen, N. & Colin, J. Suitability of a Pléiades VHR Digital Surface Model for glacier mass balance estimates at Mt. Gurla Mandhata and Mt. Geladandong (China). *In proceedings Pléiades Days 2014*, Toulouse, France, April 2014.
44. Zhang, Z. *et al.* Mass Change of Glaciers in Muztagh Ata–Kongur Tagh, Eastern Pamir, China from 1971/76 to 2013/14 as Derived from Remote Sensing Data. *PLOS ONE*, **11** (1) (2016).
45. McNabb, R., Nuth, C., Kääb, A. & Girod, L. Sensitivity of glacier volume change estimation to DEM void interpolation. *Cryosphere*. **13**, 895-910 (2019).
46. McNabb, R., Nuth, C., Kääb, A. & Girod, L. Sensitivity of glacier volume change estimation to DEM void interpolation. *Cryosphere*. **13**, 895-910 (2019).
47. Hagg, W. J., Braun, L. N., Uvarov, V. N. & Makarevich, K. G. A comparison of three methods of mass balance determination in the Tuyuksu glacier region, Tien Shan, Central Asia. *J. Glaciol.* **50**, 505-510 (2004).
48. WGMS 2019, Fluctuations of Glaciers Database. World Glacier Monitoring Service, Zurich, Switzerland. Online access: http://dx.doi.org/10.5904/wgms-fog-2019-12 (2019).
49. Kapitsa, V. *et al.* Assessment of changes in mas balance of the Tuyuksu gropu of glaciers, Northern Tien Shan, between 1958 and 2016 using ground-based observations and Pleiades satellite imagery. *Front. Earth Sci.* 10.3389/feart.2020.00259 (2020).
50. Aizen, V. B., Kuzmichenok, V. A., Surazakov, A. B. & Aizen, E. M. Glacier changes in the Tien Shan as determined from topographic and remotely sensed data. *Global. Planet. Change.* **56**, 328–340 (2007).

51. Petrakov, D. A., Lavrientiev, I, I., Kovalenko, N. V. & Usubaliev, R. A. Snow Cover and Glaciers Ice Thickness, Volume and Current Changes of the Sary-Tor Glacier Area (Ak-Shyirak Massif, Inner Tian Shan). *Earth's Cryosphere*. **18**, 83–91 (2014).
52. Lei, Y., Yao, T., Yi, C. & Wang, W. Glacier mass loss induced the rapid growth of Linggo Co on the central Tibetan Plateau. *J. Glaciol.* **58**, 177–184 (2012).
53. Huintjes, E. *et al.* Evaluation of a Coupled Snow and Energy Balance Model for Zhadang Glacier, Tibetan Plateau, Using Glaciological Measurements and Time-Lapse Photography. *Arct. Antarct.Alp. Res.* **47**, 573-590 (2015).
54. Liu, L. e*t al.* Glacier elevation changes (2012–2016) of the Purogangri Ice Field on the Tibetan Plateau derived from bi-temporal TanDEM-X InSAR data. *Int. J. Remote Sens.* **37**, 5687–5707 (2016).
55. Li, G. & Lin, H. Recent decadal glacier mass balances over the Western Nyainqentanglha Mountains and the increase in their melting contribution to Nam Co Lake measured by differential bistatic SAR interferometry. *Global. Planet. Change.* **149**, 177–190 (2017).
56. Kang, S. *et al*. Dramatic loss of glacier accumulation area on the Tibetan Plateau revealed by ice core tritium and mercury records. *Cryosphere*. **9** (3), 1213–1222 (2015).
57. Gardelle, J., Berthier, E., Arnaud, Y. & Kääb, A. Region-wide glacier mass balances over the Pamir-Karakoram-Himalaya during 1999-2011. *Cryosphere.* **7**, 1263-1286 (2013).
58. Zhang, Q. & Zhang, G. Glacier elevation changes in the western Nyainqentanglha Range of the Tibetan Plateau as observed by TerraSAR-X/TanDEM-X images. *Remote Sens. Lett.* **8**, 1142–1151 (2017).
59. Gardner, S. *et al*. A reconciled estimate of glacier contributions to sea level rise: 2003 to 2009. *Science* **340**, 852-857 (2013).
60. Zhu, M. *et al.* Energy- and mass-balance comparison between Zhadang and Parlung No. 4 glaciers on the Tibetan Plateau. *J. Glaciol.* **61**, 595-607 (2015).
61. Zhang, G. *et al*. Energy and mass balance of Zhadang glacier surface, central Tibetan Plateau. *J. Glaciol.* **59** (213), 137-148 (2013).
62. Yu, W. *et al.* Different region climate regimes and topography affect the changes in area and mass balance of glaciers on the north and south slopes of the same glacierized massif (the West Nyainqentanglha Range, Tibetan Plateau). *J. Hydrol.* **495**, 64–73 (2013).

63. Zhao, H., Yang, W., Yao, T., Tian, L. & Xu, B. Dramatic mass loss in extreme high-elevation areas of a western Himalayan glacier: observations and modeling. *Sci. Rep.* **6,** 30706 (2016).
64. Zhao, L., Ding, R. & Moore, J. C. The High Mountain Asia glacier contribution to sea-level rise from 2000 to 2050, *Ann.Glaciol.* 57, 223–231 (2016).
65. Tian, L. *et al.* Direct measurement of glacier thinning on the southern Tibetan Plateau (Gurenhekou, Kangwure and Naimona'Nyi glaciers). *J. Glaciol.* **60**, 879–888 (2014).
66. Bolch, T. *et al*. The state and fate of Himalayan glaciers. *Science* **336**, 310–314 (2012).
67. Bolch, T., Pieczonka, T. & Benn, D. I. Multi-decadal mass loss of glaciers in the Everest area (Nepal Himalaya) derived from stereo imagery. *Cryosphere.* **5**, 349-358 (2011).
68. Nuimura, T., Fujita, K., Yamaguchi, S., & Sharma, R.R. Elevation changes of glaciers revealed by multitemporal digital elevation models calibrated by GPS survey in the Khumbu region, Nepal Himalaya, 1992–2008. *J. Glaciol.* 58, 648-656 (2012).
69. King, O., Quincey, D.J., Carrivick, J.L., & Rowan, A.V. Spatial variability in mass loss of glaciers in the Everest region, central Himalayas, between 2000 and 2015. *Cryosphere*, 11, 407–426 (2017).
70. Kääb, A., Treichler, D., Nuth, C. & Berthier, E. Brief Communi-cation: Contending estimates of 2003–2008 glacier mass balanceover the Pamir–Karakoram–Himalaya. *Cryosphere*, 9, 557–564 (2015).
71. Ye, Q. *et al.* Glacier mass changes in Rongbukcatchment on Mt. Qomolangma from 1974 to 2006 based on to-pographic maps and ALOS PRISM data. *J. Hydrol.* 530, 273–280 (2015).
72. King, O. *et al.* Six decades of glacier mass changes around Mt. Everest are revealed by historical and contemporary images. *One Earth,* **3** (5), 608-620 (2020).
73. Pellicciotti, F. *et al.* Mass balance changes of the debris-covered glaciers in the Langtang Himal, Nepal, from 1974 to 1999. *J. Glaciol.* 61(226), 373-386 (2015)
74. Maurer, J. M., Schaefer, J. M., Rupper, S. & Corley, A. Acceleration of ice loss across the Himalayas over the past 40 years. *Sci. Adv.* **5**, 6 (2019).
